# Data-independent acquisition boosts quantitative metaproteomics for deep characterization of gut microbiota

**DOI:** 10.1038/s41522-023-00373-9

**Published:** 2023-01-24

**Authors:** Jinzhi Zhao, Yi Yang, Hua Xu, Jianxujie Zheng, Chengpin Shen, Tian Chen, Tao Wang, Bing Wang, Jia Yi, Dan Zhao, Enhui Wu, Qin Qin, Li Xia, Liang Qiao

**Affiliations:** 1 Department of Chemistry, Shanghai Stomatological Hospital, Fudan University, 200000 Shanghai, China; 2 Department of Core Facility of Basic Medical Sciences, and Department of Psychiatry of Shanghai Mental Health Center, Shanghai Jiao Tong University School of Medicine, 200000 Shanghai, China; 3 Shanghai Omicsolution Co., Ltd, 201100 Shanghai, China; 4 Changhai Hospital, The Naval Military Medical University, 200433 Shanghai, China; 5 College of Food Science and Technology, Shanghai Ocean University, 201306 Shanghai, China; 6 Present Address: ZJU-Hangzhou Global Scientific and Technological Innovation Center, Zhejiang University, 311200 Hangzhou, China

**Keywords:** Microbiome, Biological techniques

## Abstract

Metaproteomics can provide valuable insights into the functions of human gut microbiota (GM), but is challenging due to the extreme complexity and heterogeneity of GM. Data-independent acquisition (DIA) mass spectrometry (MS) has been an emerging quantitative technique in conventional proteomics, but is still at the early stage of development in the field of metaproteomics. Herein, we applied library-free DIA (directDIA)-based metaproteomics and compared the directDIA with other MS-based quantification techniques for metaproteomics on simulated microbial communities and feces samples spiked with bacteria with known ratios, demonstrating the superior performance of directDIA by a comprehensive consideration of proteome coverage in identification as well as accuracy and precision in quantification. We characterized human GM in two cohorts of clinical fecal samples of pancreatic cancer (PC) and mild cognitive impairment (MCI). About 70,000 microbial proteins were quantified in each cohort and annotated to profile the taxonomic and functional characteristics of GM in different diseases. Our work demonstrated the utility of directDIA in quantitative metaproteomics for investigating intestinal microbiota and its related disease pathogenesis.

## Introduction

The human body is composed not only of human cells but also complex and dynamic populations of microorganisms that inhabit various body sites, including the gastrointestinal tract^1^. Through host-microbiota interactions, the microbes are closely associated with human health and disease^2^. It has been estimated that the human-associated microbiota has a genetic composition that is over 100 times the amount of the human genome^3,4^. The development of genome sequencing technologies has accelerated the study of gut microbiota (GM). Metagenomics can provide a comprehensive view on the taxonomic composition of GM, but is limited in functional analysis of microbiome, e.g. identifying activated bacterial metabolic pathways^5^.

Metaproteomics based on liquid chromatography–tandem mass spectrometry (LC-MS/MS) has shown the capability to provide deep function information regarding the dynamic host-microbiota interactions^2,5,6^. Due to the extreme complexity and heterogeneity of gut metaproteome, accurate protein identification and quantification in metaproteome is still a severe challenge compared to the conventional proteomics of a single organism. Most current metaproteomic studies are based on label-free quantification (LFQ) using the data-dependent acquisition (DDA) approach^7–11^. In a typical LFQ-DDA analysis, protein identification is performed by database searching of peptide fragment spectra, while protein quantification is based on precursor ion intensities^9,10^ or the numbers of identified spectra^8^. Quantification accuracy of LFQ-DDA is affected by the precursor selection procedure, which constitutes a stochastic element, resulting in the “missing value” problem^12^. The “missing value” problem is more significant in metaproteomics compared to conventional single-organism proteomics due to the significantly enhanced sample heterogeneity, complexity and dynamic range. To overcome this issue, isobaric labeling techniques, including the tandem mass tag (TMT), has been introduced to metaproteomics, enabling multiplex quantification in one MS analysis^13–15^. Currently, commercially available TMT labeling reagents allow up to 18-plex experiments^16^, which can be used for small-scale quantitative metaproteomic cohort studies. In a TMT analysis, protein quantification is based on the reporter ions generated by the isobaric labels after fragmentation. Co-isolation of multiple labeled peptides can lead to errors in the relative quantification. Due to the high complexity of microbiota sample, the co-isolation issue is more significant in metaproteomics, which can be partially solved by further fragmentation with an additional isolation step (MS3) on special instruments^17^.

During the past years, data-independent acquisition (DIA) methods have emerged, which can systematically record fragmentation information of all precursor ions within defined isolation windows. In a typical DIA analysis, peptide abundances are measured by targeted extraction of quantitative signals using a spectral library of known peptides^18^, wherein the spectral library has great impacts on the analysis results^19^. The strategy is known as the peptide-centric DIA analysis or library-based DIA. As an alternative, DIA data can also be searched against a proteome sequence database directly through spectrum deconvolution^20^, which is known as the spectrum-centric DIA analysis or library-free DIA. DIA has shown outstanding performance in conventional proteomics with increased proteome coverage, reproducibility, and accuracy in quantification^21–24^. Despite the promising applications, DIA is still in the early stage of development in the field of metaproteomics^25–27^. In 2020, Aakko et al. provided a proof of concept for DIA metaproteomics and demonstrated its technical feasibility in GM metaproteomics using laboratory-assembled microbial mixtures as well as human fecal samples^26^. In the same year, we also applied library-based DIA to real clinical gut metaproteome samples and quantified more than 30,000 proteins^25^. More recently, Pietilä et al. introduced library-free DIA for metaproteomic analysis of complex microbial samples, which circumvents the initial DDA-originated limitations in peptide identification that hamper approaches using a DDA-based spectral library^27^. To date, the performance of DIA metaproteomics has not been compared with other quantification strategies in a systematic way.

Herein, we applied library-free DIA (directDIA)-based metaproteomics and compared directDIA with the commonly used MS-based quantification strategies, i.e., LFQ-DDA, DDA with TMT labeling, and the library-based DIA on a simulated microbial community and feces samples spiked with bacteria with known ratios, demonstrating the superior performance of directDIA based on a comprehensive consideration of proteome coverage, as well as accuracy and precision in quantification. Then, we applied the directDIA workflow to characterize human GM in real clinical feces samples, including cohorts of pancreatic cancer (PC) and mild cognitive impairment (MCI). About 70,000 microbial proteins were quantified in each cohort and annotated to profile the taxonomic and functional characteristics of GM. We expect that our work will promote the application of DIA quantitative metaproteomics to investigate intestinal microbiota and its related disease pathogenesis.

## Results

### Benchmarking quantification strategies using simulated microbial communities

Microbial mixtures with known composition have been used as benchmarking samples for performance evaluation of metaproteomics methods. We constructed a simulated microbial community consisting of 12 species of bacteria, including 9 anaerobic and 3 aerobic bacteria species commonly found in GM (Supplementary Table 1). The number of species in the mixtures was set in line with previous studies of benchmarking metaproteomic methods, which used lab-assembled microbial mixtures composed by 5–12 species^26–29^. The concentration of each individual species was determined by plate counting and optical density measurement at the wavelength of 595 nm. Then the species were mixed to form three samples with expected cell number ratios (Supplementary Table 2). These samples with “ground truth” relative quantities allowed us to evaluate different MS quantification strategies and data analysis methods.

**Fig. 1:**
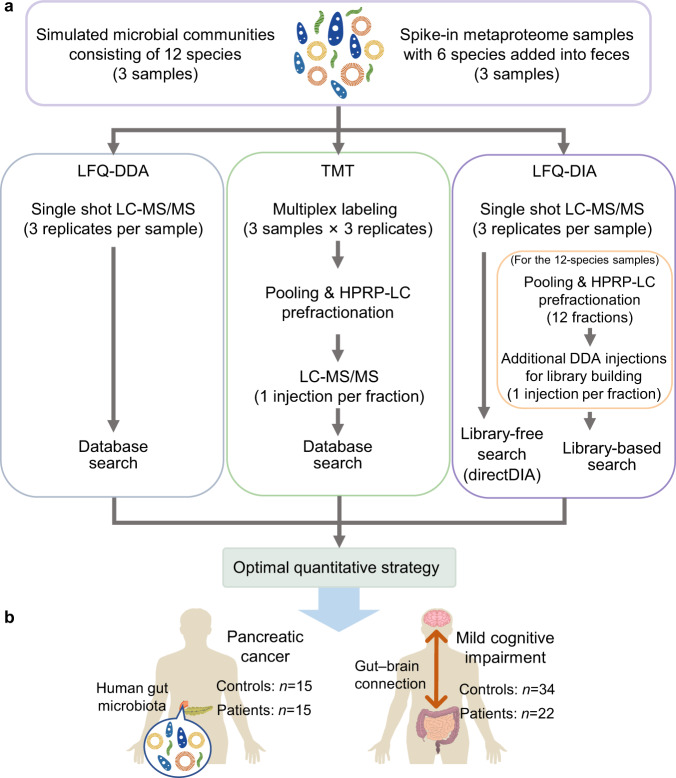
Overview of the study. **a** Benchmarking label-free quantification by data-dependent acquisition (LFQ-DDA) and data-independent acquisition (LFQ-DIA), as well as isobaric tandem mass tag (TMT)-based quantification, on simulated microbial communities consisting of 12 species of bacteria and feces samples spiked with 6 species. **b** Profiling human gut metaproteome in clinical samples of pancreatic cancer patients and mild cognitive impairment patients.

Three current mainstream MS quantification strategies, i.e., LFQ-DDA, LFQ-DIA, and TMT, were tested on the simulated microbial communities (Fig. 1a and Supplementary Fig. 1). DDA and DIA injections were performed on individual samples with three technical replicates (repeated injections) per sample, which was intended to assess the variability of the MS acquisition and data analysis. The LFQ-DDA and LFQ-DIA quantification was based on MS1 precursor intensities and MS2 fragment intensities, respectively. For TMT, three technical replicates were prepared for each sample, and the 3 samples × 3 replicates were labeled by TMT 10-plex reagents (one report ion channel per sample replicate). Thus, the measured variability also involved the TMT labeling process. The labeled samples were mixed and then divided into 16 fractions by high-pH reversed-phase (HPRP) LC. Each fraction was analyzed with one LC-MS/MS injection. A second round of fragmentation (MS3) was performed to generate the reporter ions for quantitative analysis^30^. The MS3-based TMT was employed to minimize the influence of co-isolation issue. The correlation between raw file names and the sample compositions is shown in Supplementary Table 3.

**Fig. 2:**
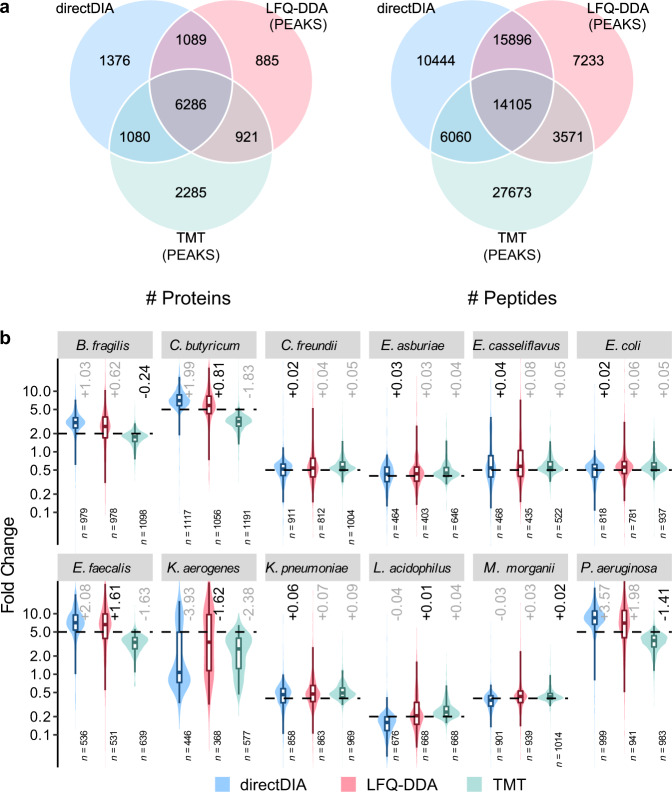
Performance evaluation of directDIA, LFQ-DDA, and TMT on the simulated microbial community of 12 species. **a** Overlap of proteins and peptides shared in at least 2/3 replicates in each sample group by different methods. **b** Measured fold change (FC) values of protein abundance between sample 3 (as numerator) and sample 1 (as denominator) for each species calculated based on the average of the replicates of each sample. Only proteins quantified in at least 2/3 replicates of each sample group and uniquely belonging to one species were taken into consideration. Numbers (*n*) of quantified proteins are indicated for each species. The boxes mark the first and third quantile and the lines inside the boxes mark the median; the whiskers mark 2.5% and 97.5% percentile; outliers are not shown. The theoretical ratios are highlighted as dashed lines. Differences between the measured median FC values and theoretical values are indicated, among which the smallest ones are darkened. Results of the other sample pairs are shown in Supplementary Fig. 14. The DDA and TMT data were analyzed by PEAKS. Source data are provided as a Source Data file.

We firstly compared the performance of LFQ-DIA to LFQ-DDA and TMT. Several state-of-the-art software solutions (PEAKS, MaxQuant and FragPipe) were used for LFQ-DDA and TMT data analysis. Among them, PEAKS Studio^31^ quantified the most proteins for LFQ-DDA and provided the most accurate quantification for LFQ-DDA and TMT (Supplementary Notes 1 and 2, Supplementary Data 1 and 2, as well as Supplementary Figs. 2–9). LFQ-DIA data were analyzed in a spectral library-free manner, where the directDIA^32^ module in Spectronaut^33^ was used to search the DIA data against the protein sequence database of the 12 species directly (Supplementary Data 3, and Supplementary Figs. 10–13). As shown in Supplementary Figs. 10, 11, directDIA identified and quantified 10,986 ± 221 (mean ± standard deviation, sic passim) proteins and 52,733 ± 1542 peptides per run. From the 9 DIA runs, 11,988 proteins and 57,888 peptides were detected totally. Among them, 74% (8903) proteins and 69% (39,703) peptides were shared in all the runs, indicating lower missing values than those of LFQ-DDA (71% [8114/11,361] proteins and 58% [33,679/57,847] peptides shared in all the runs, Supplementary Figs. 2 and 3). Considering proteins and peptides shared in at least 2/3 replicates (runs for LFQ-DDA and LFQ-DIA, or reporter ion channels for TMT) in each sample group, directDIA detected 7% more (9831/9181) proteins and 14% more (46,505/40,805) peptides than LFQ-DDA, but 7% less (9831/10,572) proteins and 10% less (46,505/51,409) peptides than TMT (Fig. 2a). The highest number of proteins and peptides detected by TMT was possibly resulted from the prefractionation step in the TMT experiment. It should be noted that the protein inference algorithms may vary in different software tools and we kept only the leading protein in each protein group for simplicity, so the protein overlap can then be slightly underestimated.

Besides the numbers of quantified proteins and peptides, directDIA provided peptide-level quantification precision close to TMT. TMT resulted in smaller protein-level coefficient of variation (CV) values among three replicates in each sample group than directDIA, and LFQ-DDA showed the smallest CV values at peptide level among all the methods (Supplementary Figs. 4, 8, 12). However, it should be noted that the small CV values by LFQ-DDA were accompanied by the low numbers of quantified peptides and proteins.

We also calculated the fold change (FC) values of quantification results between each two of the three samples based on the average of the replicates of each sample (Fig. 2b and Supplementary Fig. 14). Only the proteins quantified in at least 2/3 replicates in each sample group and uniquely belonging to one species were taken into consideration. Among the 36 comparisons (by pairwise enumeration of the 3 samples as numerator and denominator) of the 12 species, LFQ-DDA yielded experimental median FC values closest to the theoretical values in 18 comparisons, while the experimental FC values showed high variability. The experimental median FC values by directDIA and TMT were closest to the theoretical values in 8 and 10 comparisons, respectively, indicating close quantitative accuracy of directDIA to TMT.

We then compared directDIA with alternative DIA data analysis methods based on experimental and predicted spectral libraries. For the experimental library-based method, DDA experiments were performed on the pool of all the samples with 12 HPRP-LC fractions. Spectronaut^33^ was used to build a spectral library from the DDA data and then analyzed the DIA data. For the predicted library-based method, DIA-NN^34^ was used to generate an in silico spectral library from the protein sequence database of the 12 species and then analyze the DIA data (Supplementary Data 3). Although these two methods detected more proteins and peptides totally, they resulted in much higher level of missing values than directDIA. For the DDA library-based method, only 52% [7168/13,917] proteins and 27% [23,058/84,573] peptides were shared in all the runs; and for DIA-NN, only 56% [7796/13,844] proteins and 36% [27,837/78,063] peptides were shared in all the runs, Supplementary Figs. 10 and 11. Considering proteins and peptides shared in at least 2/3 replicate runs in each sample group, directDIA detected 18% more (9831/8310) proteins and 54% more (46,505/30,204) peptides than the DDA library-based method, as well as 9% more (9831/9038) proteins and 33% more (46,505/34,899) peptides than DIA-NN (Supplementary Fig. 12). The three DIA methods resulted in similar quantification precision. In terms of deviation between experimental median FC values and the theoretical values, DIA-NN achieved the best quantitative accuracy, and directDIA outperformed the DDA library-based method (Supplementary Fig. 13).

With all the benchmarking results on the simulated microbial communities of 12 species, the superior performance of DIA approach with directDIA data analysis was demonstrated based on a comprehensive consideration of proteome coverage, as well as accuracy and precision in quantification. Overall, the median FC values measured by directDIA were close to the theoretical values, with the relative difference from the theoretical values, i.e., (median FC − theoretical value) / theoretical value, in the range of −40% to +71%, except for *Klebsiella aerogenes* (−79% to −46% relative difference) and *Klebsiella pneumoniae* (−64% to +235% relative difference). The possible reason for the lower quantitative accuracy of proteins from *K. aerogenes* and *K. pneumoniae* may be that the two species are from the same genus. Bacteria of the same genus are likely to share many identical or highly similar protein sequences, increasing the risk of misassignment of proteins at the species level.

Ribosomal proteins are highly conserved and high-abundant proteins that have been reported as favorable targets of metaproteomic analysis to derive the taxonomic composition of a microbial community^35^. Hence, we further tested whether ribosomal proteins are good quantification targets to accurately reflect the relative abundance of species (Supplementary Note 3 and Supplementary Data 3). Only the proteins uniquely belonging to one species were taken into consideration for fold change calculation. The quantitative accuracy of species based on ribosomal proteins was slightly better than that based on the total proteins (Supplementary Fig. 15). The more accurate median FC values and less dispersed distribution of measured FC values can be benefited from the relative high abundance of ribosomal proteins and the significantly smaller number of ribosomal proteins compared to all the proteins.

### Benchmarking on spike-in metaproteome samples

We further evaluated the performance of LFQ-DIA, LFQ-DDA, and TMT using real human gut microbial samples spiked with laboratory cultured species to mimic the difficulties encountered when analyzing complex microbiota samples. A human fecal sample was analyzed by metagenomic sequencing to identify the bacterial species composition of the sample. Six bacterial species not at a detectable abundance in the fecal sample were selected and cultured. For each species, the bacterial cell numbers were determined by optical density measurement and plate counting. The bacterial suspensions were mixed with three different cell number ratios to form three samples, wherein the low abundant species counted down to ~0.4% of the cell copies of the 6 species (as shown in Supplementary Table 4). Bacterial proteins were then extracted from the three mixtures. For each mixture, 1 μg of bacterial proteins were taken and spiked into 99 μg of microbial proteins extracted from the fecal sample to form the spiked samples. As a result, proteins of the 6 species accounted for 1% weight of the total proteins in the spiked samples.

**Fig. 3:**
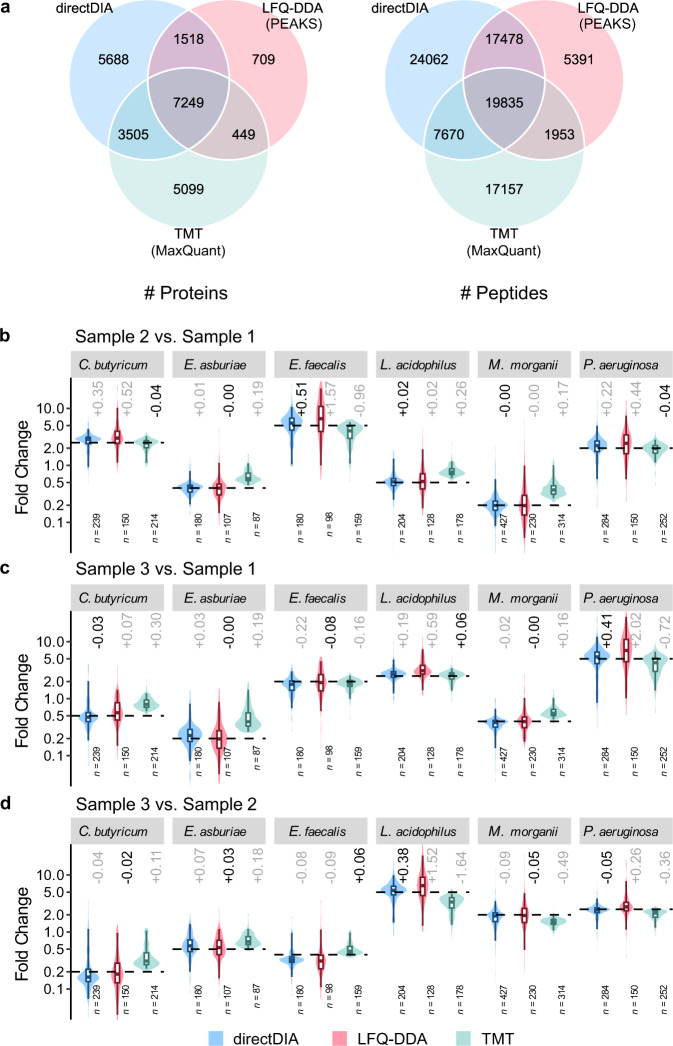
Performance evaluation of directDIA, LFQ-DDA, and TMT on the spike-in metaproteome samples with 6 species added to feces. **a** Overlap of proteins and peptides shared in at least 2/3 replicates in each sample group by different methods. **b** Measured fold change (FC) values of protein abundance between sample 2 (as numerator) and sample 1 (as denominator) for each species calculated based on the average of the replicates of each sample. **c** Measured FC values between sample 3 (as numerator) and sample 1 (as denominator). **d** Measured FC values between sample 3 (as numerator) and sample 2 (as denominator). Only proteins quantified in at least 2/3 replicates of each sample group and uniquely belonging to one species were taken into consideration. Numbers (*n*) of quantified proteins are indicated for each species. The boxes mark the first and third quantile and the lines inside the boxes mark the median; the whiskers mark 2.5% and 97.5% percentile; outliers are not shown. The theoretical ratios are highlighted as dashed lines. Differences between the measured median FC values and theoretical values are indicated, among which the smallest ones are darkened. The DDA data were analyzed by PEAKS, and the TMT data were analyzed by MaxQuant. Source data are provided as a Source Data file.

The LFQ-DIA, LFQ-DDA, and TMT data (Supplementary Table 5) were searched against a protein database combining proteomes of the 6 species and protein sequences from the metagenomic sequencing results of the fecal sample (491,768 entries in total). Among the several state-of-the-art software solutions, PEAKS Studio was chosen for LFQ-DDA and MaxQuant^36^ for TMT as they achieved the most accurate quantification on the dataset (Supplementary Note 4, Supplementary Data 4 and 5, as well as Supplementary Figs. 16–23). Similar with the results of the 12-species simulated microbial communities, directDIA outperformed LFQ-DDA on the spike-in metaproteome samples with superior proteome coverage and data completeness (Fig. 3a, Table 1, as well as Supplementary Figs. 24, 25). In average, directDIA quantified 18,268 ± 24 proteins and 70,272 ± 203 peptides per run. Considering those detected in at least 2/3 replicate runs in each sample group, directDIA quantified 17,960 proteins and 69,045 peptides, including 1514 proteins and 5899 peptides uniquely belonging to the spiked 6 species. In contrast, LFQ-DDA quantified 9925 proteins and 44,657 peptides, including 863 proteins and 2970 peptides from the spiked 6 species. In addition, directDIA yielded fewer missing values (91% [17,031/18,712] proteins and 88% [63,407/72,374] peptides shared in all the runs) compared with LFQ-DDA (84% [9077/10,774] proteins and 75% [38,542/51,384] peptides shared in all the runs). The comparison of performance between directDIA and LFQ-DDA on the 6 species spiked fecal samples is shown in Table 1.

**Table 1:** Performance comparison of directDIA and LFQ-DDA on the spike-in metaproteome samples.

	directDIA	LFQ-DDA (PEAKS)
Proteome coverage		
Quantified proteins per run^a^	18,268 ± 24	10,229 ± 13
Quantified peptides per run^a^	70,272 ± 203	47,137 ± 95
Reproducible proteins^b^	17,960	9925
Reproducible peptides^b^	69,045	44,657
Species-specific proteins^b,c^	1514	863
Species-specific peptides^b,c^	5899	2970
Data completeness		
Protein full/sparse ratio^d^	91% (17,031/18,712)	84% (9077/10,774)
Peptide full/sparse ratio^d^	88% (63,407/72,374)	75% (38,542/51,384)
Quantification precision		
Protein CV^e^	9.1% to 9.6%	9.3% to 9.7%
Peptide CV^e^	12.3% to 13.1%	8.4% to 8.8%
Quantification accuracy		
FC error^f^	−0.21 to +0.51	−0.09 to +2.02
FC relative error^g^	−20% to +15%	−22% to +40%
Outperforming frequency^h^	11	7

^a^mean ± standard deviation.

^b^shared in at least 2/3 replicate runs in each sample group.

^c^uniquely belonging to one of the 6 spiked species.

^d^ratio of proteins or peptides detected in all the runs (“full”) to those detected in at least one runs (“sparse”).

^e^coefficient of variation (CV) in median values within each sample group.

^f^median fold change (FC) − theoretical value.

^g^(median FC − theoretical value)/theoretical value.

^h^number of comparisons where the measured median FC values were closer to the theoretical values than the other quantification strategy.

From the detected proteins, those uniquely belonging to the 6 species were selected to evaluate the quantitative accuracy of the methods (Fig. 3b–d). Among the 18 FC comparisons of the 6 species, TMT achieved the most accurate measurement in only 4 comparisons. In the majority of the comparisons, TMT suffered from ratio compression that experimental median FC values deviated from theoretical values toward a 1:1 ratio (Supplementary Note 4). The bias came from superposition of reporter ion intensities of co-isolated and co-fragmented peptides. This phenomenon also resulted in the apparently low missing values. The spike-in metaproteome samples were much more complex than the 12-species samples. The proteins of the 6 species accounted for only 1% weight of the total proteins, and the background proteins from feces posed great challenges for accurate quantification of the 6-species proteins. Nevertheless, high quantitative accuracy of directDIA remained despite the sample complexity, where the median FC values were closest to the theoretical values in 7 of the 18 comparisons among the three quantification methods. The median FC values measured by directDIA were very close to the theoretical values, with the relative difference from the theoretical values, i.e., (median FC − theoretical value) / theoretical value, in the range of −20% to +15%, in contrast to −22% to +40% by LFQ-DDA. FC values measured by directDIA were also more accurate than LFQ-DDA in the majority of comparisons (11 out of 18) (Table 1). Notably, DIA-NN also showed remarkable quantitative performance (Supplementary Figs. 26 and 27, as well as Supplementary Data 6). However, spectral library prediction by DIA-NN from a huge protein sequence database (e.g., those used for clinical metaproteome samples hereafter) is hardly practical due to depletion of computing resources. Therefore, directDIA was still chosen as the most suitable method for large-scale quantitative metaproteomic analysis of real samples.

### Characterizing gut metaproteome in MCI by directDIA with metagenomics-based database

To demonstrate the utility of directDIA in characterizing clinical gut microbiome samples for biological research, we analyzed a cohort of fecal samples from 22 MCI patients and 34 controls (Fig. 1b, demographic data shown in Supplementary Table 6). Metagenomic sequencing was performed on a pool of all the samples to build a sample-specific protein sequence database (1,217,422 entries). Then, the DIA data were searched directly against the sample-specific database by directDIA, resulting in 70,925 proteins and 233,217 peptides quantified totally. The quantified peptides were annotated using Unipept^37^. Taxonomic abundances of gut microbiome from MCI patients and controls based on peptide quantities are shown in Supplementary Fig. 28 and discussed in Supplementary Note 5.

**Fig. 4:**
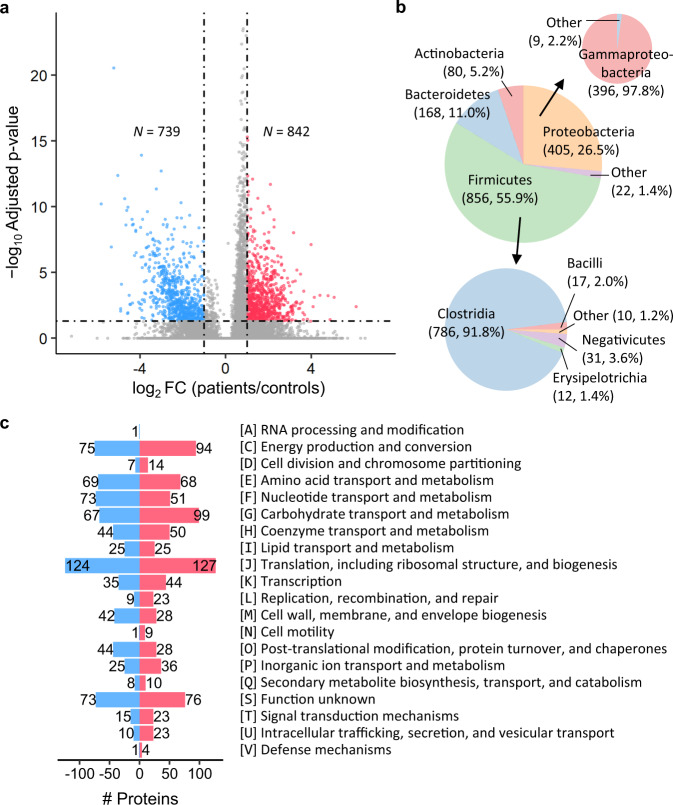
Differential gut microbial proteins between the MCI patients and controls. **a** Volcano plot indicating the differential proteins between the patients and controls. Proteins with fold change (FC, patients/controls) > 2 and p-value < 0.05 were colored red, while those with FC < 0.5 and *p*-value < 0.05 were colored blue. The p-values were given by MS1-MS2-combined statistical test in Spectronaut and adjusted by the Bonferroni method. **b** Distribution of taxonomy assigned to the differential proteins. **c** Numbers of the differential proteins in each category of clusters of orthologous groups (COG). Proteins more abundant in patients were colored red, while those more abundant in controls were colored blue.

Differential proteins between the MCI patients and controls were determined using abundance FC and statistical test by Spectronaut^38^. The Bonferroni method was conducted on the p-values given by the MS1-MS2-combined statistical test in Spectronaut for multiple testing correction to obtain a conservative result, and 1581 proteins with FC > 2 (or <0.5) and adjusted *p*-value < 0.05 were discovered (Fig. 4a). The FC cut-off values for differential proteins were determined by considering the quantification performance of directDIA on the simulated microbial community samples and the spike-in samples. Among the differential proteins, 1531 were annotated with taxonomy information by eggNOG^39^ (Supplementary Data 7). The classes Clostridia (51.3%) and Gammaproteobacteria (25.9%), as well as the phylum Bacteroidetes (11.0%) accounted for large proportions of the differential proteins (Fig. 4b). Functional characteristics of the differential proteins are presented in Fig. 4c. The differential proteins were annotated into 20 categories of clusters of orthologous groups (COG). For most of the COG categories, more differential proteins were found with high relative abundance in the MCI patients than in the controls. However, there were more differential proteins related to nucleotide transport and metabolism (category F), bacterial outer membrane component biogenesis (category M), as well as post-translational modification, protein turnover and chaperones (category O) that were less abundant in the MCI patients. We also observed that the stress response-related gut microbial chaperones, proteases and peroxidases were significantly changed in MCI (Supplementary Note 6 and Supplementary Fig. 29).

The differential proteins were further mapped to metabolic pathways in the Kyoto Encyclopedia of Genes and Genomes (KEGG) database^40^ using the KEGG orthology (KO) numbers annotated by eggNOG. For the dominant phyla and classes assigned to the differential proteins (Fig. 4b and Supplementary Fig. 28), KEGG pathway enrichment analysis was performed per taxon (Supplementary Figs. 30, 31, as well as Supplementary Data 8). Some enriched metabolic pathways, especially those representing basic biological functions, were shared among the selected taxa, extensively covering ribosome, amino acid biosynthesis, carbon metabolism, as well as glycolysis and gluconeogenesis. On the other hand, divergences were also observed among the altered metabolism of these taxa.

### Characterizing gut metaproteome of PC patients by directDIA with taxonomy-based databases

As an alternative to a metagenomics-based database, public proteome sequences taxonomically filtered based on 16S rRNA sequencing can also be utilized for metaproteomic analysis. We further analyzed a cohort of fecal samples with 15 PC patients and 15 controls (Fig. 1b, demographic data shown in Supplementary Table 7). In order to construct the protein sequence database for directDIA analysis, 16S rRNA gene sequencing was performed on the samples to identify the bacterial taxonomic composition. Accumulating the results of all the samples, 126 genera were identified (Supplementary Data 9), and the corresponding proteomes were downloaded from UniProt^41^. Since the taxonomy-based database was built at the general level, there can be many proteins in the database not included in the sample, which would waste a lot of calculation time and restrict the detection sensitivity. Meanwhile, there can also be proteins of the sample not included in the database, such as proteins from eukaryotes and food, due to the incompleteness of the UniProt database of bacteria. Therefore, we adopted a database refining strategy proposed in our previous study to optimize the protein sequence database^25^, illustrated in detail in Supplementary Fig. 32. For database refining, DDA experiments were performed on a pool of all the samples after prefractionation by HPRP-LC (12 fractions). De novo sequencing-assisted database searching by PEAKS was conducted on the DDA data against successively a database of stool microbial proteins from the Human Microbiome Project (HMP)^3^ (containing >4.8 million protein entries, including sequences from eukaryotes and food) and the database combining the proteomes from UniProt of the 126 genera (containing >17 million protein entries). The HMP stool database and the identified UniProt proteins (22,160 entries) were combined and used as the database for DIA analysis. The DIA data of individual samples were then analyzed by directDIA with the refined protein sequence database. Consequently, 66,196 proteins and 215,655 peptides were quantified totally. At the protein level, functional characteristics of differential proteins between the PC patients and controls are shown in Supplementary Fig. 33, Supplementary Data 10 and discussed in Supplementary Note 7. Enriched metabolic pathways are shown in Supplementary Figs. 34, 35, as well as Supplementary Data 11.

**Fig. 5:**
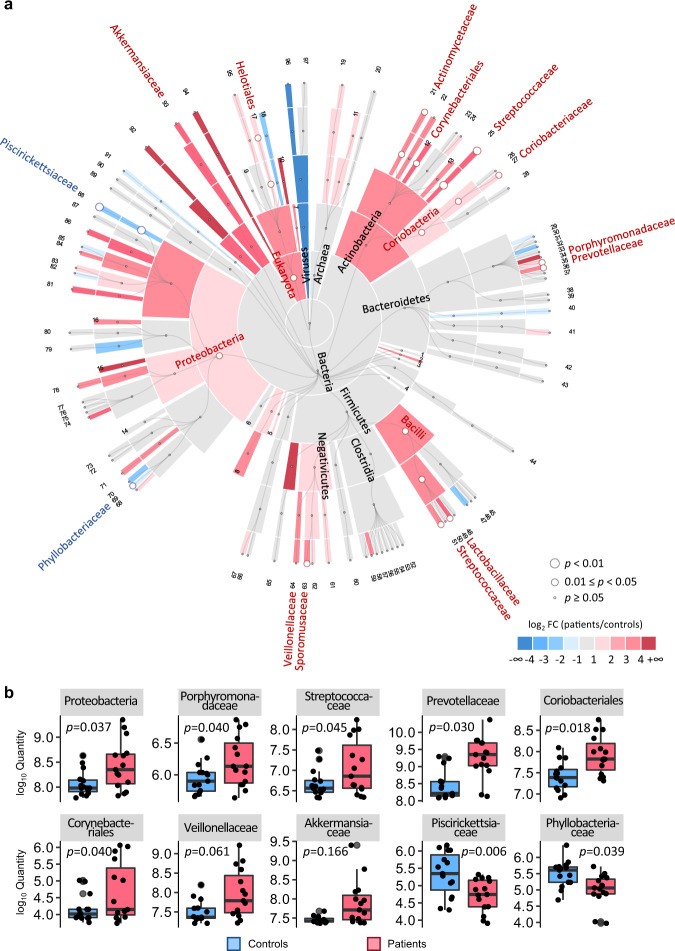
Taxonomic abundances based on microbial proteins of the PC patients and controls. **a** Cladogram illustrating abundance of taxa (domain to family). Colors indicate the log2 fold change (FC, patients/controls) between the patients and controls; circle sizes indicate the p-value (t-test). Names of the taxa are highlighted in red (more abundant in the patients) or blue (more abundant in the controls) color if their significant abundance differences between patients and controls were observed in this work (p-value < 0.05) or have been reported in previous studies. Information of the taxa with the labeled numbers are shown in Supplementary Data 12. **b** Boxplots showing the abundance of the differential taxa between the patients and controls. The boxes mark the first and third quantile and the lines inside the boxes mark the median; the whiskers extend from the ends of the inter-quartile range (IQR) to the furthest observations within the 1.5 times the IQR. Individual data points are overlaid as dots. The *p*-values are indicated. Source data are provided as a Source Data file.

Taxonomic information was assigned to the quantified peptides using Unipept^37^. Among the annotated peptides, 101,217 were matched to 79 families of microbes and 90,697 to 129 genera. We summed the quantitative information of all the quantified peptides at different taxonomic levels to demonstrate the proteome-based abundance of gut microbial taxa (Fig. 5, Supplementary Data 12, and Supplementary Note 8). We observed some significant abundance differences in taxa that have been reported in previous studies based on metagenomics or 16S rRNA gene sequencing^42–46^, including the phylum Proteobacteria, families Porphyromonadaceae, Streptococcaceae, and Prevotellaceae, as well as orders Coriobacteriales and Corynebacteriales, which showed higher abundance in the PC patients than in the controls. We found consistent results for the regulation of the families Veillonellaceae and Akkermansiaceae that have been observed more abundant in fecal samples of PC patients by 16S rRNA^47^, while the difference of their abundance was not statistically significant enough (p-value > 0.05 by t-test) in our results. We also observed changes in taxonomic abundance that have not been revealed by metagenomics and 16S rRNA, including the families Piscirickettsiaceae and Phyllobacteriaceae. It is reasonable that taxonomic abundances based on metagenomics and metaproteomics are different, probably due to divergences between genetic potential and functional activity^8^.

## Discussion

Metaproteomics is emerging as a powerful approach to perform large-scale characterization of proteins from microbiota, such as the human gut, linking microbial function to host disease pathogenesis^48^. Quantification strategies have been evaluated for proteomic analysis on the single organism or simple mixtures, but few studies have been performed to systematically compare their performance of quantification on complex metaproteomic samples. In this study, we demonstrated superior performance of directDIA based on a comprehensive consideration of proteome coverage, as well as accuracy and precision in quantification. With the feature of co-isolating peptide ions in parallel and recording all the fragment ions simultaneously, DIA can achieve more efficient ion usage than DDA^24^, resulting in surpassing proteome coverage. In addition, while LFQ-DDA uses only MS1-level elution profiles for quantification, DIA measures multiple fragments to alleviate interference of MS1 precursor profiles^23^, improving quantification accuracy. We also demonstrated that directDIA showed quantitative accuracy and precision close to TMT on the simulated microbial communities of 12 species, which is consistent with previous studies on single organism or simple mixture samples^17,49,50^. However, ratio compression limited TMT quantification accuracy for the spike-in metaproteome samples, and directDIA outperformed TMT in quantitative analysis of the spike-in metaproteome samples, even though MS3-based quantification was used in this study aiming at circumventing the issue of co-isolation of precursors. Notably, the MS3-based approach is restricted on specialized MS instruments, and the scanning speed limits the proteome coverage^51^. Furthermore, DIA has better scalability to large cohorts^49^, while the high accuracy of TMT will be negated due to unreliable correlation among multiple blocks if the sample size is larger than the number of multiplex channels^52^ (e.g., 30 and 56 clinical samples in this study).

In the past, a main drawback of DIA was that its data analysis required a spectral library, which is usually built from fractionated samples by DDA^21^. Library-free spectrum-centric methods performed worse to exploit highly comprehensive DIA data than library-based methods in terms of sensitivity^53^, since they rely on correct matching of MS1 precursor elution profiles with those of fragments^22^. With a recent improvement of the library-free workflow in Spectronaut, directDIA has been expected to perform on par with library-based DIA searches^54^. Our results corroborated this expectation and further demonstrated that directDIA can outperform library-based approaches on complex proteome samples of microbial communities. In this study, the spectral library was built by DDA with prefractionation. Signals of low-abundance peptides detected by extensive fractionation cannot be easily recovered by DIA processing tools with unfractionated DIA analyses^55^. These undetectable peptides can constitute a large proportion (denoted as *π*_0_) of false targets in the library, compromising the detection sensitivity from DIA data^19^. Indeed, it has been reported that libraries built by extensive fractionation are not beneficial to achieve good quantification performance by DIA^56^. Moreover, due to the high taxonomic heterogeneity of GM among individuals, for any specific samples, the library built from pooled samples with prefractionation would contain a large portion of undetectable peptides from other samples, resulting in an even larger *π*_0_ value, and hence further compromising the detection sensitivity from DIA data. Nevertheless, directDIA can build internal libraries using DIA data per se, which are highly specific to the samples, and thus is more suitable for profiling complex metaproteomic samples.

With reliable results from the spike-in metaproteome samples, directDIA was then applied to MCI and PC cohorts, demonstrating the utility to profile the taxonomic and functional characteristics of GM in clinical samples. We note that these results are only preliminary since limited sample sizes did not provide sufficient statistical power to draw strong conclusions. Nonetheless, our results showcased the power of the directDIA methodology for metaproteomics, which is scalable to large clinical cohort in pursuit of reliable biological findings. We present potential biological interpretations of the results in Supplementary Notes.

The only prior knowledge for directDIA data analysis is the protein sequence database. In this study, directDIA cooperated well with two types of databases, i.e., a database consisting of UniProt protein sequences taxonomically filtered based on 16S rRNA gene sequencing and a database built by whole-genome metagenomic sequencing, covering the current mainstream metaproteome protein sequence database construction methods. A previous study has systematically compared the two database construction approaches on metaproteomic analysis of GM, indicating that taxonomy-based UniProt databases can lead to poorer results than metagenomic databases when analyzing non-human GM but the gap is not severe when analyzing human GM samples^57^. The divergent numbers of sequences among different taxa deposited on UniProt could lead to biases of protein identification among taxa^57^, but not for relative quantitative comparison within each taxon across samples. In many cases in the absence of whole-genome metagenomic sequencing, taxonomy-based UniProt databases can be used as an alternative. In addition, public databases, e.g. HMP, can be used to combine with the UniProt database to compromise the biases of protein identification among taxa. However, we failed to search the PC dataset (30 runs) against the UniProt database of 126 genera (>17 million entries) by directDIA on our workstation (Intel Core i9-7960X CPU, 128 GB RAM). Thereby, we used the DDA data of the pooled and fractionated sample to refine the sequence database (but not for spectral library building), and the refined taxonomy-based UniProt database was used as complement to the HMP stool database for directDIA analysis. We expect that advances in software tools will make it practical to analyze large DIA cohorts against large comprehensive databases in the future so that DDA experiments are no longer needed.

In summary, our study demonstrated the superior performance of metaproteomics by directDIA based on a comprehensive consideration of proteome coverage in identification as well as accuracy and precision in quantification. The method has been successfully applied to human GM characterization, revealing the taxonomic and functional characteristics in PC and MCI. Notably, the results indicated that our workflow can cooperate well with both sample-specific database built by whole-genome metagenomics and public proteome sequences taxonomically filtered based on 16S rRNA sequencing. This directDIA approach will advance the metaproteomic applications to diverse samples, such as sludge, soil, and fermenting foods.

## Methods

### Bacteria culture and construction of simulated microbial communities

The twelve bacterial species (Supplementary Table 1) were purchased from American Type Culture Collection (ATCC) or China Center of Industrial Culture Collection (CICC). The bacterial cell numbers were determined by optical density measurement at the wavelength of 595 nm and plate counting. Growth curves were measured and all the species were collected in the stationary phase. We constructed three samples of simulated communities by mixing the twelve species at different cell numbers (Supplementary Table 2). The mixed cells were washed twice with phosphate-buffered saline (PBS) at pH 7.4 (Solarbio, Beijing, China) to remove the cultivation medium, and then stored at −80 °C.

### Preparation of the spike-in metaproteome samples

Similar with the 12-species simulated communities, we constructed another three samples by mixing 6 species at different cell numbers (Supplementary Table 4). After that, bacterial proteins were extracted from the 6-species mixtures. For each mixture, 1 μg of bacterial proteins were taken and then spiked into 99 μg of microbial proteins extracted from the fecal sample to form the spiked samples.

### Clinical sample collection

Thirty fecal samples from 15 PC patients before any clinical treatments and 15 non-PC volunteers were collected in Changhai Hospital (Shanghai, China). Fifty-six fecal samples from 22 MCI patients before any clinical treatment and 34 non-MCI volunteers were collected in at Shanghai Mental Health Center (Shanghai, China). Individuals taking antibiotics 2 weeks before sample collection were excluded. All participants only took ordinary Chinese diet during the 2 months before sample collection. Fecal specimens were collected in sterile collection tubes in the hospitals, sent to the laboratory within 2 h, and stored at −80 °C. All participants provided written informed consent to take part in the study. The study protocol was approved by the Ethics Committee of Shanghai Changhai Hospital, the Ethics Committee of Shanghai Mental Health Center, and the Ethics Committee of Fudan University, and complied with all relevant laws and regulations of China.

### 16S rRNA gene amplicon sequencing and data analysis

The total DNAs of fecal samples from PC patients and non-PC volunteers were extracted according to the protocol of QIAamp Mini DNA Kits (Product No. 51304, QIAGEN, Hilden, Germany). NanoDrop (Thermo Fisher Scientific, Waltham, MA, USA) was used to quantify DNA concentration and 1% agarose gel electrophoresis was used to assess DNA integrity. The 16S rRNA gene V3-V4 region that meets the DNA sample quality requirements (A260/280 = 1.8–2.0, total DNA > 500 ng) was selected to perform PCR amplification. After library construction using Truseq Kits (Illumina, Inc., San Diego, CA, USA), 2% agarose gel electrophoresis was used to select and purify library fragments. NanoDrop was used to assess quality, followed by sequencing using Illumina MiSeq PE250 high-throughput sequencer (Illumina, Inc., San Diego, CA, USA).

PANDAseq^58^ was used to splice paired-end reads according to the overlapping relationship to obtain long-reads in highly variable regions. Sharp’s internal procedures were used to process the spliced reads, involving the exclusion of quality reads below Q20, the elimination of N bases >3 reads and the remote control of reading between 250–500 nt to obtain clean reads. The clean reads with identical sequences were sorted by abundance. The operational taxonomic units (OTUs) for species classification were screened after clustering with Usearch^59^ (version 10.0.240) at a similarity of >97%. Finally, all clean reads were aligned to the OTU sequence^60^. One sequence from each OTU sequence was extracted as a representative of its classification. Ribosomal Database Project (http://dpc.me.ms.edu) was used for classification by comparing the extracted sequences with 165 known species. Based on the completed classification, the OTU abundance table was obtained by statistics according to the serial number of each representative OTU^61,62^.

### Whole-genome sequencing and assembly

The DNAs of fecal samples from MCI patients and non-MCI volunteers were extracted using HiPure Bacterial DNA Kits (Magen, Guangzhou, China) according to the manufacturer’s instructions. The DNA quality was detected using Qubit (Thermo Fisher Scientific, Waltham, MA, USA) and Nanodrop (Thermo Fisher Scientific, Waltham, MA, USA).

Qualified genomic DNA was first fragmented to a size of 350 bp by sonication. Both ends were flattened with enzymes, followed by the addition of an A-base to each end. The adapter was connected with a specific ligase. A library of DNA mixtures was obtained based on the protocol of NEBNext ΜLtra DNA Library Prep Kits for Illumina (NEB, Ipswich, MA, USA). DNA fragments with lengths of 300–400 bp were enriched by PCR. The enriched products were purified by AMPure XP system (Beckman Coulter, Brea, CA, USA). The size distribution of libraries was analyzed using a 2100 Bioanalyzer (Agilent, Santa Clara, CA, USA), followed by quantitative analysis using real-time PCR. Genome sequencing was performed on an Illumina Novaseq 6000 sequencer (Illumina, Inc., San Diego, CA, USA) using pair-end technology.

Raw data from the Illumina platform were filtered using FASTP^63^ (version 0.18.0) according to the following criteria: (1) removing reads with ≥10% unidentified nucleotides; (2) removing reads with ≥50% bases with Phred quality scores ≤ 20; (3) removing reads aligned to the barcode adapter. After filtering, clean reads retained were used for genome assembly. The clean reads of each sample were assembled using SOAPnuke^64^ (version 1.5.2), and the sequencing reads were mapped to the host genome using Bowtie2^65^ (version 2.3.5). The host contamination reads were trimmed to obtain high-quality clean sequencing reads.

### Sample preparation for metaproteomic analysis

For the simulated microbial community samples, 0.5 mL of lysis buffer containing 1% sodium dodecyl sulfate (SDS), 8 M urea, 20 mM Tris-HCl (pH = 8.8) and protease inhibitor cocktail (EDTA-free, 1×) was added into the sterile collection tubes containing the samples, and then the tubes were transferred into an ice bath. The ice bath was settled inside a SCIENTZ-II D ultrasonic crusher (Scientz, Ningbo, China) to homogenize the cells (50 W, 20 Hz, 10 min). Cell debris were removed by centrifugation (13,500 *g*, 10 min, 4 °C). The protein concentration of the supernatant was measured by the Pierce BCA assay Kit (Thermo Fisher Scientific, Waltham, MA, USA), and then lyophilized to obtain the dried protein powders for subsequent usage.

Gut microbial cells were enriched from fecal samples by differential centrifugation^25,66^. Briefly, 20 mL PBS was added to 0.5 g fecal sample, mixed for 30 min by a shaker under room temperature and 100 rpm, and centrifuged (500 *g*, 5 min, 4 °C) to remove the precipitates. Then, the supernatant was centrifuged at 12,000 *g* for 10 min to collect precipitates. The precipitates from the low-speed centrifugation (500 *g*) were collected and subjected to the differential centrifugation procedure again. And the two final precipitates from the high-speed centrifugation (12,000 *g*) were combined for subsequent processing. The collected precipitates were milled under liquid nitrogen. Then, 0.5 mL lysis solution containing 2% SDS, 100 mM dithiothreitol (DTT) and 20 mM Tris-HCl (pH = 8.8) was added into the milled powder, heated at 95 °C for 30 min, and the precipitates were removed through centrifugation (12,000 *g*, 10 min, 4 °C) to collect supernatants. Afterwards, pre-cooled acetone solution at −20 °C of five-fold volume of the lysis solution was added into the tube, incubated at −20 °C for 4 h. The supernatant was removed through centrifugation, and the precipitates were washed twice with 90% pre-cooled acetone. Finally, the precipitates were dried at room temperature, and 0.5 mL lysis buffer was added to dissolve the precipitates. The protein concentration was measured by the Pierce BCA assay Kit (Thermo Fisher Scientific, Waltham, MA, USA), and then lyophilized to obtain the dried protein powders for subsequent usage.

For proteolysis, 300 μg of protein was dissolved in 300 μL 8 M urea. Then, 6.1 μL of 0.5 M Tris-(2-carboxyethyl) phosphine (TCEP) was added and incubated at 37 °C and 600 rpm for 1 h. After that, 18 μL of 0.5 M iodoacetamide (IAA) solution was added and incubated at 25 °C in the darkness for 45 min. Next, 1.5 mL of pre-cooled acetone at −20 °C was added and incubated at −20 °C for 4 h, followed with centrifugation and washing by pre-cooled acetone twice, and finally dried at room temperature. Then, 200 μL of 0.1 M triethylammonium bicarbonate (TEAB) solution was added to dissolve the dried proteins. 6 μg of trypsin (Hualishi Technology, Beijing, China) was added into the dissolved proteins and incubated under 37 °C and 600 rpm for 16 h. The peptides were desalted by MonoSpin C18 column (GL Sciences, Tokyo, Japan) and quantified by Pierce quantitative colorimetric peptide assay (Thermo Fisher Scientific, Waltham, MA, USA).

### TMT labeling

The 12-species samples and spike-in metaproteome samples were labeled with the 10-plex TMT kit (Thermo Fisher Scientific, Waltham, MA, USA) according to the manufacturer’s instructions. For each TMT label reagent, 41 µL of anhydrous acetonitrile was added, and then the acetonitrile-dissolved reagent was added to 100 μg of peptides. After reaction at room temperature for 1 h, 8 µL of 5% hydroxylamine was added to each reagent tube, followed with reaction at room temperature for another 45 min. After desalting, the labeling efficiency was determined by a 2 h DDA run using LC-MS/MS. With the labeling efficiency greater than 99%, the labeled samples were mixed and desalted by Pierce C18 spin column (Shimadzu, Tokyo, Japan).

### HPRP-LC peptide separation

The mixed TMT labeled sample (450 μg) was dissolved in 450 μL solvent A (5 mM ammonium acetate in 5% acetonitrile) and fractionated by HPRP-LC on a Dionex Ultimate 3000 LC system (Thermo Fisher Scientific, Waltham, MA, USA) using an XBridge Peptide BEH C18 column (130 Å, 3.5 µm, 2.1 mm × 100 mm, Waters Corporation, Milford, MA, USA). A non-linear gradient of 75 min was used (Supplementary Table 8) for LC separation. Phase A was 5 mM ammonium acetate in 5% acetonitrile (pH = 10), and phase B was 5 mM ammonium acetate in 80% acetonitrile (pH = 10). Fractions were collected from 5 min to 65 min with the flow rate of 500 μL/min. For the 12-species samples, each fraction was collected every 45 s. Totally 80 fractions were collected and combined into 16 final fractions. For the spike-in metaproteome samples, each fraction was collected every 60 s. Totally 60 fractions were collected and combined into 12 final fractions.

For spectral library building, 150 µg unlabeled peptides from each sample of the 12-species simulated communities were mixed into a pool (for a total of 450 µg). Also, 20 µg peptides from each sample of the PC patients and non-PC volunteers were mixed into a pool (for a total of 600 µg). The final concentration of each pooled sample was 1 μg/μL. Each of the two pooled samples was fractionated by HPRP-LC into 60 fractions (each fraction collected every 60 s) and combined into 12 final fractions. The HPRP-LC fractionation system was same as the one for the mixed TMT labeled sample fractionation.

### LC-MS/MS analysis

For each sample, 10 μg of peptides were dried by vacuum centrifugation and resuspended in 30 μL 0.1% formic acid in water spiked with iRT peptides (Biognosys, Schlieren, Switzerland, 1x). The samples were analyzed by a nanospray Orbitrap Fusion Lumos Tribrid mass spectrometer (Thermo Fisher Scientific, Waltham, MA, USA) with a nano UPLC system (Waters Corporation, Milford, MA, USA). For each injection, 1 µg of peptides were loaded to a C18 column (75 μm × 25 cm, Waters Corporation, Milford, MA, USA) and separated with a 130 min gradient (Supplementary Table 8). Phase A was 0.1% formic acid in water, and phase B was 0.1% formic acid in 80% acetonitrile. The LC flow rate was 250 nL/min, and the column temperature was 40 °C.

The unlabeled 12-species samples and spike-in metaproteome samples with and without prefractionation, the TMT labeled samples with prefractionation, and the pooled PC sample with prefractionation were analyzed in DDA mode. For TMT, MS parameters were set as follows: (1) MS1: scan range (*m*/*z*) = 350–1500; resolution = 120,000; AGC target = 8e5; maximum injection time = 50 ms; number of charges = 2–5; dynamic rejection time = 30 s; (2) HCD-MS2: detector = ion trap; resolution = 30,000; isolation window = 1; AGC target = 10,000; maximum injection time = 120 ms; collision energy = 35%; (3) HCD-MS3: precursor selection range = 400–2000; precursor ion exclusion, low = 18, high = 5; isolation window = 2; collision energy = 65%; resolution = 50,000; AGC target = 50,000; maximum injection time = 86 ms. For DDA, the MS1 parameters were the same as TMT, and HCD-MS2 parameters were set as follows: resolution = 15,000; isolation window = 4; AGC target = 50,000; maximum injection time = 25 ms; collision energy = 30%.

The unlabeled 12-species samples and spike-in metaproteome samples without prefractionation, as well as the individual clinical samples were analyzed in DIA mode with 50 variable isolation windows (Supplementary Table 9). HCD-MS2 parameters were set as follows: resolution = 30,000; AGC target = 1e5; collision energy = 33%; maximum injection time = 54 ms. Other parameters were the same as DDA.

### LFQ-DDA data analysis of the 12-species samples and spike-in metaproteome samples

Raw DDA data of the unlabeled 12-species samples were analyzed by search against a database combining the sequences of the 12 species downloading from UniProt Proteomes (https://www.uniprot.org/, accessed in June 2020): *Clostridium butyricum* (4245 entries), *Escherichia coli* (5062 entries), *Enterococcus casseliflavus* (3112 entries), *Klebsiella aerogenes* strain ATCC 13048 (4909 entries), *Lactobacillus acidophilus* (1859 entries), *Bacteroides fragilis* strain ATCC 25285 (4234 entries), *Citrobacter freundii* (5149 entries), *Enterobacter asburiae* (5254 entries), *Pseudomonas aeruginosa* (5564 entries), *Klebsiella pneumoniae* (5126 entries), *Enterococcus faecalis* (3240 entries), and *Morganella morganii* (3510 entries). Data of the spike-in metaproteome samples were searched against a database combining the UniProt sequences of the 6 species and proteins translated from the metagenomic sequencing data of the fecal sample (468,096 entries). All of the software workflows were run using the default settings with modifications to make their results comparable. Trypsin was set as enzyme, and the maximum number of missed cleavages was set as 2. Carbamidomethylation (C) was specified as a fixed modification. Oxidation (M) and Acetylation (Protein N-term) were specified as variable modifications. Protein quantification is performed using unique and razor peptides as default settings of the software.

1. PEAKS workflow: PEAKS Studio^31^ (version X+, Bioinformatics Solutions Inc., Waterloo, Canada) was used. The MS1 tolerance was set as 7 ppm, and the MS2 tolerance was 0.02 Da. The false discovery rate (FDR) cut-off at both peptide and protein level was 1% by using a target-decoy strategy. Other parameters were default. In order to export the complete quantification results, protein significance filter was set to 0, protein fold change filter to 1 and unique peptide filter to 1 in the export settings.
2. MaxQuant workflow: MaxQuant^36^ (version 2.0.3.0) was used. MaxLFQ was on. Peptide-spectrum match (PSM) and protein FDR cut-offs were 1%. Match between runs was on. Other parameters were default.
3. FragPipe workflow: FragPipe (version 17.1) with MSFragger^67^ (version 3.4), Philosopher^68^ (version 4.1.0), and IonQuant^69^ (version 1.7.17) was used. The built-in workflow for LFQ with match between runs was selected. Peptide and protein FDR cut-offs were 1%. Other parameters were default.

### TMT data analysis of the 12-species samples and spike-in metaproteome samples

Raw TMT MS data were analyzed by PEAKS Studio, MaxQuant, and FragPipe. For PEAKS Studio, TMT 10-plex and MS3 quantification were selected. MS2 tolerance was 0.3 Da. Deamidation (NQ) and Oxidation (M) were specified as variable modifications. For MaxQuant, Reporter ion MS3 and TMT 10-plex were selected. For FragPipe, the built-in TMT10-MS3 workflow was selected. Other parameters were the same as LFQ-DDA.

### DIA data analysis of the 12-species samples and spike-in metaproteome samples

Raw DIA data of the 12-species samples were analyzed by directDIA, the DDA library-based method, and predicted library-based method. Raw DIA data of the spike-in metaproteome samples were analyzed by directDIA and DIA-NN.

1. directDIA workflow: Spectronaut^33^ (version 16.2.220903, Biognosys AG, Schlieren, Switzerland) was used. The raw DIA data were searched against the protein sequence database directly. The directDIA workflow is a two-step process: first, MS2 information are extracted from DIA data and searched against the sequence database like DDA to generate an internal spectral library; next, the targeted analysis of DIA data is performed using the internal spectral library. Q-value cut-off at both precursor and protein level was set as 1%. Other parameters were default.
2. The DDA library-based workflow: Spectronaut was used to build a spectral library by searching the raw DDA data of the pooled, fractionated 12-species sample against the protein sequence database with default settings. The spectral library was then used to analyze the raw DIA data using the same parameters as directDIA.
3. The predicted library-based workflow: DIA-NN^34^ (version 18.0) was used for library-free analysis. Deep learning-based in silico spectral library generation was enabled. To reduce computation burden, the maximum number of missed cleavages was 1 as default. Protein inference was performed based on protein names. For benchmarking purposes, heuristic protein inference was enabled to make sure that no protein was present simultaneously in multiple protein groups. Other parameters were default.

### Database refining for DIA analysis of the PC samples

Raw DDA data of the pooled and fractionated PC samples were analyzed by PEAKS Studio using a database refining strategy^25^. All the DDA MS/MS spectra were analyzed by de novo sequencing and searched against the database of stool microbial proteomes downloaded from HMP (4,854,034 entries, https://hmpdacc.org/, accessed in November 2017). Database searching parameters were the same as LFQ-DDA data analysis. Those identified by database searching with PSM-level FDR < 1% were assigned to a peptide from the HMP database. Other DDA MS/MS spectra with de novo sequencing average local confidence (ALC) > 80% were then searched against a database combining the proteomes from UniProt of the 126 genera identified by 16S rRNA gene sequencing (17,465,047 entries). Those identified with PSM-level FDR < 1% were assigned to a peptide from the UniProt database. The remaining spectra with de novo sequencing ALC > 80% were reported as de novo only results. Part of the de novo only results were assigned to proteins from the HMP or UniProt database using the SPIDER^70^ algorithm in PEAKS. The HMP stool database and the identified UniProt proteins (22,160 entries) were combined and used as the refined protein sequence database for library-free DIA analysis.

### DIA data analysis of the PC and MCI samples

Raw DIA data were analyzed by Spectronaut^33^ (version 15.4.210913). The PC data were analyzed by directDIA against the refined database containing HMP and the filtered UniProt proteomes. The MCI data were analyzed by directDIA against a database translated from the metagenomic sequencing data (1,217,422 entries). Global imputing was selected that missing values were imputed based on a random sampling from a distribution of low abundant signals taken across the entire experiment. Other parameters were default.

### Bioinformatic analysis of the PC and MCI samples

Protein inference was performed by the software for protein identification and quantification. Only the leading protein (with the strongest evidence and ranked first in the result) in each protein group was taken into consideration in all the subsequent analysis.

Differential proteins were determined by FC and statistical test by Spectronaut, where the p-values were calculated using a model combining MS1 and MS2 quantification^38^. FC values and raw p-values of all the quantified proteins were then exported to spreadsheets. The Bonferroni method was conducted for multiple testing correction of the p-values to obtain a conservative result. Proteins with FC > 2 (or <0.5) and adjusted p-value < 0.05 were considered as differential proteins. Annotation of the differential proteins was performed using eggNOG^39^ (version 5.0, http://eggnogdb.embl.de/, accessed in November 2021). COG and KO annotations, as well as taxonomic information were extracted from the eggNOG results. For each of the dominant taxa assigned to the differential proteins, KEGG enrichment analysis was perform using the R package “clusterProfiler”^71^ (version 4.2.0). The KO numbers matched with the differential proteins were used as the gene list of interest. The KEGG entries of all the organisms that belong to the taxon and are available in the KEGG database^40^ (https://www.genome.jp/kegg/, accessed in November 2021) were used as the background genes. The p-values by hypergeometric test were adjusted using the Benjamini-Hochberg method.

The quantified peptides were subjected to Unipept^37^ (version 4.3, https://unipept.ugent.be/, accessed in November 2021) for taxonomic analysis using the lowest common ancestor approach. Leucine and isoleucine were considered equal. Peptides matched to Metazoa and Viridiplantae (probably from human or food) were excluded. If a taxon had only one peptide, it was removed and the peptide was assigned to the parental taxon. Abundance of each taxon was determined by summing the quantities of all peptides corresponding to the taxon.

The taxonomic abundance analysis was performed using Python (version 3.5.6, Anaconda distribution version 4.2.0, https://www.anaconda.com/). Other statistics was conducted using R (version 3.5.1 and 4.0.2, Microsoft R Open distribution, https://mran.microsoft.com/open). AntV G2 (version 3.2.7, https://g2.antv.vision/) was used to plot the cladograms illustrating abundance of taxa. The R packages “ggplot2” (version 3.0.0) and “VennDiagram” (version 1.6.20) were used for other data visualization.

### Reporting summary

Further information on research design is available in the Nature Research Reporting Summary linked to this article.

## Supplementary information

Supplementary Material

Reporting Summary

Data Set 1

Data Set 2

Data Set 3

Data Set 4

Data Set 5

Data Set 6

Data Set 7

Data Set 8

Data Set 9

Data Set 10

Data Set 11

Data Set 12

## Data Availability

All raw MS data, spectral libraries and search results generated in this study have been deposited to the ProteomeXchange via the iProX^72^ partner repository with accession numbers PXD031301 or IPX0003851000. Public proteome databases used in this study are available at UniProt (https://www.uniprot.org/) and HMP (https://hmpdacc.org/), and custom databases have been deposited to the ProteomeXchange/iProX repository. The source data underlying all figures including statistics are provided as a Source Data file.

## Source data

source data

## Code availability

Custom scripts for post-analysis data processing and visualization in this paper are available at Github (https://github.com/lmsac/metaproteomics-utilities).
