# Supplementary material for: Data-independent acquisition boosts quantitative metaproteomics for deep characterization of gut microbiota
Source: NPJ Biofilms Microbiomes. 2023 Jan 24;9:4. doi: 10.1038/s41522-023-00373-9 (PMC9873935; doi:10.1038/s41522-023-00373-9)
Supplement: Supplementary file 1 — Supplementary Material [file 41522_2023_373_MOESM1_ESM.pdf]

Supplementary information for

Data-independent acquisition boosts quantitative metaproteomics for deep characterization of gut microbiota

Jinzhi Zhao et al.

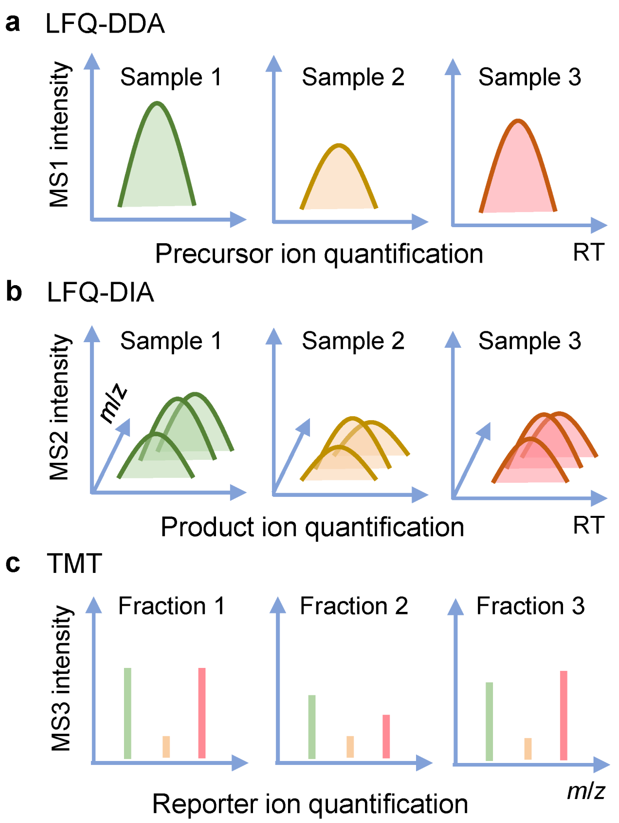

Supplementary Figure 1. Schematic illustration of the LFQ-DDA, LFQ-DIA and TMT quantification strategies used in this study.

(**a**) In LFQ-DDA experiments, quantification is performed based on MS1-level precursor chromatogram profiles for each sample. (**b**) In LFQ-DIA experiments, quantification is performed based on MS2-level fragment chromatogram profiles for each sample. (**c**) In TMT experiments, samples are multiplex labeled, mixed, and then prefractionated by HPRP-LC. Quantification is performed based on MS3-level reporter ion peak intensities.

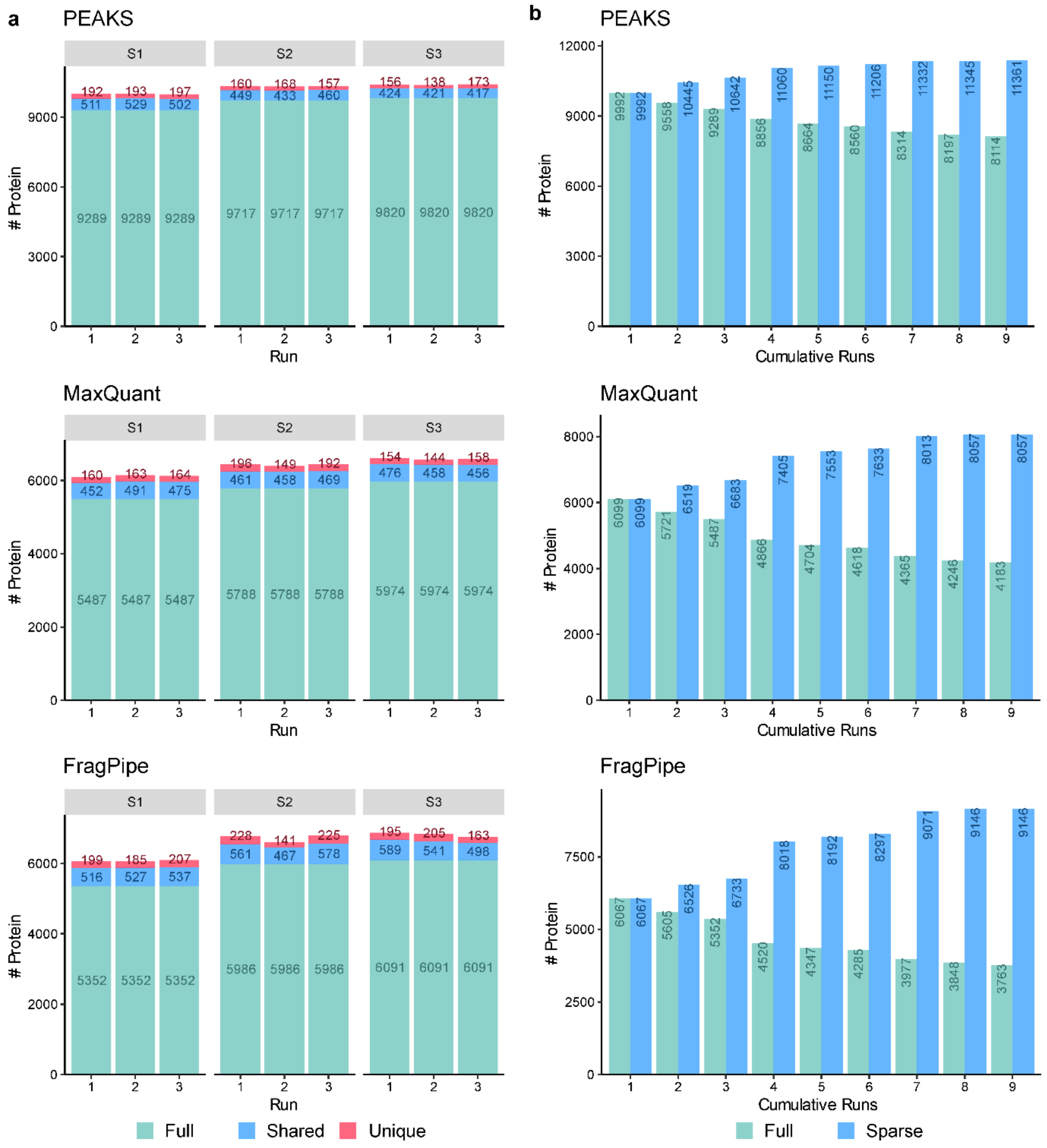

Supplementary Figure 2. Proteins quantified by different software solutions from LFQ-DDA data of the simulated microbial communities of 12 species.

(**a**) Numbers of quantified proteins per run. “Full” represents proteins quantified in all the runs of a sample; “shared” represents proteins quantified in 2 runs of a sample; “unique” represents proteins quantified in only 1 run. (**b**) Numbers of cumulative proteins from run 1 to 9 (including sample 1, sample 2 and sample 3). “Full” represents proteins shared in the cumulative runs; “sparse” represents proteins quantified in at least 1 run in the cumulative runs.

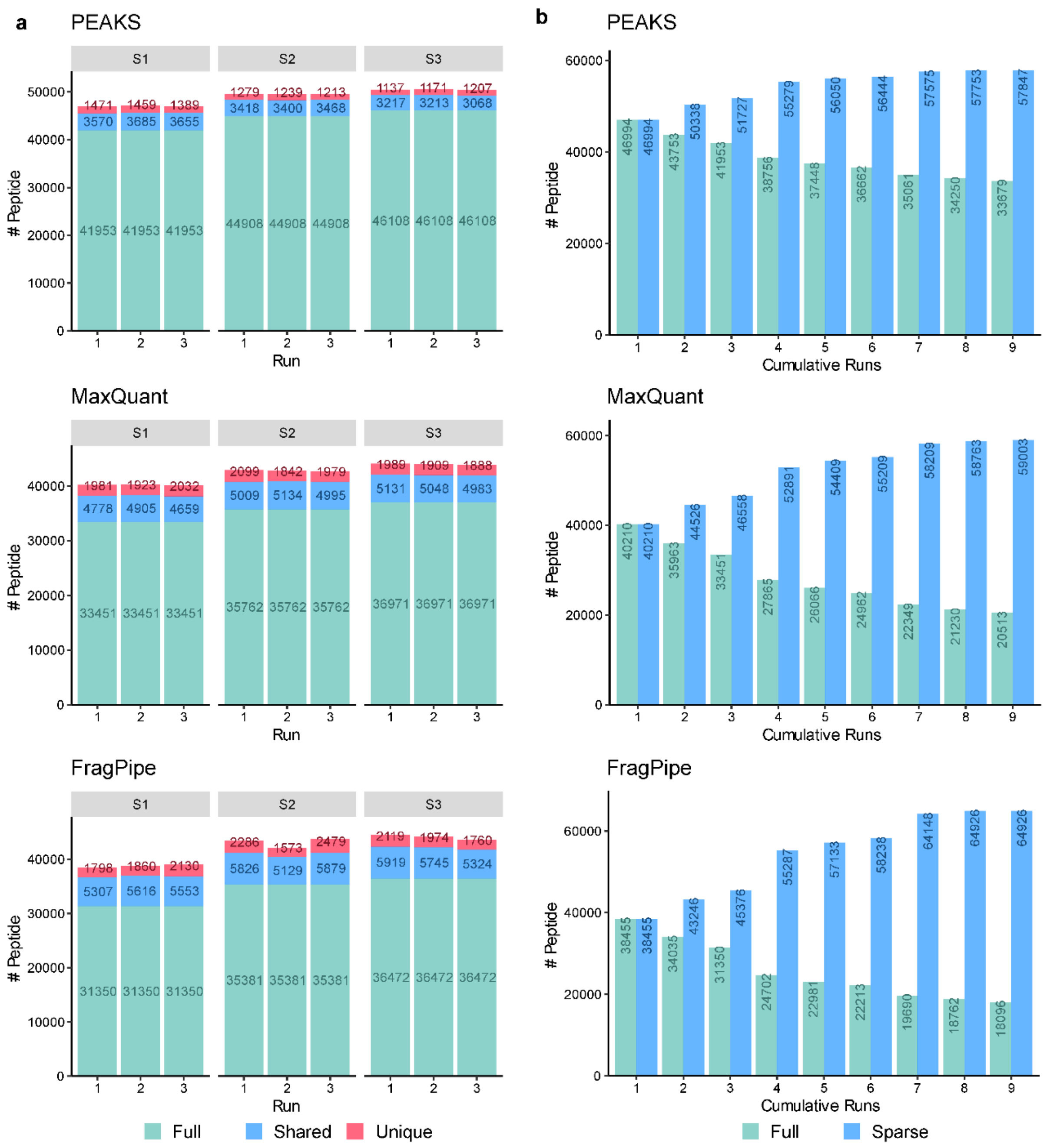

Supplementary Figure 3. Peptides quantified by different software solutions from LFQ-DDA data of the simulated microbial communities of 12 species.

(**a**) Numbers of quantified peptides per run. “Full” represents peptides quantified in all the runs of a sample; “shared” represents peptides quantified in 2 runs of a sample; “unique” represents peptides quantified in only 1 run. (**b**) Numbers of cumulative peptides from run 1 to 9 (including sample 1, sample 2 and sample 3). “Full” represents peptides shared in the cumulative runs; “sparse” represents peptides quantified in at least 1 run in the cumulative runs.

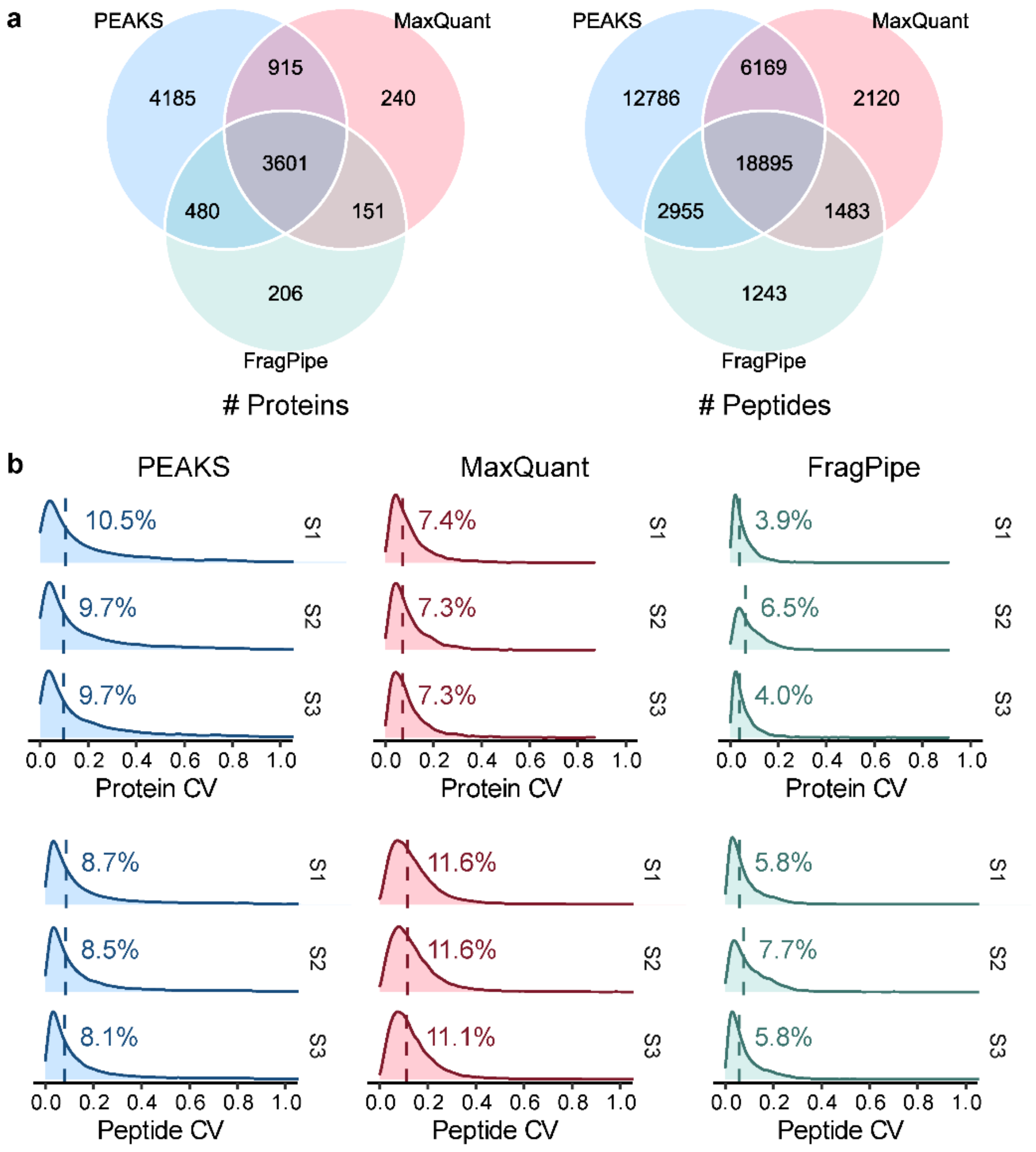

Supplementary Figure 4. Performance comparison among different software solutions for LFQ-DDA data analysis on the simulated microbial communities of 12 species.

(**a**) Overlap of proteins and peptides shared in at least 2/3 replicate runs in each sample group by different software solutions. (**b**) Coefficient of variation (CV) values of protein and peptide quantification results. For each sample, only proteins and peptides quantified in all the three replicates were taken into consideration for CV calculation. The medians are indicated. Source data are provided as a Source Data file.

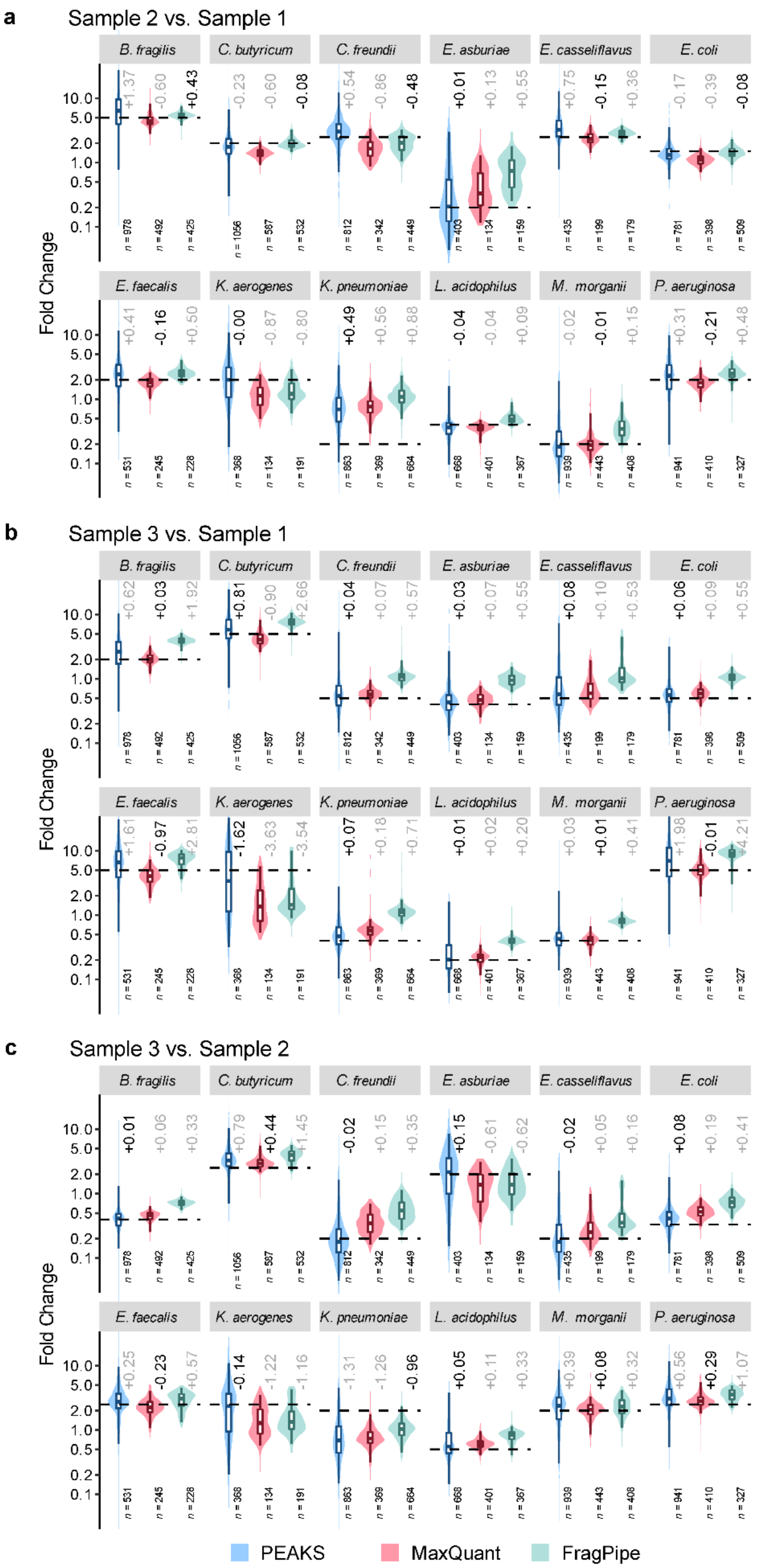
a
Sample 2 vs. Sample 1
b
Sample 3 vs. Sample 1
c
Sample 3 vs. Sample 2
B. fragilis
C. butyricum
C. freundii
E. asburiae
E. casseliflavus
E. coli
E. faecalis
K. aerogenes
K. pneumoniae
L. acidophilus
M. morganii
P. aeruginosa
Fold Change
PEAKS
MaxQuant
FragPipe

Supplementary Figure 5. Comparison of quantification accuracy among different software solutions for LFQ-DDA data analysis on the simulated microbial communities of 12 species. (**a**) Measured fold change (FC) values of protein abundance for each species between sample 2 (as numerator) and sample 1 (as denominator). (**b**) Measured FC values between sample 3 (as numerator) and sample 1 (as denominator). (**c**) Measured FC values between sample 3 (as numerator) and sample 2 (as denominator). FC values were calculated based on the average of the replicates of each sample. Only proteins quantified in at least 2/3 replicates of each sample group and uniquely belonging to one species were taken into consideration. Numbers (n) of quantified proteins are indicated for each species. The boxes mark the first and third quantile and the lines inside the boxes mark the median; the whiskers mark 2.5% and 97.5% percentile; outliers are not shown. The theoretical ratios are highlighted as dashed lines. Differences between the measured median FC values and theoretical values are indicated, among which the smallest ones are darkened. Source data are provided as a Source Data file.

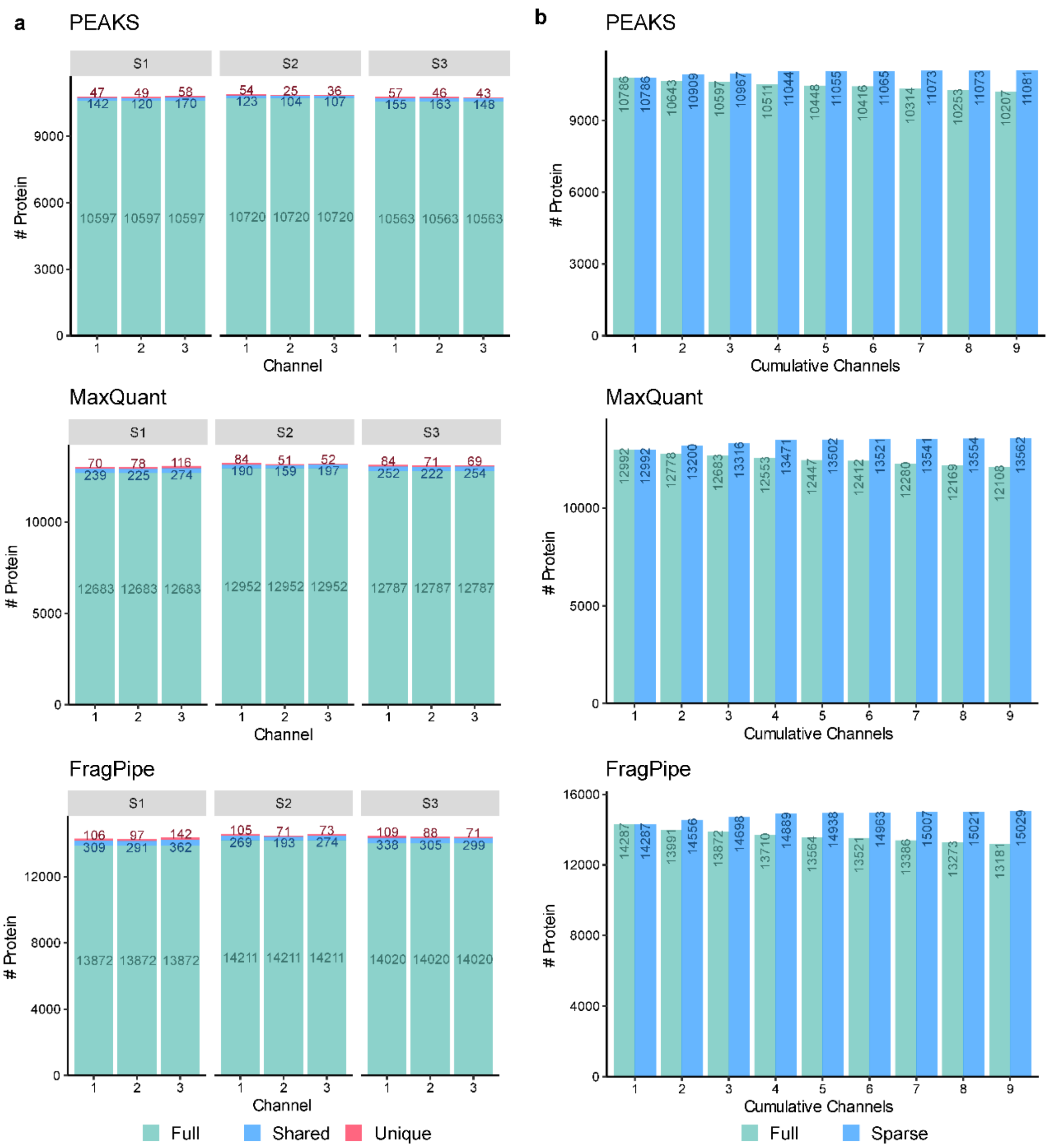

Supplementary Figure 6. Proteins quantified by different software solutions from TMT data of the simulated microbial communities of 12 species.

(**a**) Numbers of quantified proteins per channel. "Full" represents proteins quantified in all the channels of a sample; "shared" represents proteins quantified in 2 channels of a sample; "unique" represents proteins quantified in only 1 channel. (**b**) Numbers of cumulative proteins from channel 1 to 9 (including sample 1, sample 2 and sample 3). "Full" represents proteins shared in the cumulative channels; "sparse" represents proteins quantified in at least 1 channel in the cumulative channels.

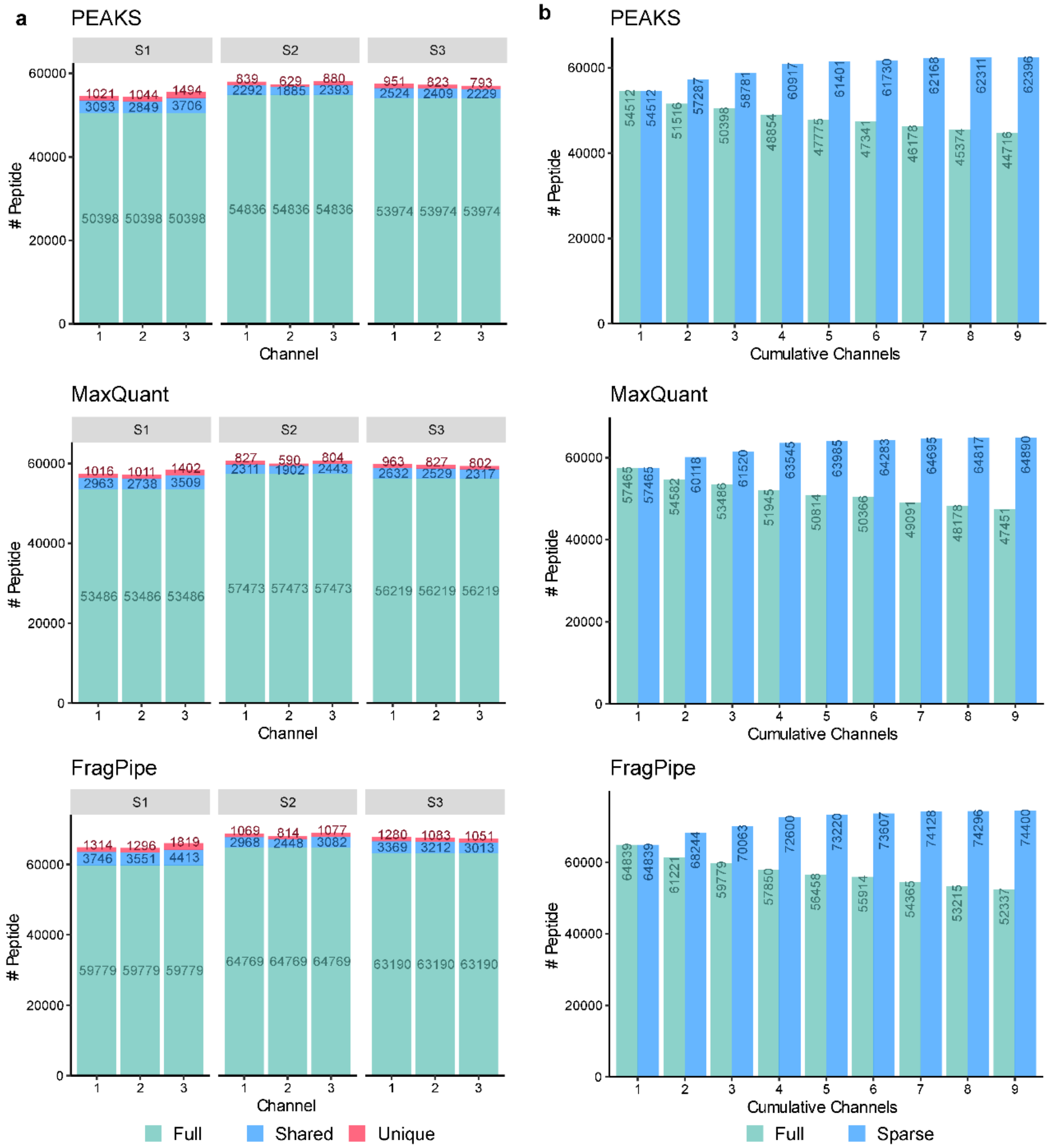

Supplementary Figure 7. Peptides quantified by different software solutions from TMT data of the simulated microbial communities of 12 species.

(**a**) Numbers of quantified peptides per channel. “Full” represents peptides quantified in all the channels of a sample; “shared” represents peptides quantified in 2 channels of a sample; “unique” represents peptides quantified in only 1 channel. (**b**) Numbers of cumulative peptides from channel 1 to 9 (including sample 1, sample 2 and sample 3). “Full” represents peptides shared in the cumulative channels; “sparse” represents peptides quantified in at least 1 channel in the cumulative channels.

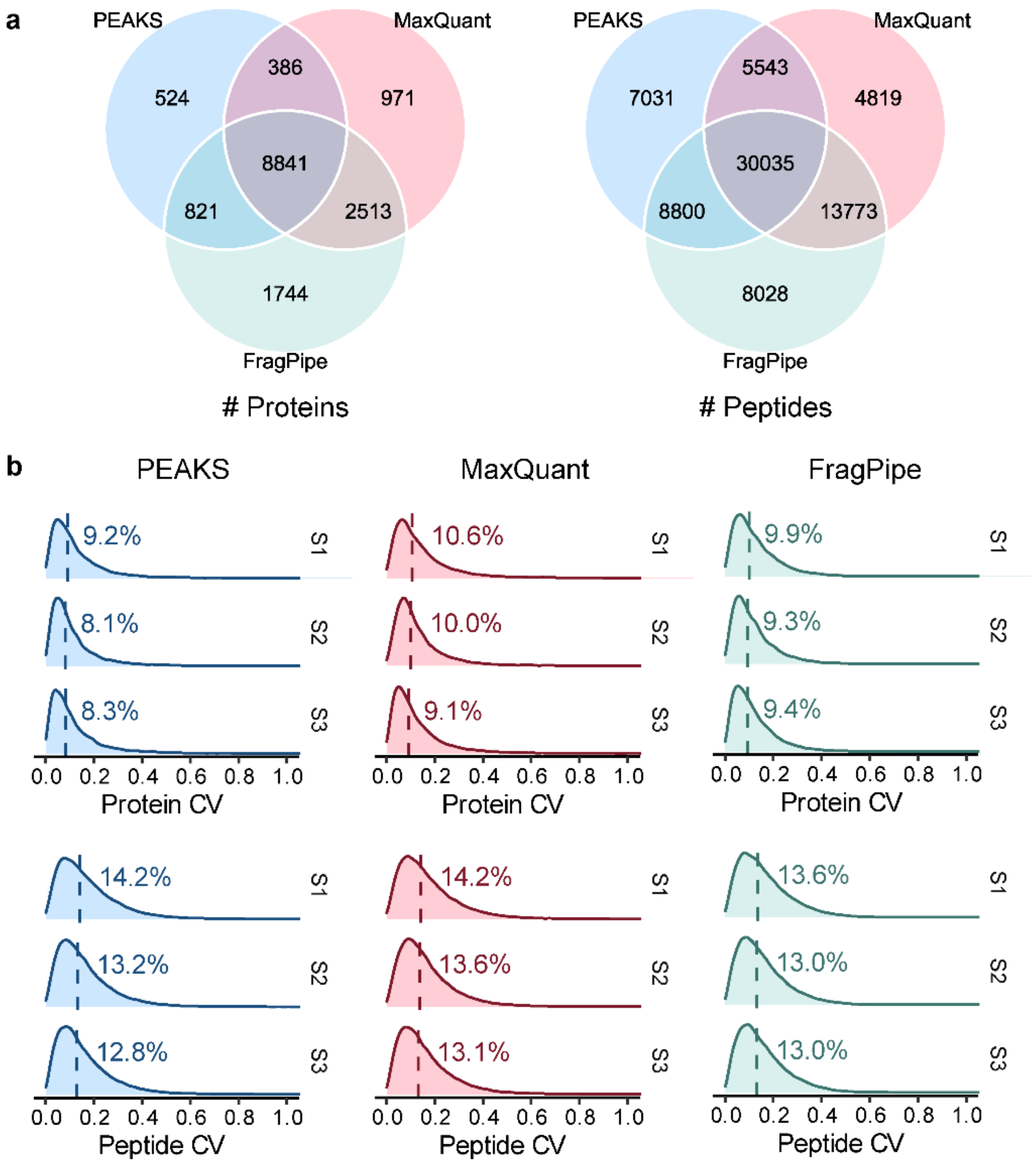

Supplementary Figure 8. Performance comparison among different software solutions for TMT data analysis on the simulated microbial communities of 12 species.

(**a**) Overlap of proteins and peptides shared in at least 2/3 replicate channels in each sample group by different software solutions. (**b**) Coefficient of variation (CV) values of protein and peptide quantification results. For each sample, only proteins and peptides quantified in all the three replicates were taken into consideration for CV calculation. The medians are indicated. Source data are provided as a Source Data file.

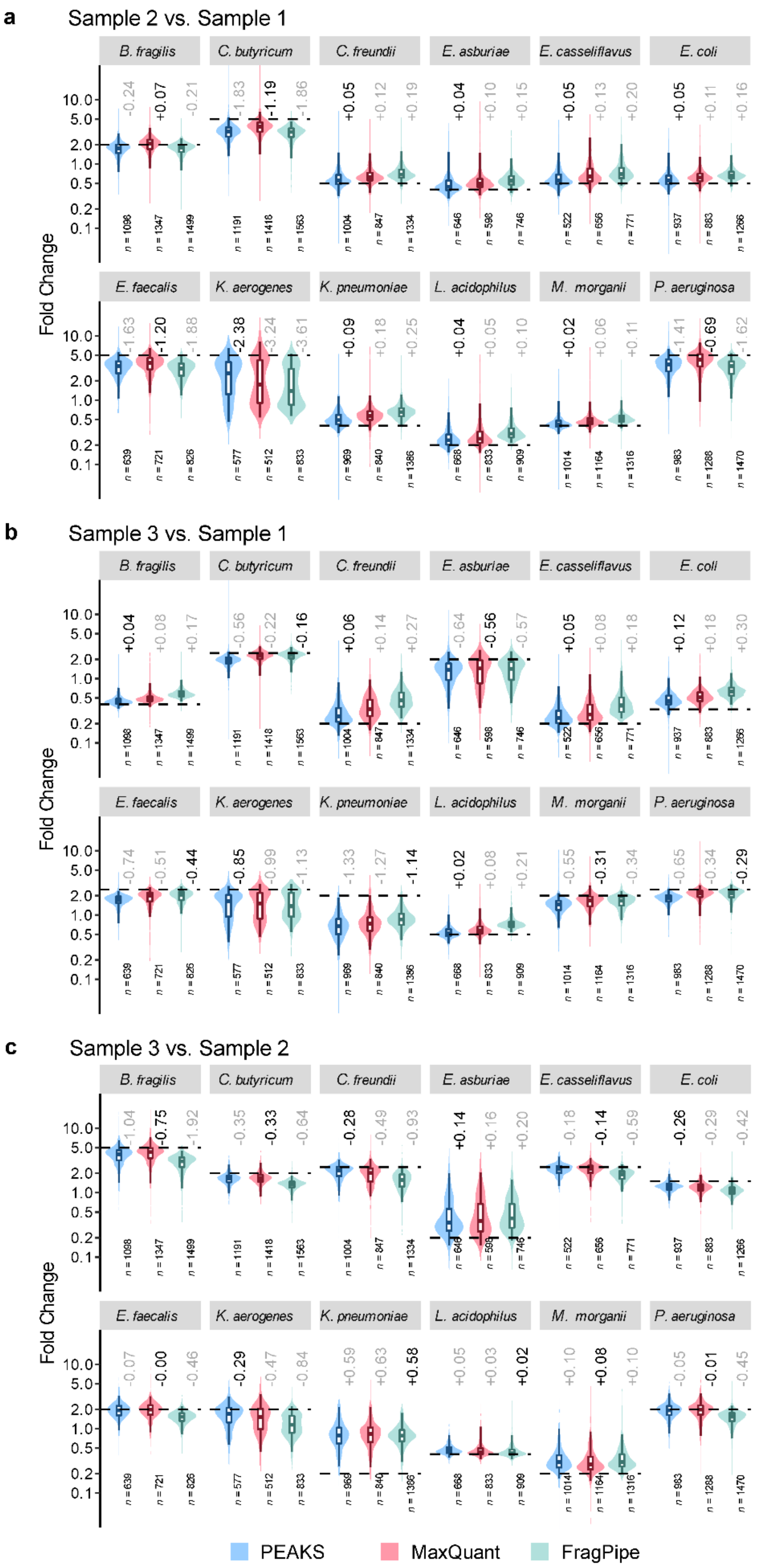
a
Sample 2 vs. Sample 1
b
Sample 3 vs. Sample 1
c
Sample 3 vs. Sample 2
B. fragilis
C. butyricum
C. freundii
E. asburiae
E. casseliflavus
E. coli
E. faecalis
K. aerogenes
K. pneumoniae
L. acidophilus
M. morganii
P. aeruginosa
Fold Change
10.0
5.0
2.0
1.0
0.5
0.2
0.1
PEAKS
MaxQuant
FragPipe

Supplementary Figure 9. Comparison of quantification accuracy among different software solutions for TMT data analysis on the simulated microbial communities of 12 species.

(**a**) Measured fold change (FC) values of protein abundance for each species between sample 2 (as numerator) and sample 1 (as denominator). (**b**) Measured FC values between sample 3 (as numerator) and sample 1 (as denominator). (**c**) Measured FC values between sample 3 (as numerator) and sample 2 (as denominator). FC values were calculated based on the average of the replicates of each sample. Only proteins quantified in at least 2/3 replicates of each sample and uniquely belonging to one species were taken into consideration. Numbers (*n*) of quantified proteins are indicated for each species. The boxes mark the first and third quantile and the lines inside the boxes mark the median; the whiskers mark 2.5% and 97.5% percentile; outliers are not shown. The theoretical ratios are highlighted as dashed lines. Differences between the measured median FC values and theoretical values are indicated, among which the smallest ones are darkened. Source data are provided as a Source Data file.

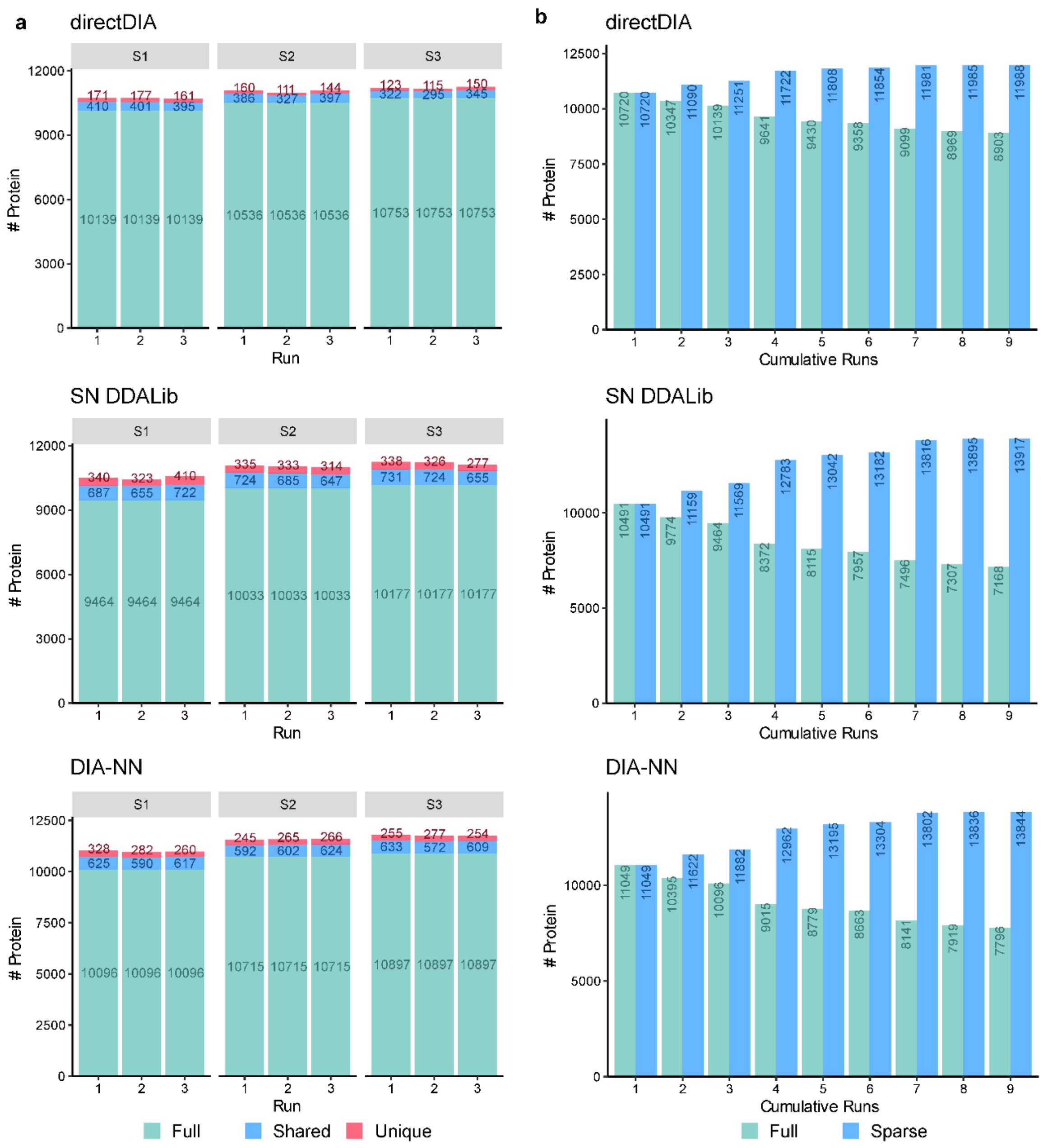

Supplementary Figure 10. Proteins quantified by different software solutions from LFQ-DIA data of the simulated microbial communities of 12 species.

(**a**) Numbers of quantified proteins per run. “Full” represents proteins quantified in all the runs of a sample; “shared” represents proteins quantified in 2 runs of a sample; “unique” represents proteins quantified in only 1 run. (**b**) Numbers of cumulative proteins from run 1 to 9 (including sample 1, sample 2 and sample 3). “Full” represents proteins shared in the cumulative runs; “sparse” represents proteins quantified in at least 1 run in the cumulative runs. SN DDALib: Spectronaut using a DDA-based spectral library.

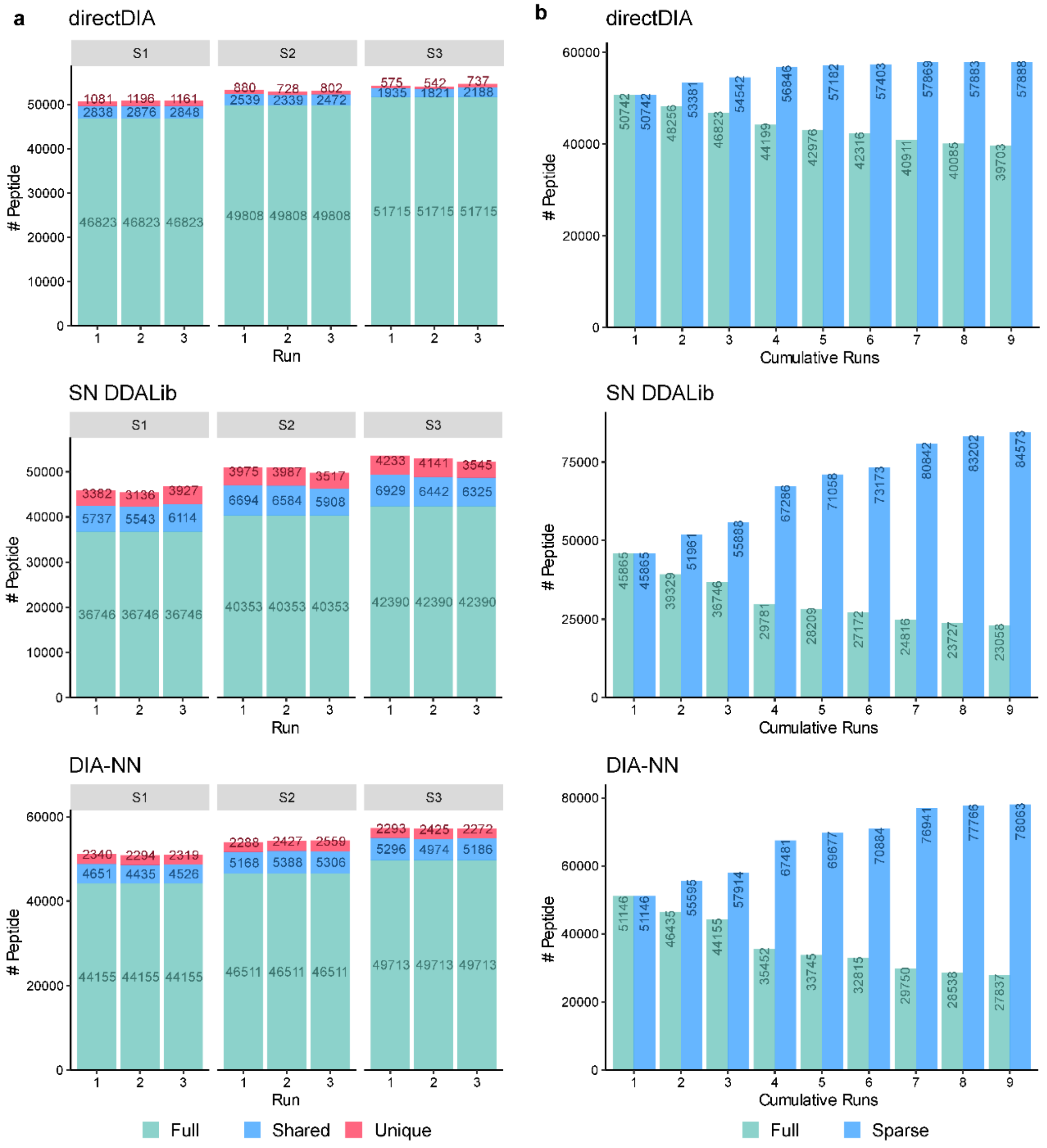

Supplementary Figure 11. Peptides quantified by different software solutions from LFQ-DIA data of the simulated microbial communities of 12 species.

(**a**) Numbers of quantified peptides per run. “Full” represents peptides quantified in all the runs of a sample; “shared” represents peptides quantified in 2 runs of a sample; “unique” represents peptides quantified in only 1 run. (**b**) Numbers of cumulative peptides from run 1 to 9 (including sample 1, sample 2 and sample 3). “Full” represents peptides shared in the cumulative runs; “sparse” represents peptides quantified in at least 1 run in the cumulative runs. SN DDALib: Spectronaut using a DDA-based spectral library.

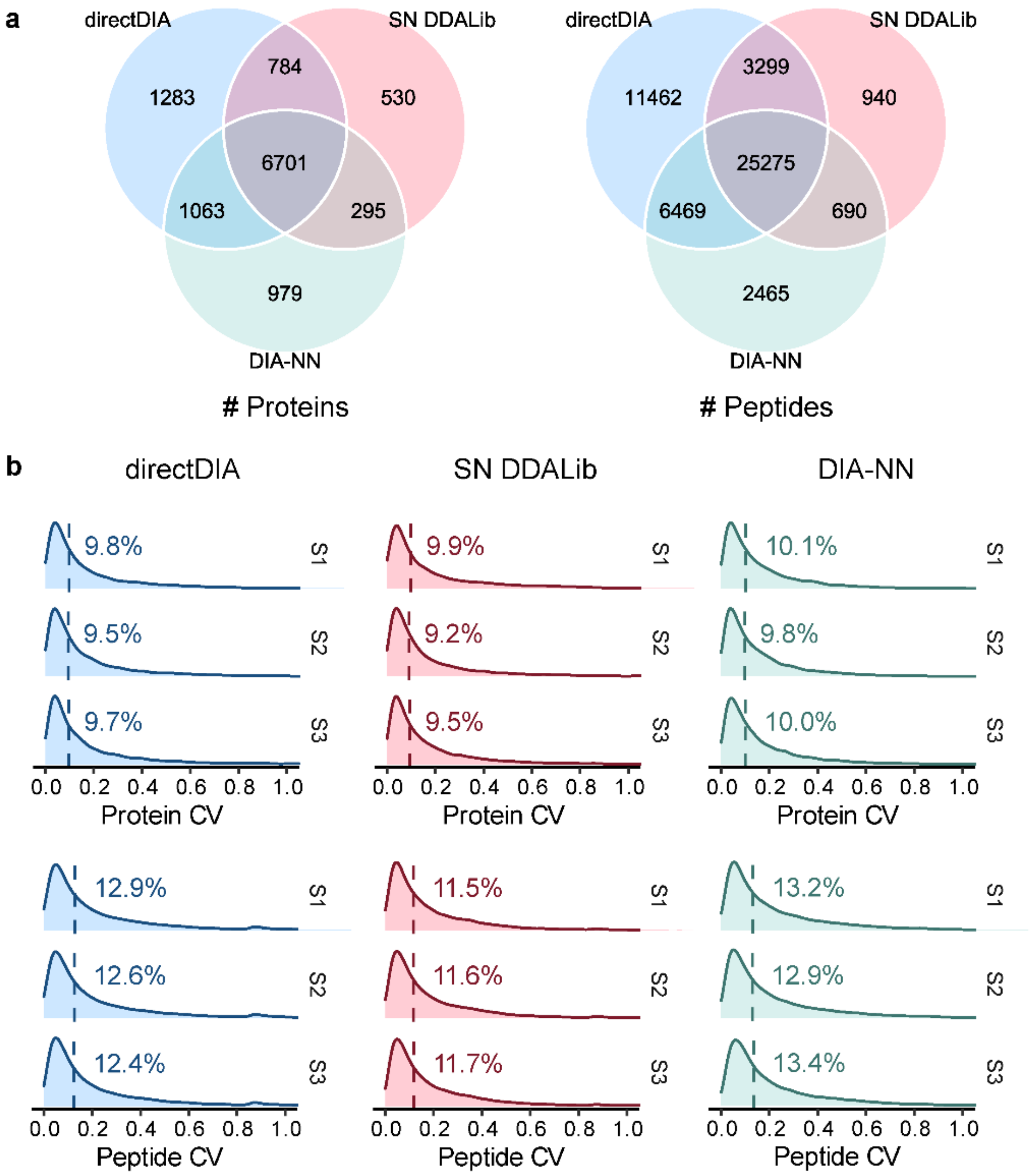

Supplementary Figure 12. Performance comparison among different software solutions for LFQ-DIA data analysis on the simulated microbial communities of 12 species.

(**a**) Overlap of proteins and peptides shared in at least 2/3 replicate runs in each sample group by different software solutions. (**b**) Coefficient of variation (CV) values of protein and peptide quantification results. For each sample, only proteins and peptides quantified in all the three replicates were taken into consideration for CV calculation. The medians are indicated. SN DDALib: Spectronaut using a DDA-based spectral library. Source data are provided as a Source Data file.

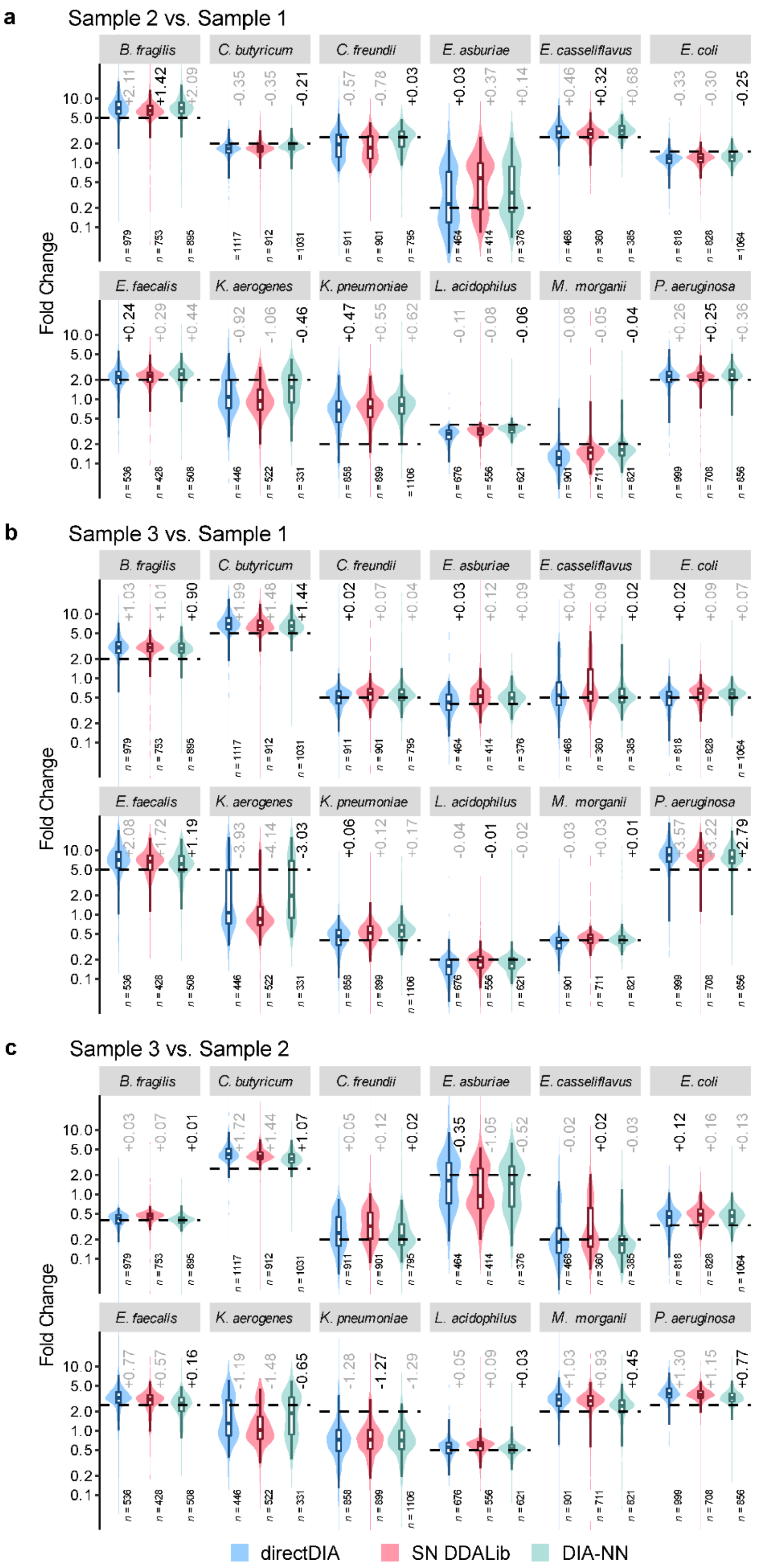
a
Sample 2 vs. Sample 1
b
Sample 3 vs. Sample 1
c
Sample 3 vs. Sample 2
B. fragilis
C. butyricum
C. freundii
E. asburiae
E. casseliflavus
E. coli
E. faecalis
K. aerogenes
K. pneumoniae
L. acidophilus
M. morganii
P. aeruginosa
Fold Change
directDIA
SN DDALib
DIA-NN

Supplementary Figure 13. Comparison of quantification accuracy among different software solutions for LFQ-DIA data analysis on the simulated microbial communities of 12 species. (**a**) Measured fold change (FC) values of protein abundance for each species between sample 2 (as numerator) and sample 1 (as denominator). (**b**) Measured FC values between sample 3 (as numerator) and sample 1 (as denominator). (**c**) Measured FC values between sample 3 (as numerator) and sample 2 (as denominator). FC values were calculated based on the average of the replicates of each sample. Only proteins quantified in at least 2/3 replicates of each sample and uniquely belonging to one species were taken into consideration. Numbers (*n*) of quantified proteins are indicated for each species. The boxes mark the first and third quantile and the lines inside the boxes mark the median; the whiskers mark 2.5% and 97.5% percentile; outliers are not shown. The theoretical ratios are highlighted as dashed lines. Differences between the measured median FC values and theoretical values are indicated, among which the smallest ones are darkened. SN DDALib: Spectronaut using a DDA-based spectral library. Source data are provided as a Source Data file.

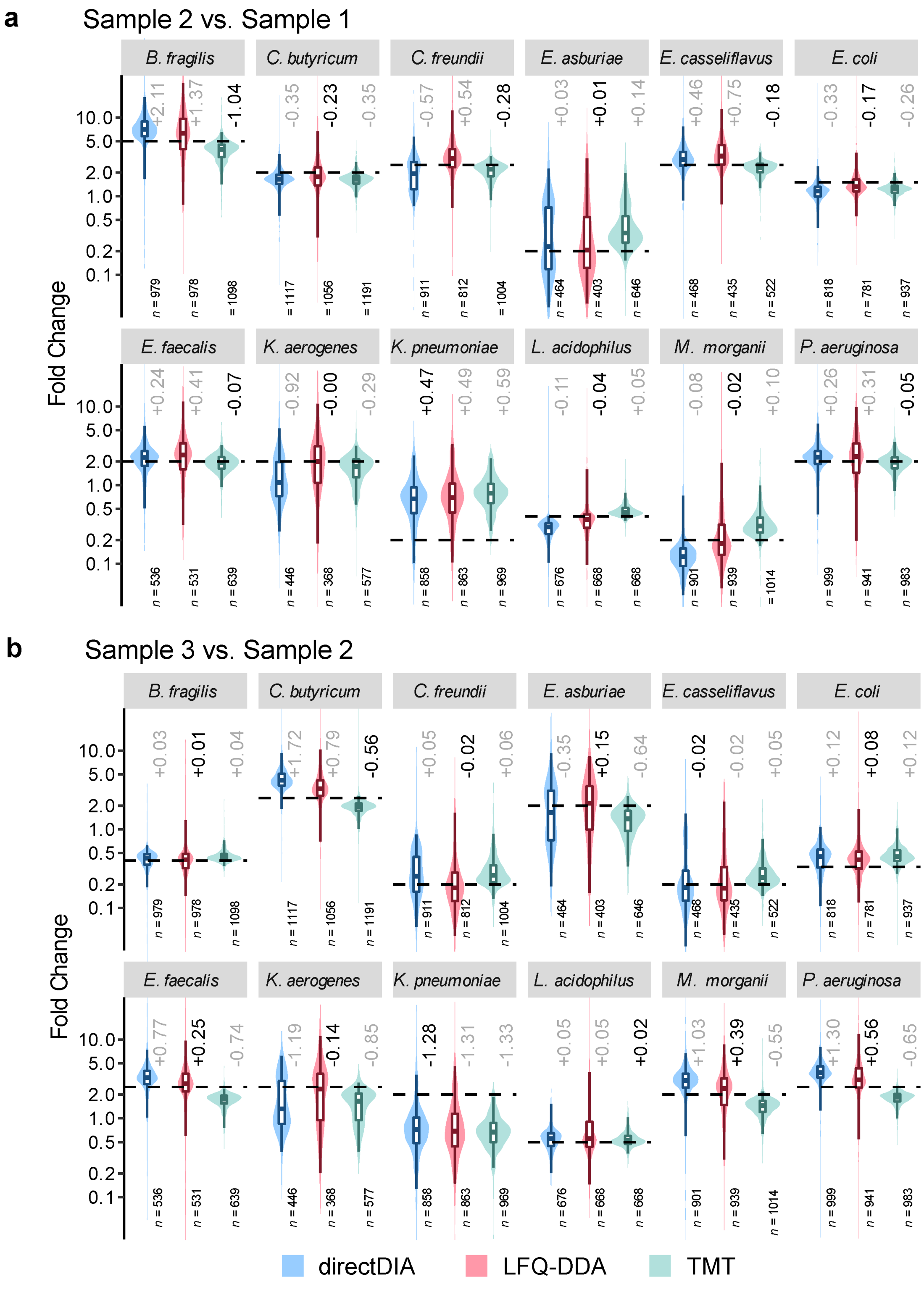
a
Sample 2 vs. Sample 1
b
Sample 3 vs. Sample 2
Fold Change
10.0
5.0
2.0
1.0
0.5
0.2
0.1
B. fragilis
C. butyricum
C. freundii
E. asburiae
E. casseliflavus
E. coli
E. faecalis
K. aerogenes
K. pneumoniae
L. acidophilus
M. morganii
P. aeruginosa
+2.11
+1.37
-1.04
n = 979
n = 978
n = 1098
-0.35
-0.23
-0.35
n = 1117
n = 1056
n = 1191
-0.57
+0.54
-0.28
n = 911
n = 812
n = 1004
+0.03
+0.01
+0.14
n = 464
n = 403
n = 646
+0.46
+0.75
-0.18
n = 468
n = 435
n = 522
-0.33
-0.17
-0.26
n = 818
n = 781
n = 937
+0.24
+0.41
-0.07
n = 536
n = 531
n = 639
-0.92
-0.00
-0.29
n = 446
n = 368
n = 577
+0.47
+0.49
+0.59
n = 858
n = 863
n = 969
-0.11
-0.04
+0.05
n = 676
n = 668
n = 668
-0.08
-0.02
+0.10
n = 901
n = 939
n = 1014
+0.26
+0.31
-0.05
n = 999
n = 941
n = 983
+0.03
+0.01
+0.04
+1.72
+0.79
-0.56
+0.05
-0.02
+0.06
-0.35
+0.15
-0.64
-0.02
-0.02
+0.05
+0.12
+0.08
+0.12
+0.77
+0.25
-0.74
-1.19
-0.14
-0.85
-1.28
-1.31
-1.33
+0.05
+0.05
+0.02
+1.03
+0.39
-0.55
+1.30
+0.56
-0.65
directDIA
LFQ-DDA
TMT

Supplementary Figure 14. Comparison of quantification accuracy between directDIA, LFQ-DDA, and TMT on the simulated microbial community of 12 species.

(**a**) Measured fold change (FC) values of protein abundance for each species between sample 2 (as numerator) and sample 1 (as denominator). (**b**) Measured FC values between sample 3 (as numerator) and sample 2 (as denominator). Measured FC values between sample 3 (as numerator) and sample 1 (as denominator) are shown in main **Figure 2b**. FC values were calculated based on the average of the replicates of each sample. Only proteins quantified in at least 2/3 replicates of each sample and uniquely belonging to one species were taken into consideration. Numbers (*n*) of quantified proteins are indicated for each species. The boxes mark the first and third quantile and the lines inside the boxes mark the median; the whiskers mark 2.5% and 97.5% percentile; outliers are not shown. The theoretical ratios are highlighted as dashed lines. Differences between the measured median FC values and theoretical values are indicated, among which the smallest ones are darkened. The DDA and TMT data were analyzed by PEAKS. Source data are provided as a Source Data file.

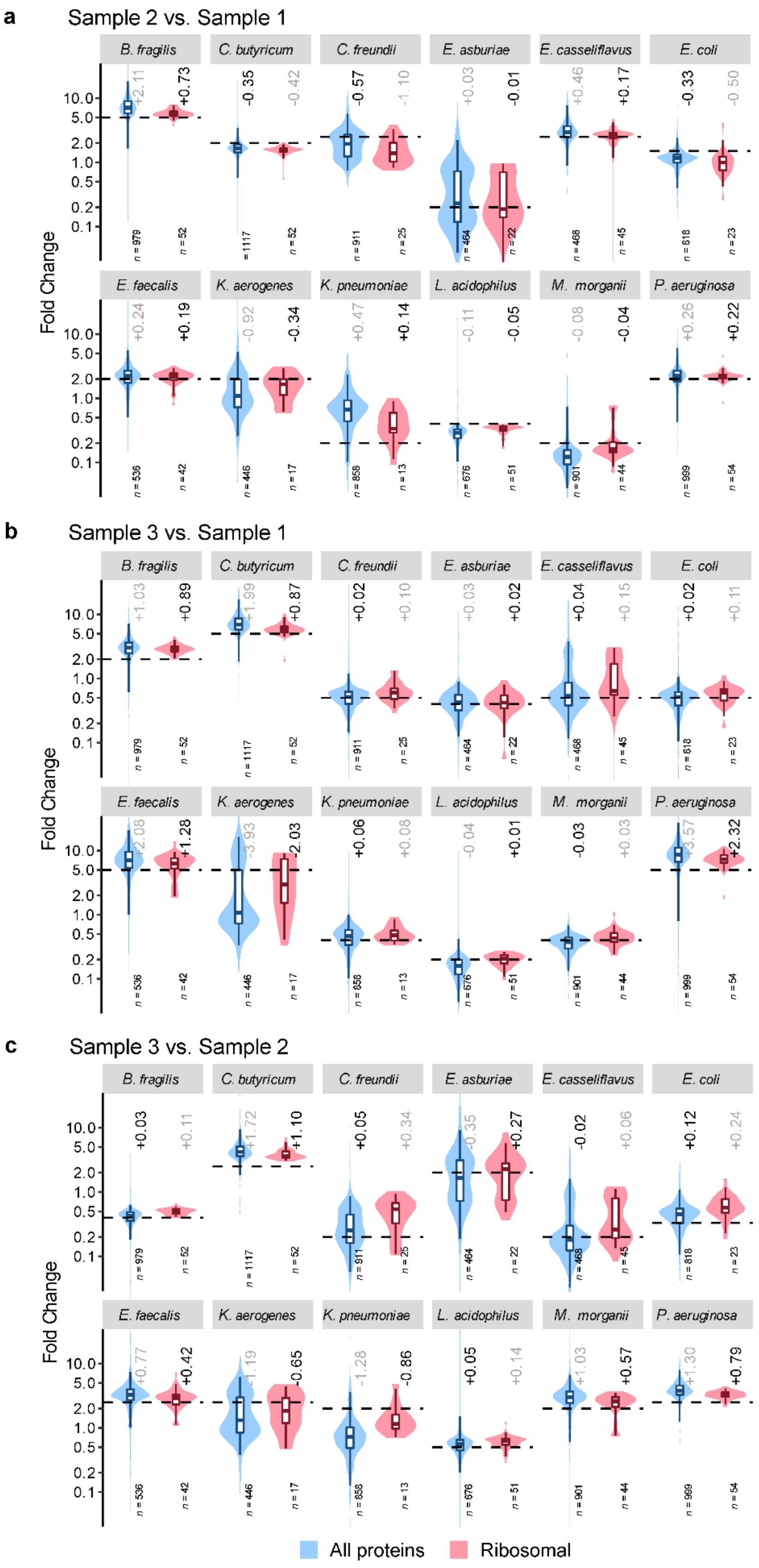
a
Sample 2 vs. Sample 1
B. fragilis
C. butyricum
C. freundii
E. asburiae
E. casseliflavus
E. coli
E. faecalis
K. aerogenes
K. pneumoniae
L. acidophilus
M. morganii
P. aeruginosa
+2.11
+0.73
-0.35
-0.42
-0.57
-1.10
+0.03
-0.01
+0.46
+0.17
-0.33
-0.50
+0.24
+0.19
-0.92
-0.34
+0.47
+0.14
-0.11
-0.05
-0.08
-0.04
+0.26
+0.22
Fold Change
10.0
5.0
2.0
1.0
0.5
0.2
0.1
b
Sample 3 vs. Sample 1
+1.03
+0.89
+1.99
+0.87
+0.02
+0.10
+0.03
+0.02
+0.04
+0.15
+0.02
+0.11
+2.08
+1.28
-3.93
-2.03
+0.06
+0.08
-0.04
+0.01
-0.03
+0.03
+3.57
+2.32
c
Sample 3 vs. Sample 2
+0.03
+0.11
+1.72
+1.10
+0.05
+0.34
-0.35
+0.27
-0.02
+0.06
+0.12
+0.24
+0.77
+0.42
-1.19
-0.65
-1.28
-0.86
+0.05
+0.14
+1.03
+0.57
+1.30
+0.79
All proteins
Ribosomal

Supplementary Figure 15. Comparison of quantification accuracy between ribosomal proteins and all proteins using directDIA on the simulated microbial communities of 12 species.

(**a**) Measured fold change (FC) values of protein abundance for each species between sample 2 (as numerator) and sample 1 (as denominator). (**b**) Measured FC values between sample 3 (as numerator) and sample 1 (as denominator). (**c**) Measured FC values between sample 3 (as numerator) and sample 2 (as denominator). FC values were calculated based on the average of the replicates of each sample. Only proteins quantified in at least 2/3 replicates of each sample and uniquely belonging to one species were taken into consideration. Numbers (*n*) of quantified proteins are indicated for each species. The boxes mark the first and third quantile and the lines inside the boxes mark the median; the whiskers mark 2.5% and 97.5% percentile; outliers are not shown. The theoretical ratios are highlighted as dashed lines. Differences between the measured median FC values and theoretical values are indicated, among which the smallest ones are darkened. Source data are provided as a Source Data file.

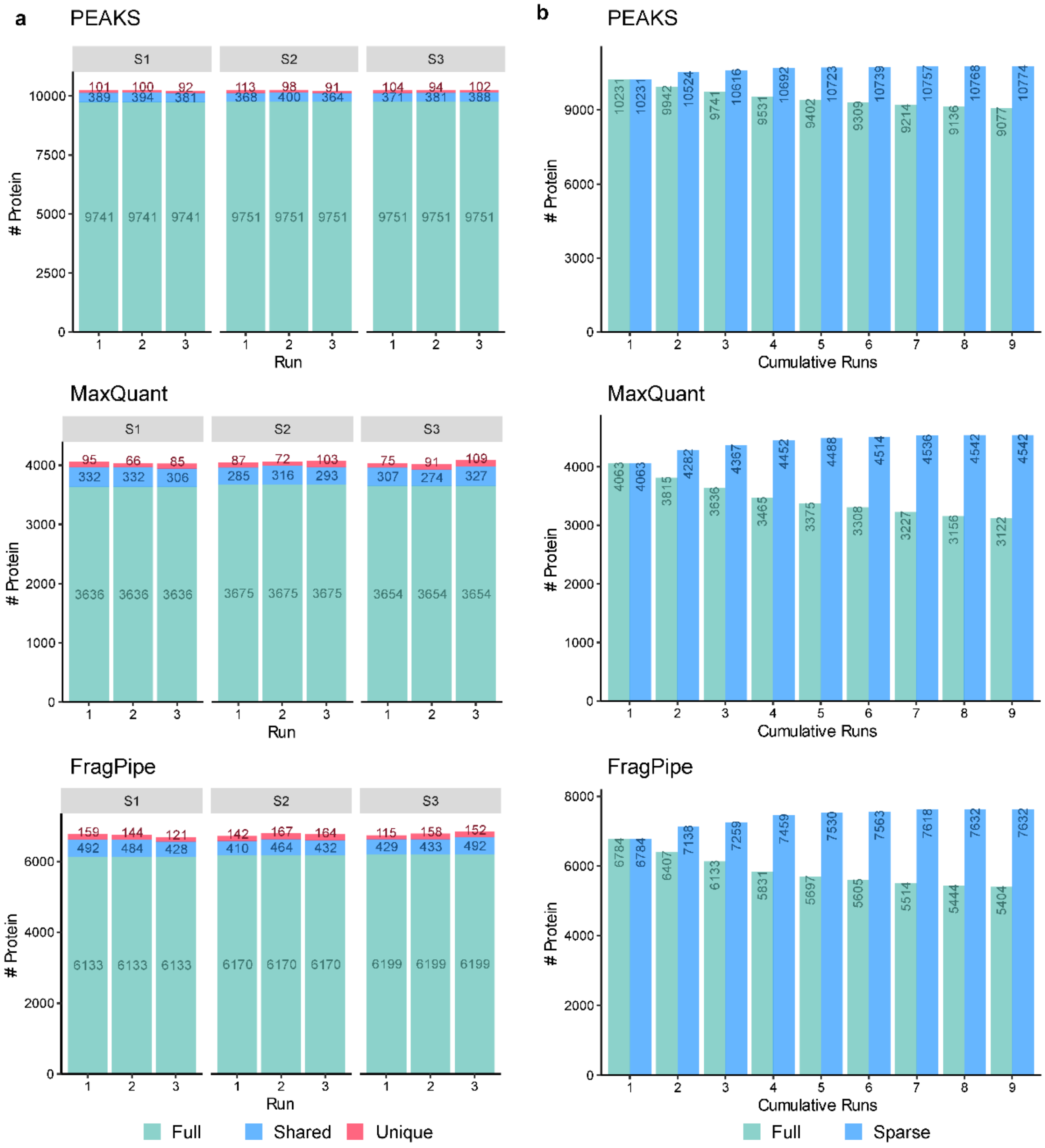

Supplementary Figure 16. Proteins quantified by different software solutions from LFQ-DDA data of the spike-in metaproteome samples.

(**a**) Numbers of quantified proteins per run. “Full” represents proteins quantified in all the runs of a sample; “shared” represents proteins quantified in 2 runs of a sample; “unique” represents proteins quantified in only 1 run. (**b**) Numbers of cumulative proteins from run 1 to 9 (including sample 1, sample 2 and sample 3). “Full” represents proteins shared in the cumulative runs; “sparse” represents proteins quantified in at least 1 run in the cumulative runs.

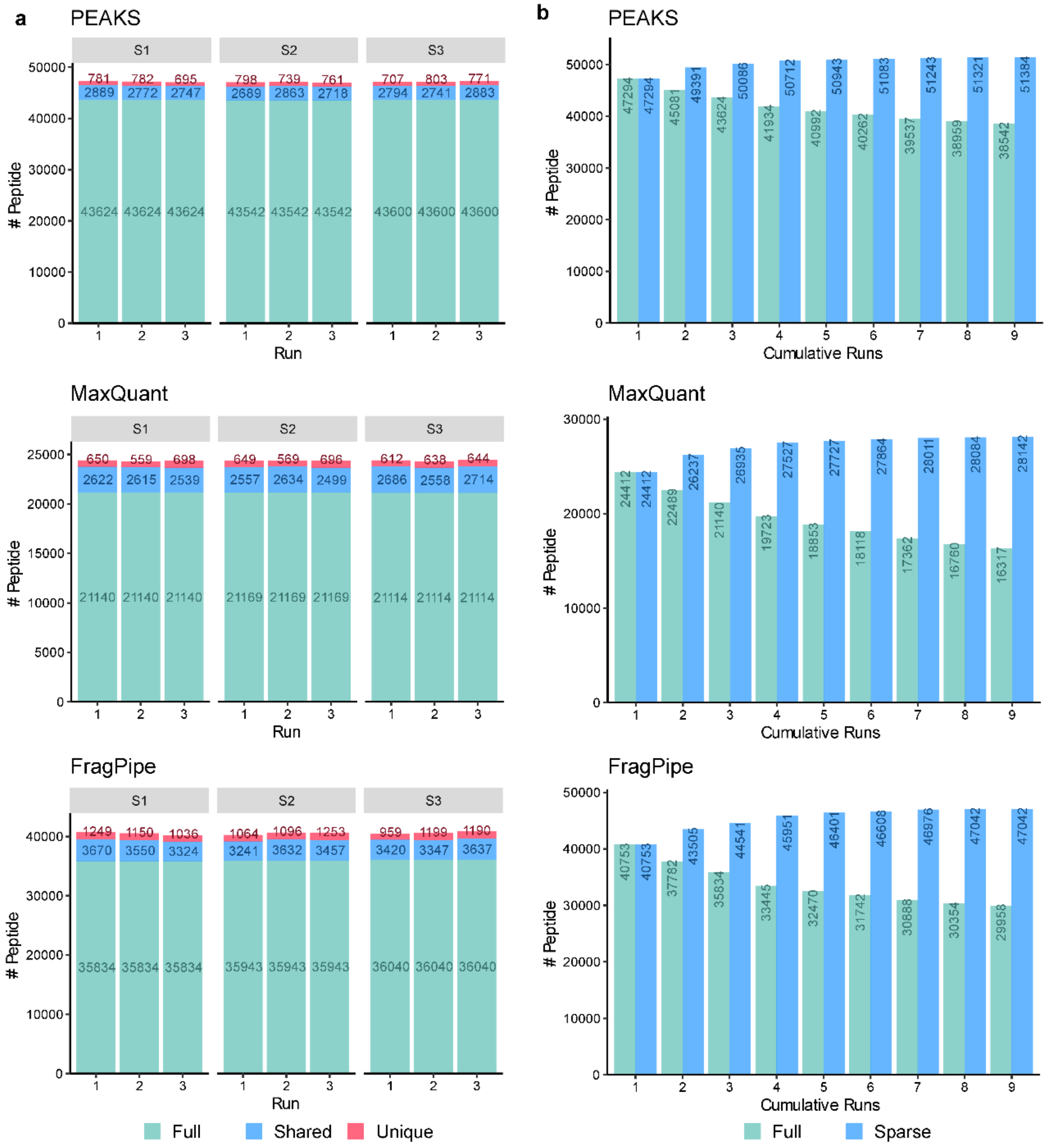

Supplementary Figure 17. Peptides quantified by different software solutions from LFQ-DDA data of the spike-in metaproteome samples.

(**a**) Numbers of quantified peptides per run. “Full” represents peptides quantified in all the runs of a sample; “shared” represents peptides quantified in 2 runs of a sample ; “unique” represents peptides quantified in only 1 run. (**b**) Numbers of cumulative peptides from run 1 to 9 (including sample 1, sample 2 and sample 3). “Full” represents peptides shared in the cumulative runs; “sparse” represents peptides quantified in at least 1 run in the cumulative runs.

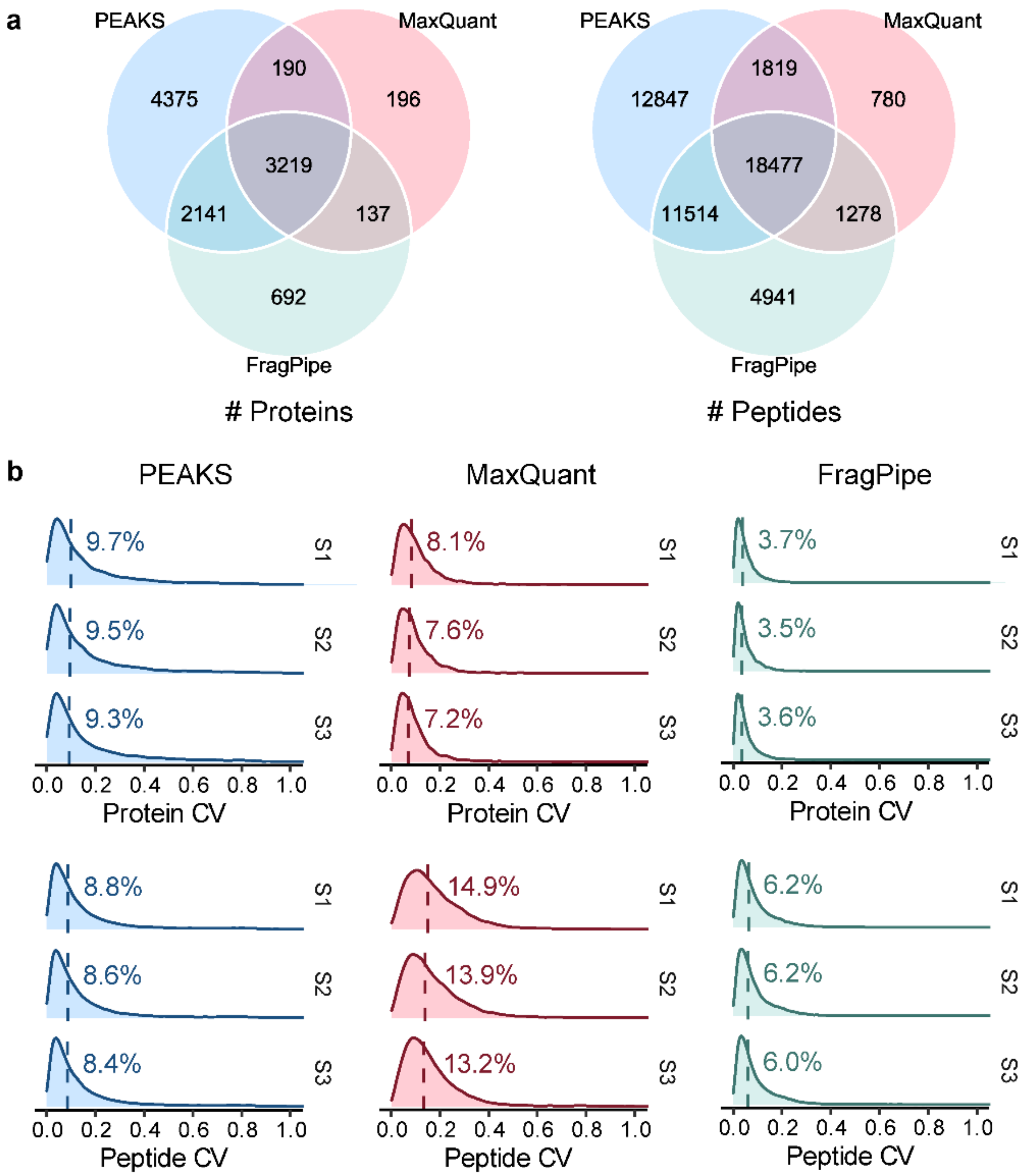

Supplementary Figure 18. Performance comparison among different software solutions for LFQ-DDA data analysis on the spike-in metaproteome samples.

(**a**) Overlap of proteins and peptides shared in at least 2/3 replicate runs in each sample group by different software solutions. (**b**) Coefficient of variation (CV) values of protein and peptide quantification results. For each sample, only proteins and peptides quantified in all the three replicates were taken into consideration for CV calculation. The medians are indicated. Source data are provided as a Source Data file.

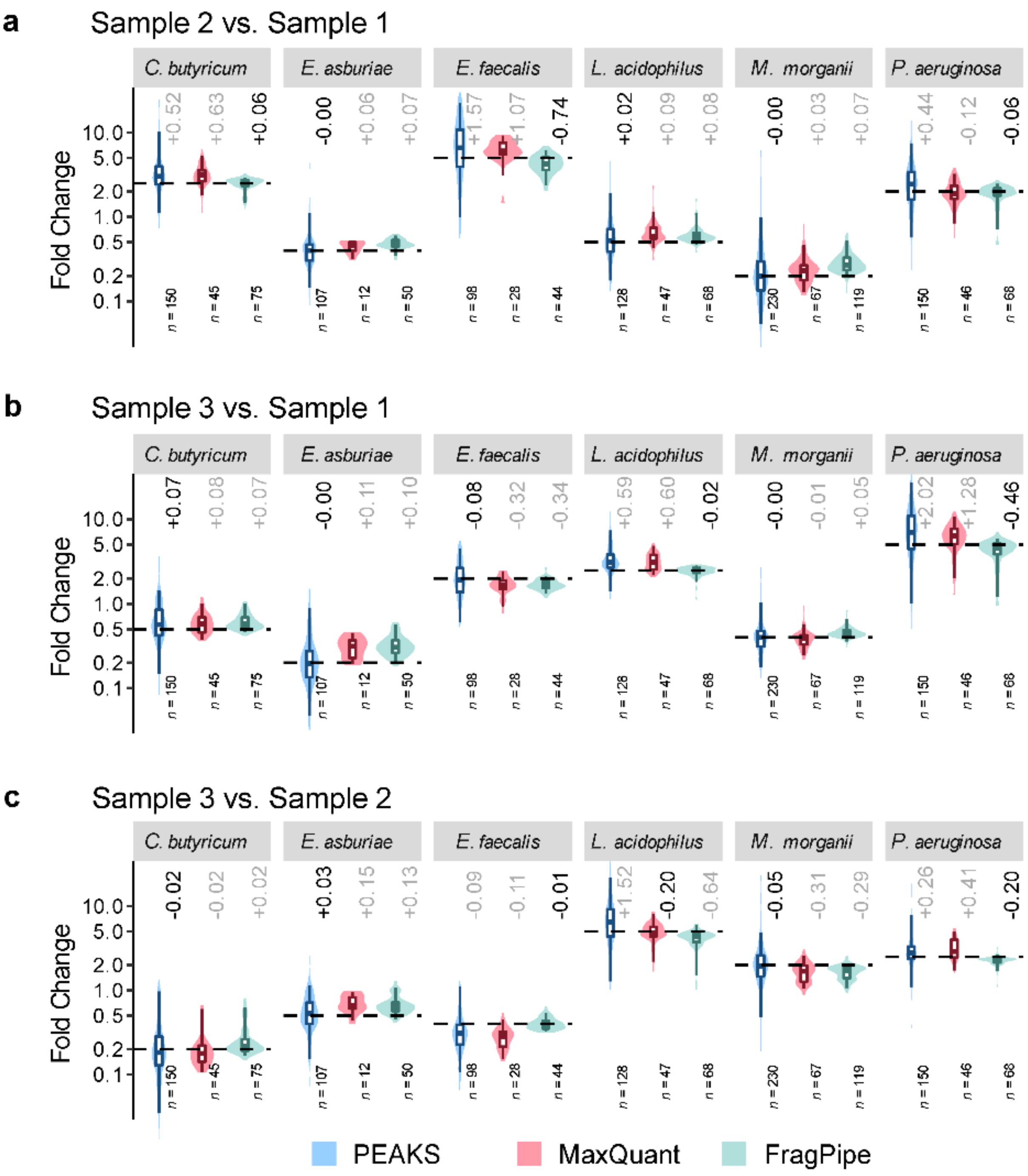

Supplementary Figure 19. Comparison of quantification accuracy among different software solutions for LFQ-DDA data analysis on the spike-in metaproteome samples.

(**a**) Measured fold change (FC) values of protein abundance for each species between sample 2 (as numerator) and sample 1 (as denominator). (**b**) Measured FC values between sample 3 (as numerator) and sample 1 (as denominator). (**c**) Measured FC values between sample 3 (as

numerator) and sample 2 (as denominator). FC values were calculated based on the average of the replicates of each sample. Only proteins quantified in at least 2/3 replicates of each sample and uniquely belonging to one species were taken into consideration. Numbers (*n*) of quantified proteins are indicated for each species. The boxes mark the first and third quantile and the lines inside the boxes mark the median; the whiskers mark 2.5% and 97.5% percentile; outliers are not shown. The theoretical ratios are highlighted as dashed lines. Differences between the measured median FC values and theoretical values are indicated, among which the smallest ones are darkened. Source data are provided as a Source Data file.

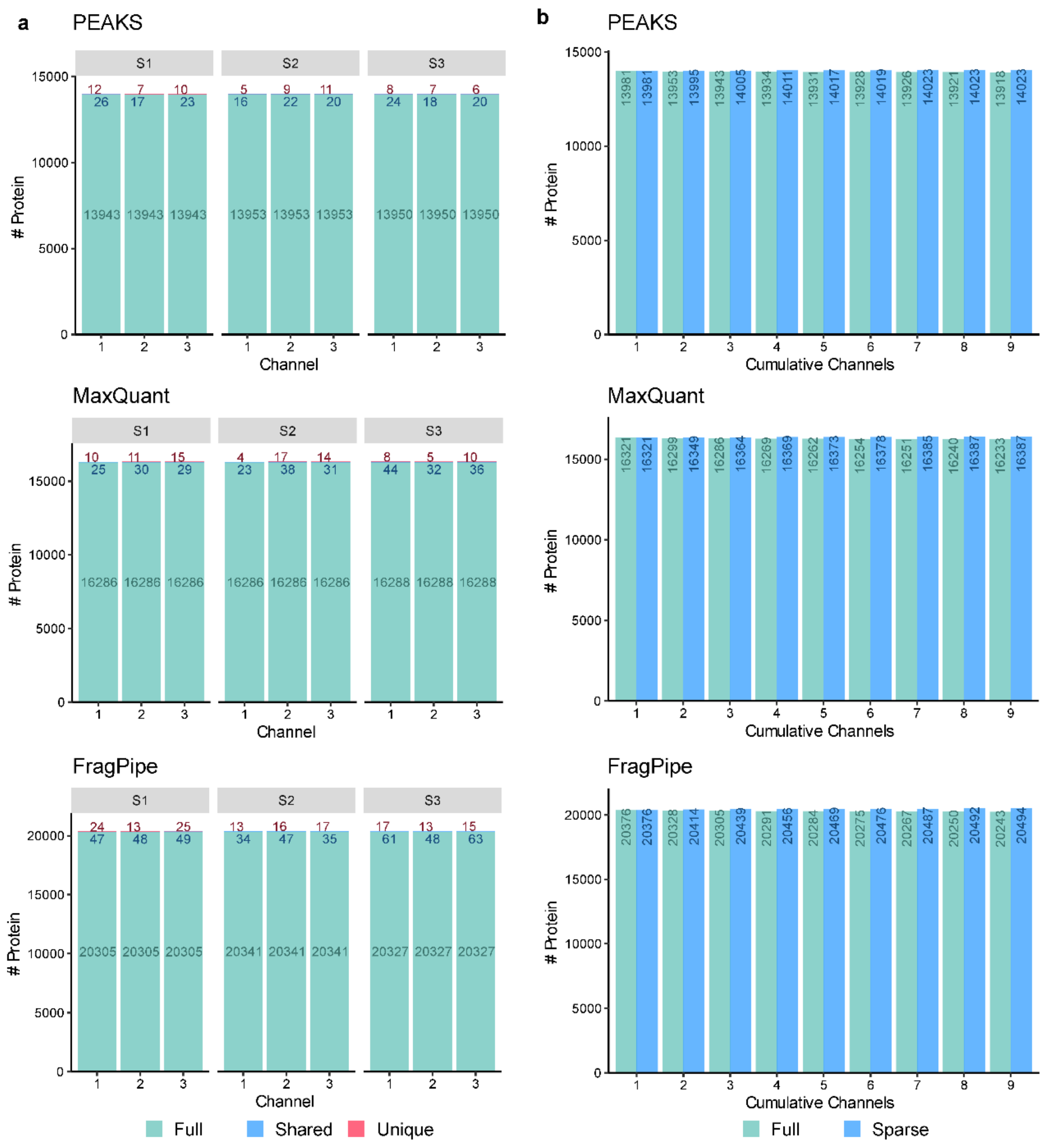

Supplementary Figure 20. Proteins quantified by different software solutions from TMT data of the spike-in metaproteome samples.

(**a**) Numbers of quantified proteins per channel. “Full” represents proteins quantified in all the channels of a sample; “shared” represents proteins quantified in 2 channels of a sample; “unique” represents proteins quantified in only 1 channel. (**b**) Numbers of cumulative proteins from channel 1 to 9 (including sample 1, sample 2, and sample 3). “Full” represents proteins shared in the cumulative channels; “sparse” represents proteins quantified in at least 1 channel in the cumulative channels.

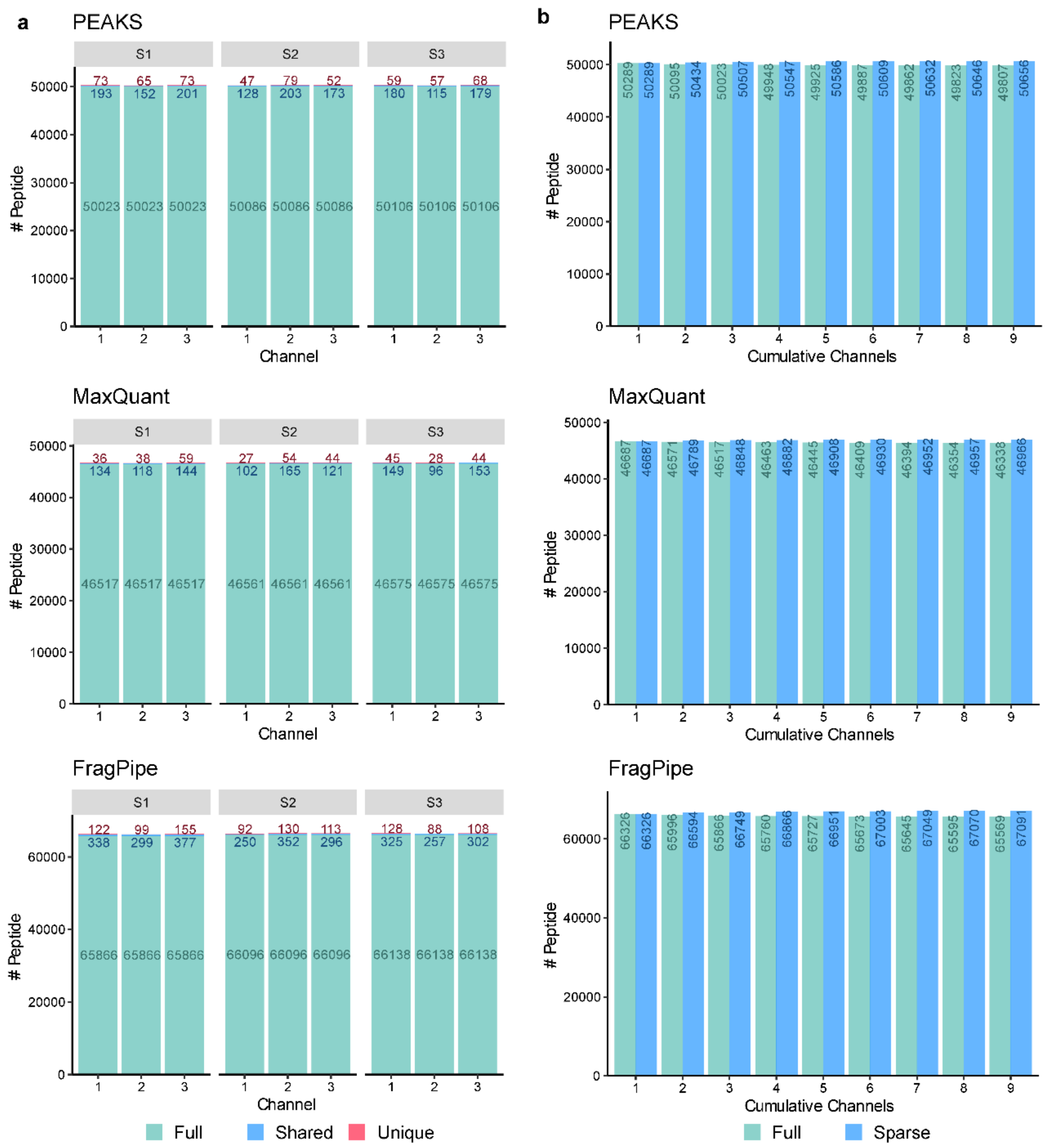

Supplementary Figure 21. Peptides quantified by different software solutions from TMT data of the spike-in metaproteome samples.

(**a**) Numbers of quantified peptides per channel. "Full" represents peptides quantified in all the channels of a sample; "shared" represents peptides quantified in 2 channels of a sample; "unique" represents peptides quantified in only 1 channel. (**b**) Numbers of cumulative peptides from channel 1 to 9 (including sample 1, sample 2, and sample 3). "Full" represents peptides shared in the cumulative channels; "sparse" represents peptides quantified in at least 1 channel in the cumulative channels.

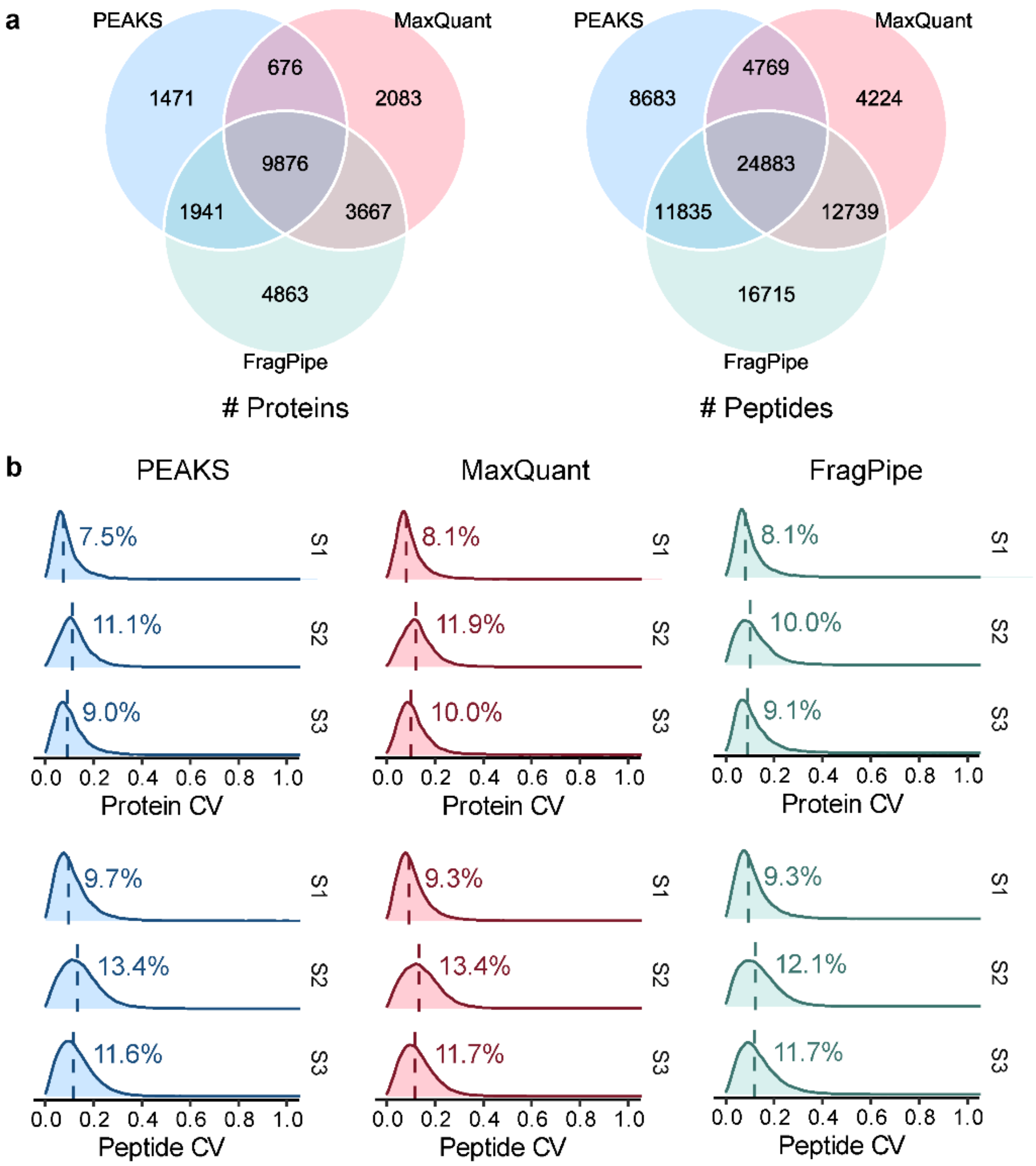

Supplementary Figure 22. Performance comparison among different software solutions for TMT data analysis on the spike-in metaproteome samples.

(**a**) Overlap of proteins and peptides shared in at least 2/3 replicate channels in each sample group by different software solutions. (**b**) Coefficient of variation (CV) values of protein and peptide quantification results. For each sample, only proteins and peptides quantified in all the three replicates were taken into consideration for CV calculation. The medians are indicated. Source data are provided as a Source Data file.

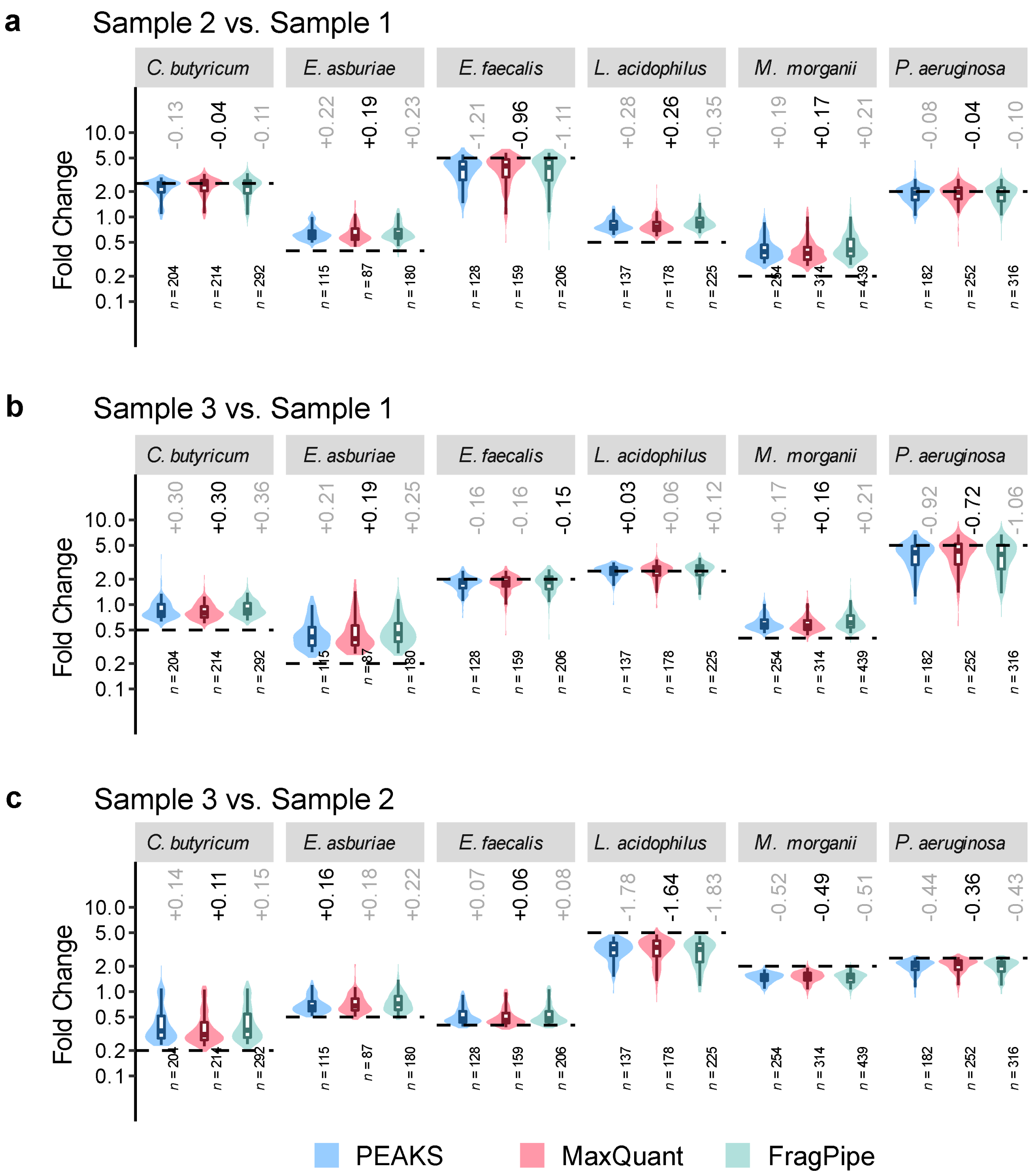

Supplementary Figure 23. Comparison of quantification accuracy among different software solutions for TMT data analysis on the spike-in metaproteome samples.

(**a**) Measured fold change (FC) values of protein abundance for each species between sample 2 (as numerator) and sample 1 (as denominator). (**b**) Measured FC values between sample 3 (as numerator) and sample 1 (as denominator). (**c**) Measured FC values between sample 3 (as numerator) and sample 2 (as denominator). FC values were calculated based on the average of the

replicates of each sample. Only proteins quantified in at least 2/3 replicates of each sample and uniquely belonging to one species were taken into consideration. Numbers (*n*) of quantified proteins are indicated for each species. The boxes mark the first and third quantile and the lines inside the boxes mark the median; the whiskers mark 2.5% and 97.5% percentile; outliers are not shown. The theoretical ratios are highlighted as dashed lines. Differences between the measured median FC values and theoretical values are indicated, among which the smallest ones are darkened. Source data are provided as a Source Data file.

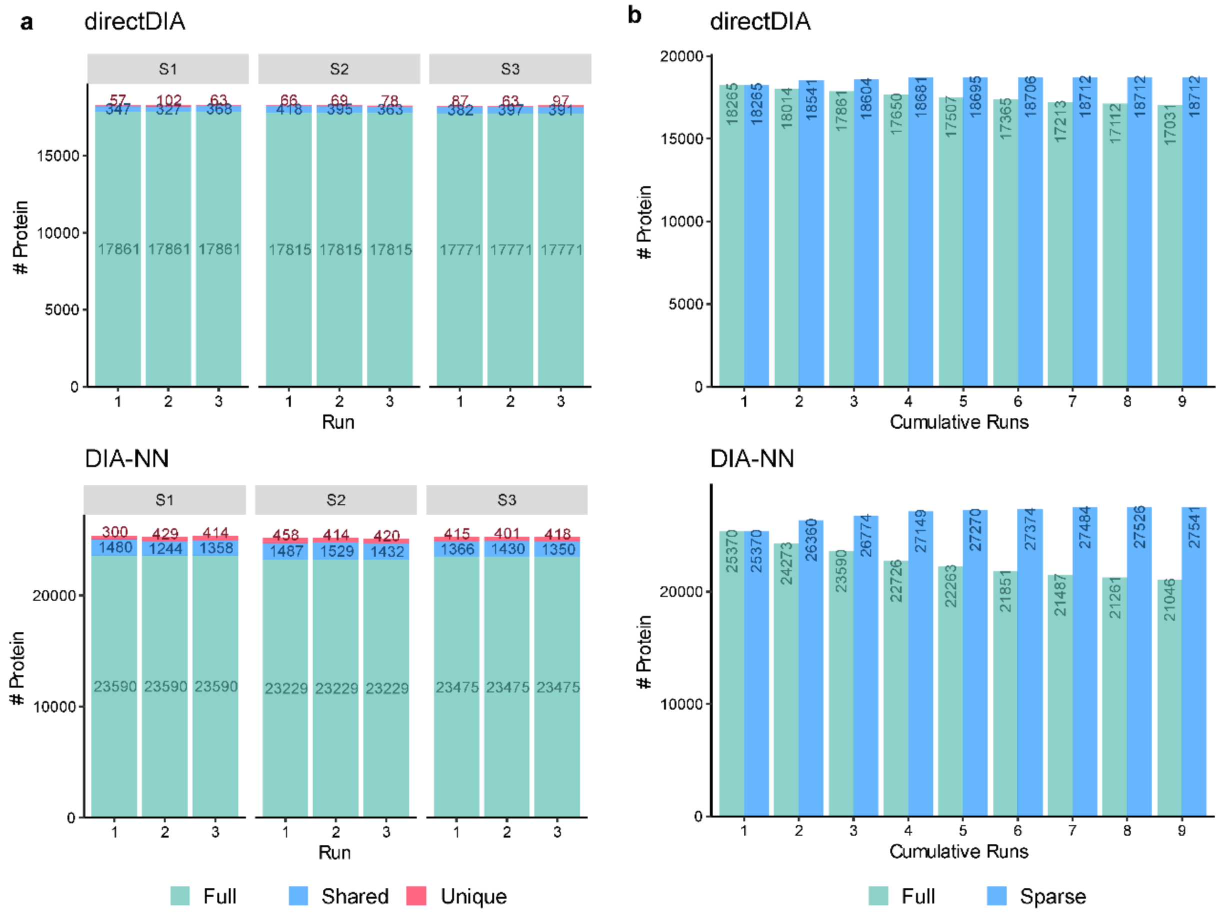

Supplementary Figure 24. Proteins quantified by different software solutions from LFQ-DIA data of the spike-in metaproteome samples.

(**a**) Numbers of quantified proteins per run. "Full" represents proteins quantified in all the runs of a sample; "shared" represents proteins quantified in 2 runs of a sample; "unique" represents proteins quantified in only 1 run. (**b**) Numbers of cumulative proteins from run 1 to 9 (including sample 1, sample 2 and sample 3). "Full" represents proteins shared in the cumulative runs; "sparse" represents proteins quantified in at least 1 run in the cumulative runs.

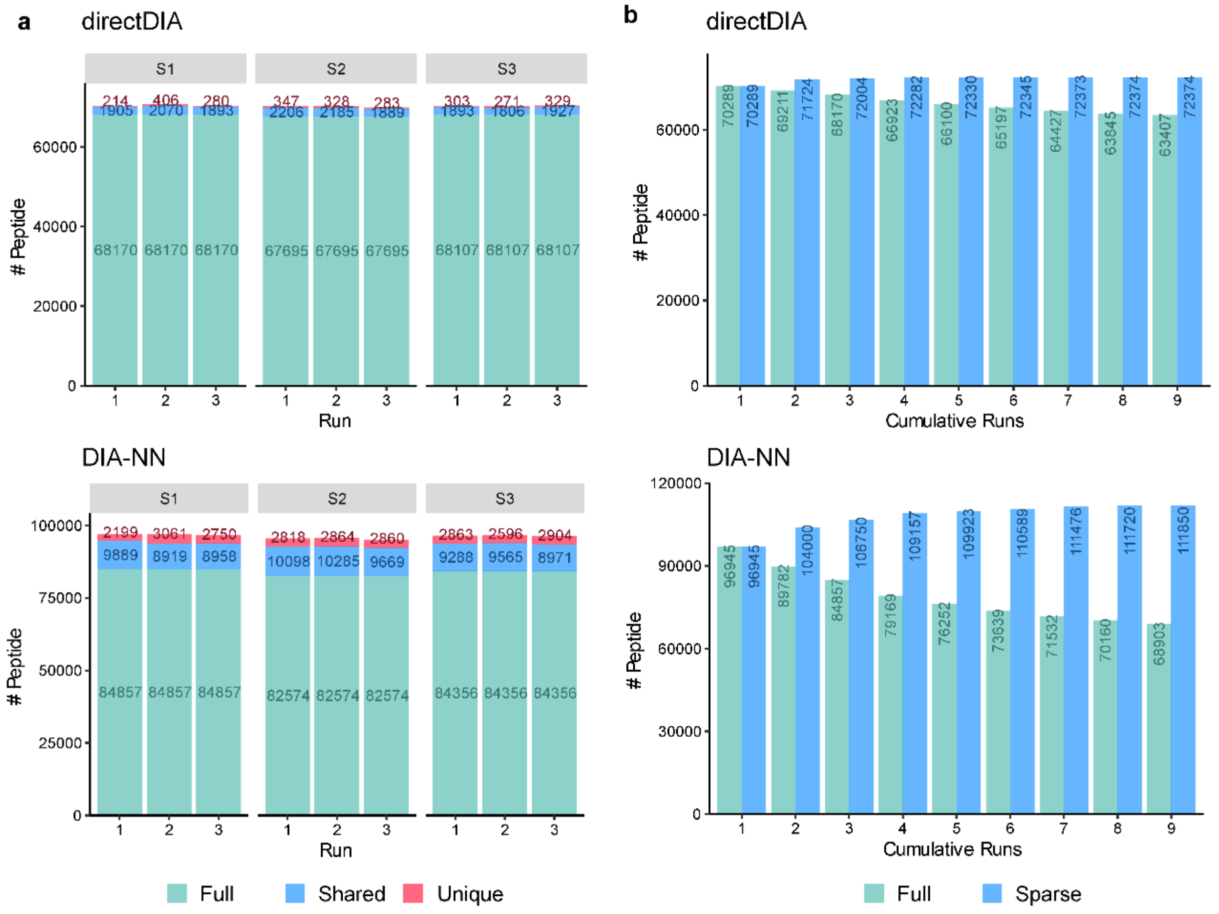

Supplementary Figure 25. Peptides quantified by different software solutions from LFQ-DIA data of the spike-in metaproteome samples.

(**a**) Numbers of quantified peptides per run. “Full” represents peptides quantified in all the runs of a sample; “shared” represents peptides quantified in 2 runs of a sample; “unique” represents peptides quantified in only 1 run. (**b**) Numbers of cumulative peptides from run 1 to 9 (including sample 1, sample 2 and sample 3). “Full” represents peptides shared in the cumulative runs; “sparse” represents peptides quantified in at least 1 run in the cumulative runs.

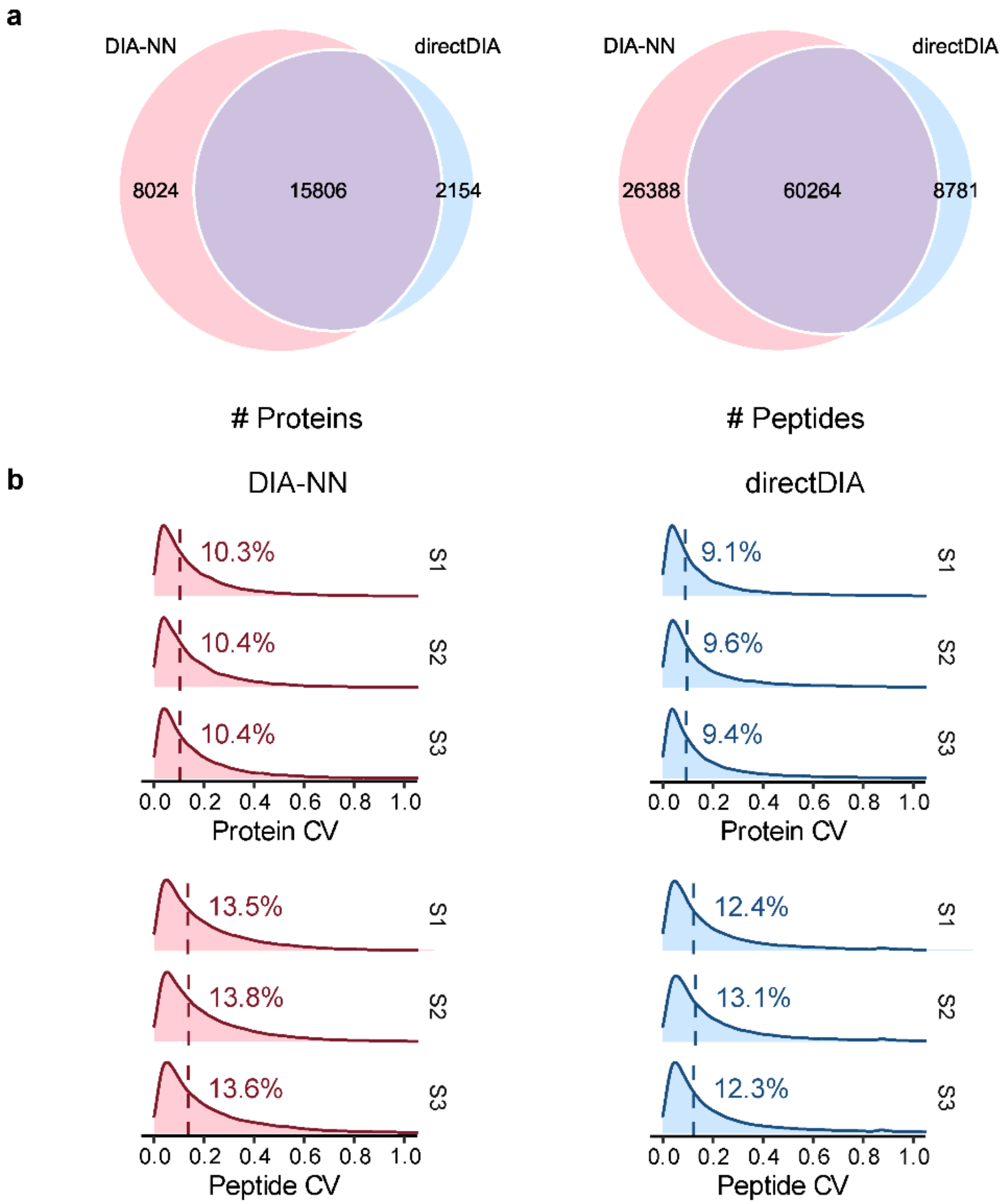

Supplementary Figure 26. Performance comparison between different software solutions for LFQ-DIA data analysis on the spike-in metaproteome samples.

(**a**) Overlap of proteins and peptides shared in at least 2/3 replicate runs in each sample group by different software solutions. (**b**) Coefficient of variation (CV) values of protein and peptide quantification results. For each sample, only proteins and peptides quantified in all the three replicates were taken into consideration for CV calculation. The medians are indicated. Source data are provided as a Source Data file.

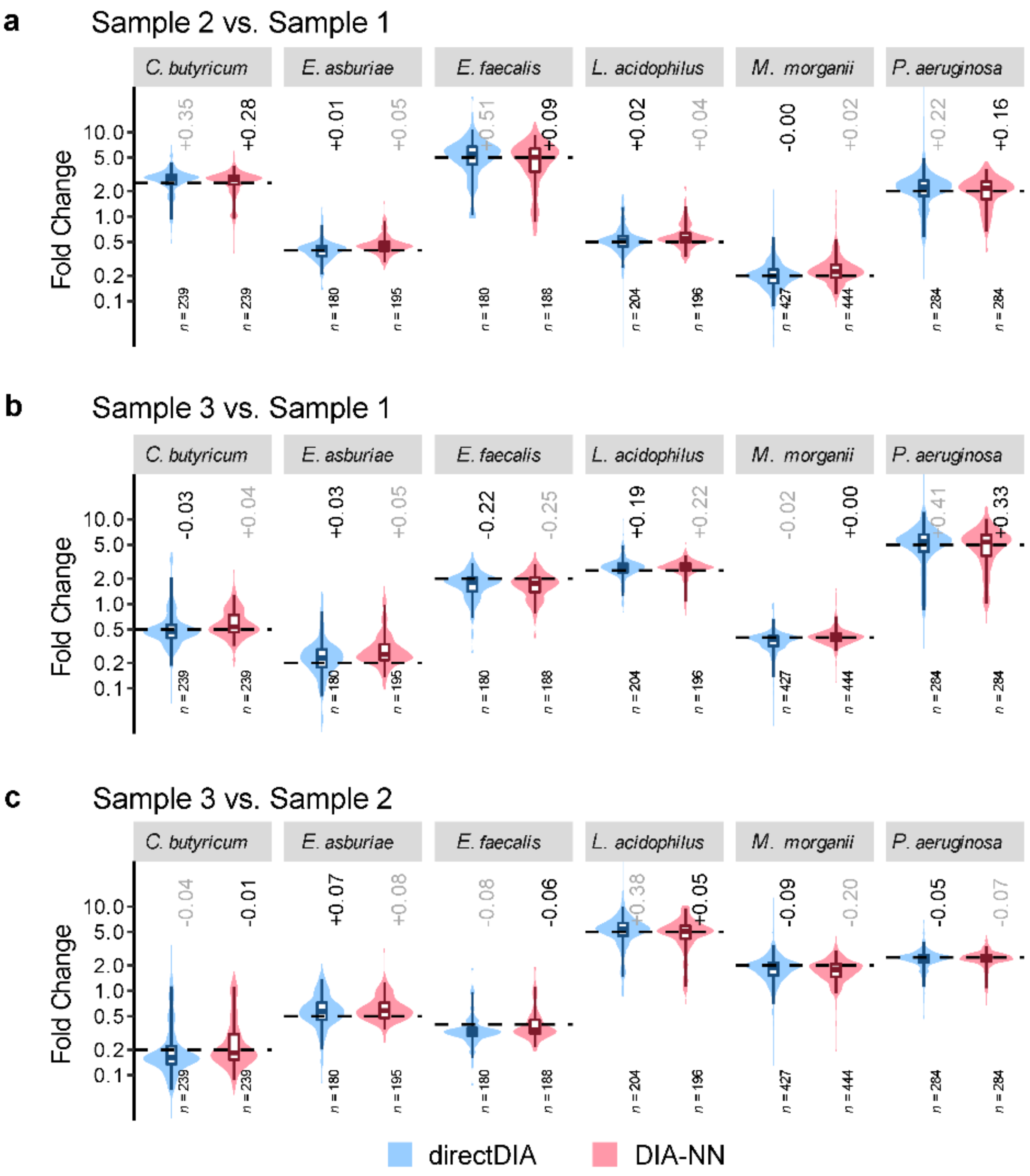

Supplementary Figure 27. Comparison of quantification accuracy between different software solutions for LFQ-DIA data analysis on the spike-in metaproteome samples.

(**a**) Measured fold change (FC) values of protein abundance for each species between sample 2 (as numerator) and sample 1 (as denominator). (**b**) Measured FC values between sample 3 (as numerator) and sample 1 (as denominator). (**c**) Measured FC values between sample 3 (as numerator) and sample 2 (as denominator). FC values were calculated based on the average of the

replicates of each sample. Only proteins quantified in at least 2/3 replicates of each sample and uniquely belonging to one species were taken into consideration. Numbers (n) of quantified proteins are indicated for each species. The boxes mark the first and third quantile and the lines inside the boxes mark the median; the whiskers mark 2.5% and 97.5% percentile; outliers are not shown. The theoretical ratios are highlighted as dashed lines. Differences between the measured median FC values and theoretical values are indicated, among which the smallest ones are darkened. Source data are provided as a Source Data file.

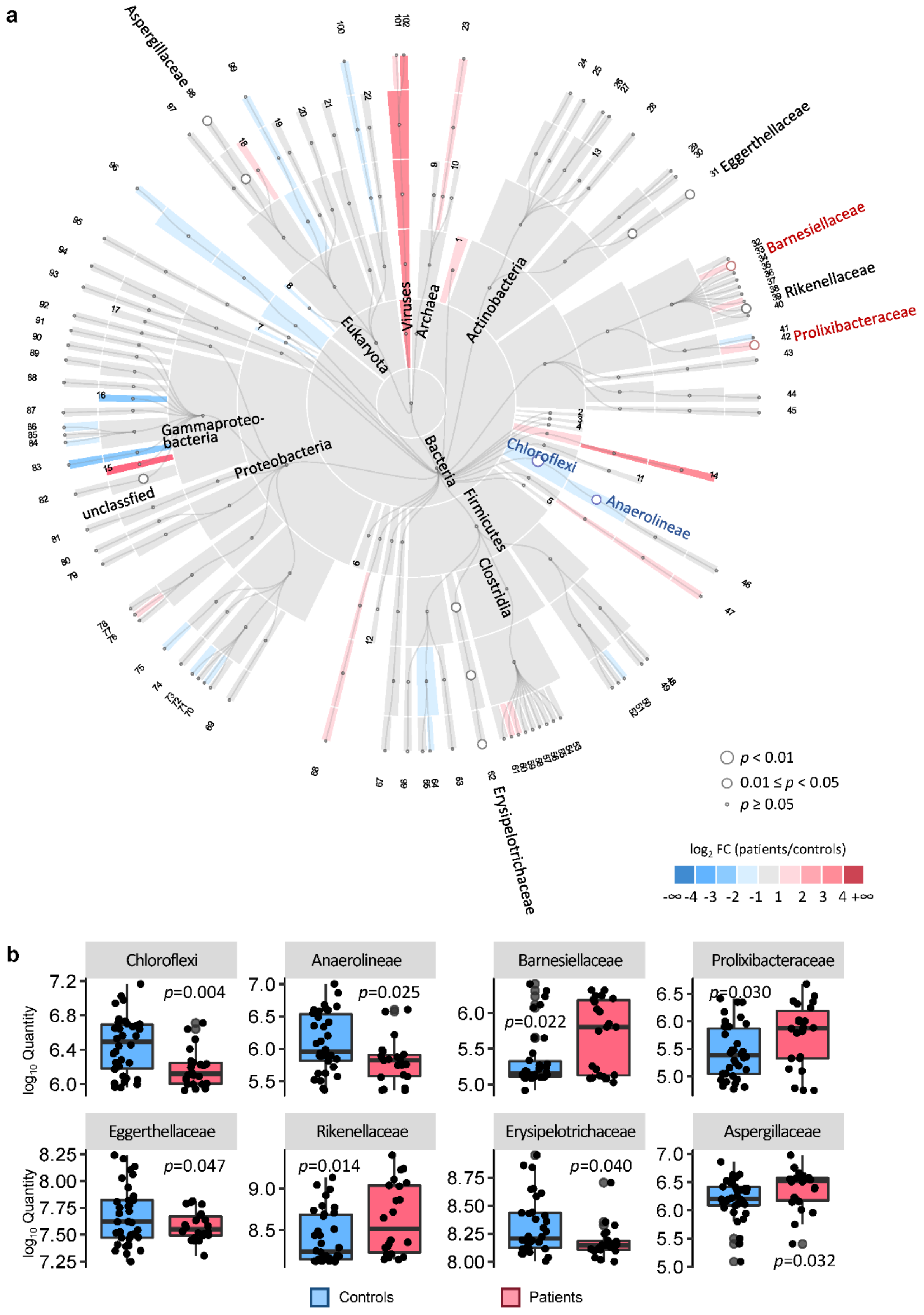
a
Aspergillaceae
Eggerthellaceae
Barnesiellaceae
Rikenellaceae
Prolixibacteraceae
Viruses
Archaea
Eukaryota
Actinobacteria
Bacteria
Chloroflexi
Anaerolineae
Firmicutes
Clostridia
Erysipelotrichaceae
Proteobacteria
Gammaproteo-
bacteria
unclassfied
p < 0.01
0.01 ≤ p < 0.05
p ≥ 0.05
log2 FC (patients/controls)
-∞ -4 -3 -2 -1 1 2 3 4 +∞
b
Chloroflexi
p=0.004
Anaerolineae
p=0.025
Barnesiellaceae
p=0.022
Prolixibacteraceae
p=0.030
Eggerthellaceae
p=0.047
Rikenellaceae
p=0.014
Erysipelotrichaceae
p=0.040
Aspergillaceae
p=0.032
log10 Quantity
Controls
Patients

Supplementary Figure 28. Taxonomic abundances based on microbial proteins of the MCI patients and controls.

(**a**) Cladogram illustrating abundance of taxa (domain to family). Colors indicate the $\log_2$ fold change (FC, patients/controls) between the patients and controls; circle sizes indicate the p-value (t-test). Names of the taxa are highlighted in red (more abundant in the patients) or blue (more abundant in the controls) color if their significant abundance differences between patients and controls were observed (p-value < 0.05). Information of the taxa with the labeled numbers are shown in **Supplementary Data 12**. (**b**) Boxplots showing the abundance of the differential taxa between the patients and controls. The boxes mark the first and third quantile and the lines inside the boxes mark the median; the whiskers extend from the ends of the inter-quartile range (IQR) to the furthest observations within the 1.5 times the IQR. Individual data points are overlaid as dots. The p-values are indicated. Source data are provided as a Source Data file.

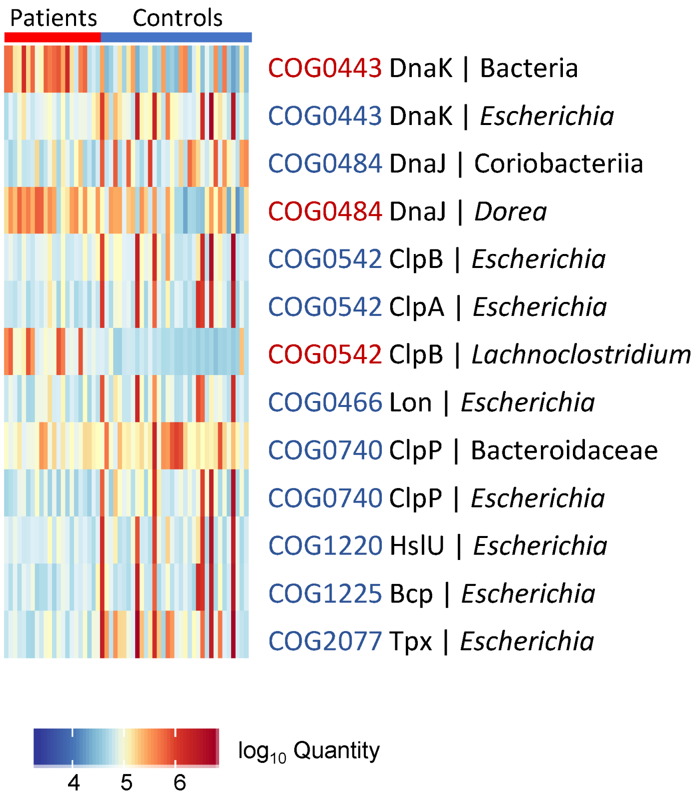

Supplementary Figure 29. Heatmap showing the stress response-related gut microbial chaperones, proteases and peroxidases that are significantly changed in the MCI patients. COG numbers are highlighted in red if they are more abundant in the patients compared to the controls, or conversely in blue (p-value < 0.05, MS1-MS2-combined statistical test in Spectronaut, adjusted using the Bonferroni method). Source data are provided as a Source Data file.

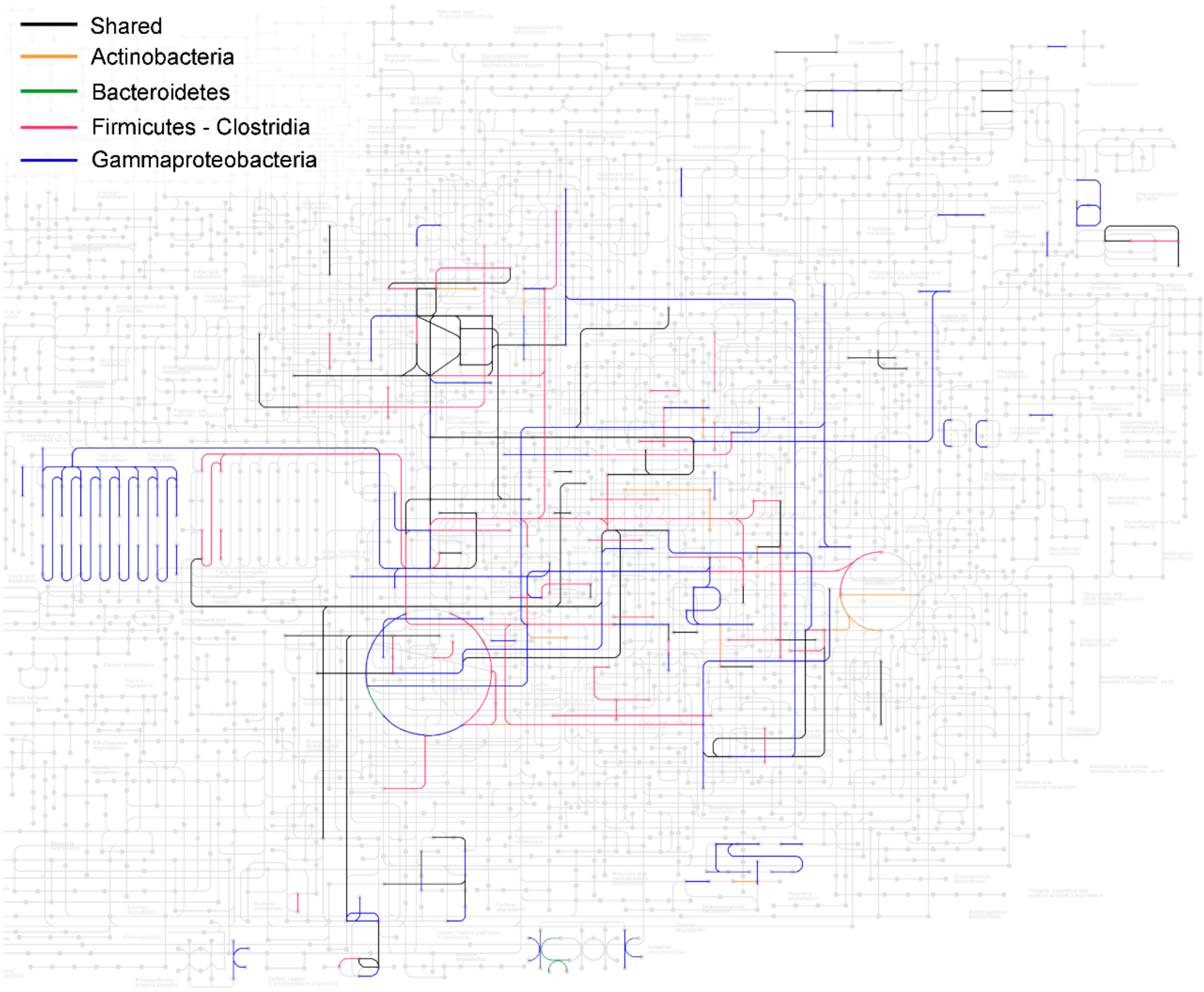

Supplementary Figure 30. Distribution of enriched bacterial metabolic pathways in the MCI patients based on the differential proteins.

Enzymes and metabolic reactions shared among multiple taxa are colored in black, while other colors indicate metabolic reactions only enriched in a specific taxon. Pathway map was adapted from the KEGG pathway map01100. Source data are provided as a Source Data file.

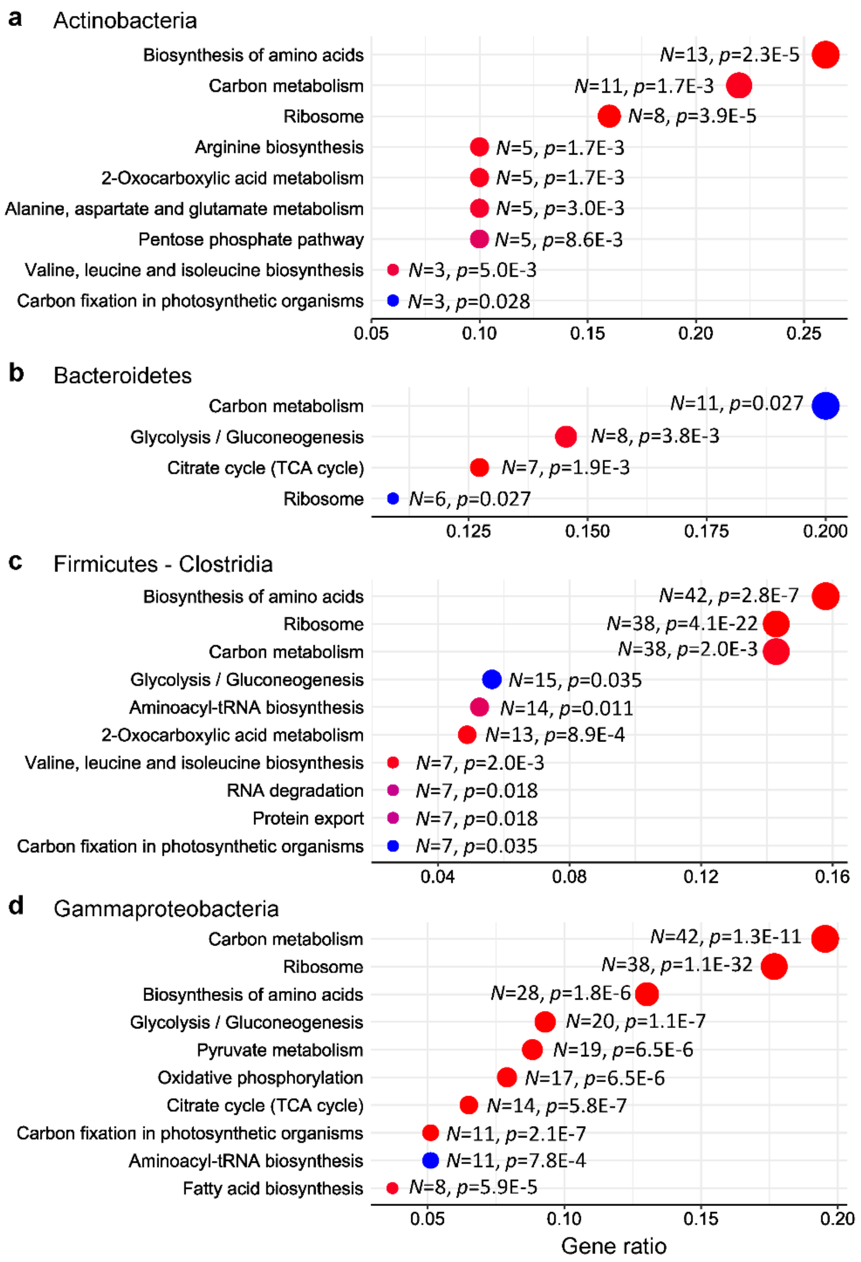

Supplementary Figure 31. Enriched bacterial metabolic pathways in the MCI patients based on the differential proteins.

(**a**) Enriched pathways of the phylum Actinobacteria. (**b**) Enriched pathways of the phylum Bacteroidetes. (**c**) Enriched pathways of the class Clostridia in the phylum Firmicutes. (**d**) Enriched pathways of the class Gammaproteobacteria in the phylum Proteobacteria. Numbers of KO entries (*N*) and adjusted p-values (hypergeometric test, adjusted using the Benjamini-Hochberg method) are indicated. Gene ratio: number of the differential genes related to the pathway / number of the total differential genes.

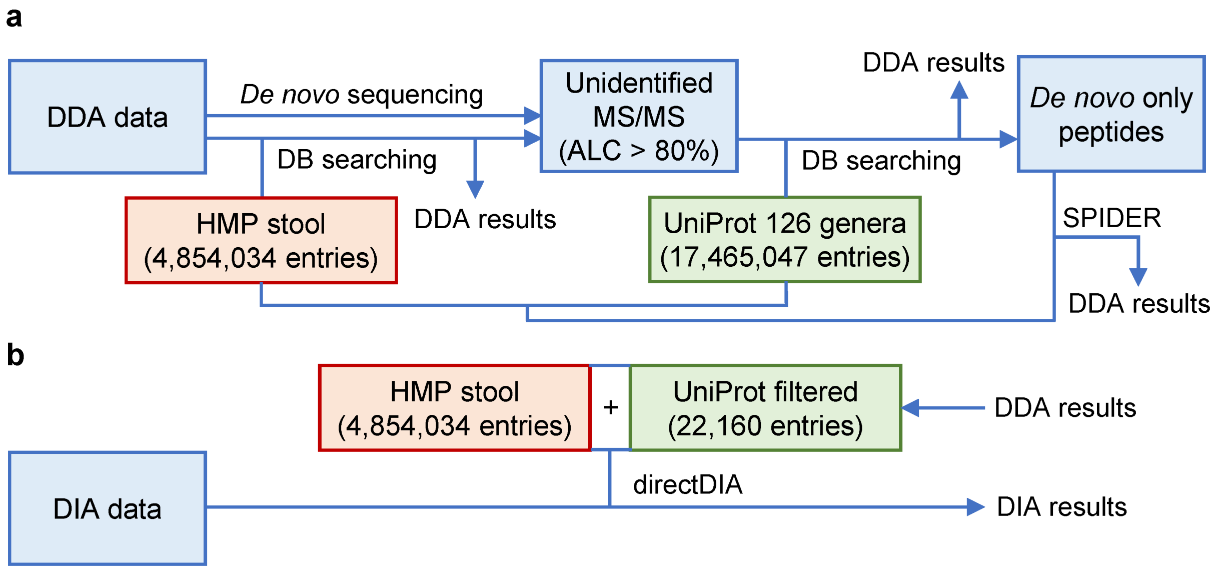

Supplementary Figure 32. Workflow of DIA data analysis using taxonomy-based database. (**a**) De novo sequencing assisted database searching by PEAKS on the pooled and fractionated DDA data against successively the database of stool microbial proteins from Human Microbiome Project (HMP) and a database combining the proteomes from UniProt of the genera identified by 16S rRNA gene sequencing. SPIDER matching was applied on the de novo only results. (**b**) DIA data analysis by directDIA against a database combining the HMP and the identified UniProt proteins in (**a**). ALC: average local confidence.

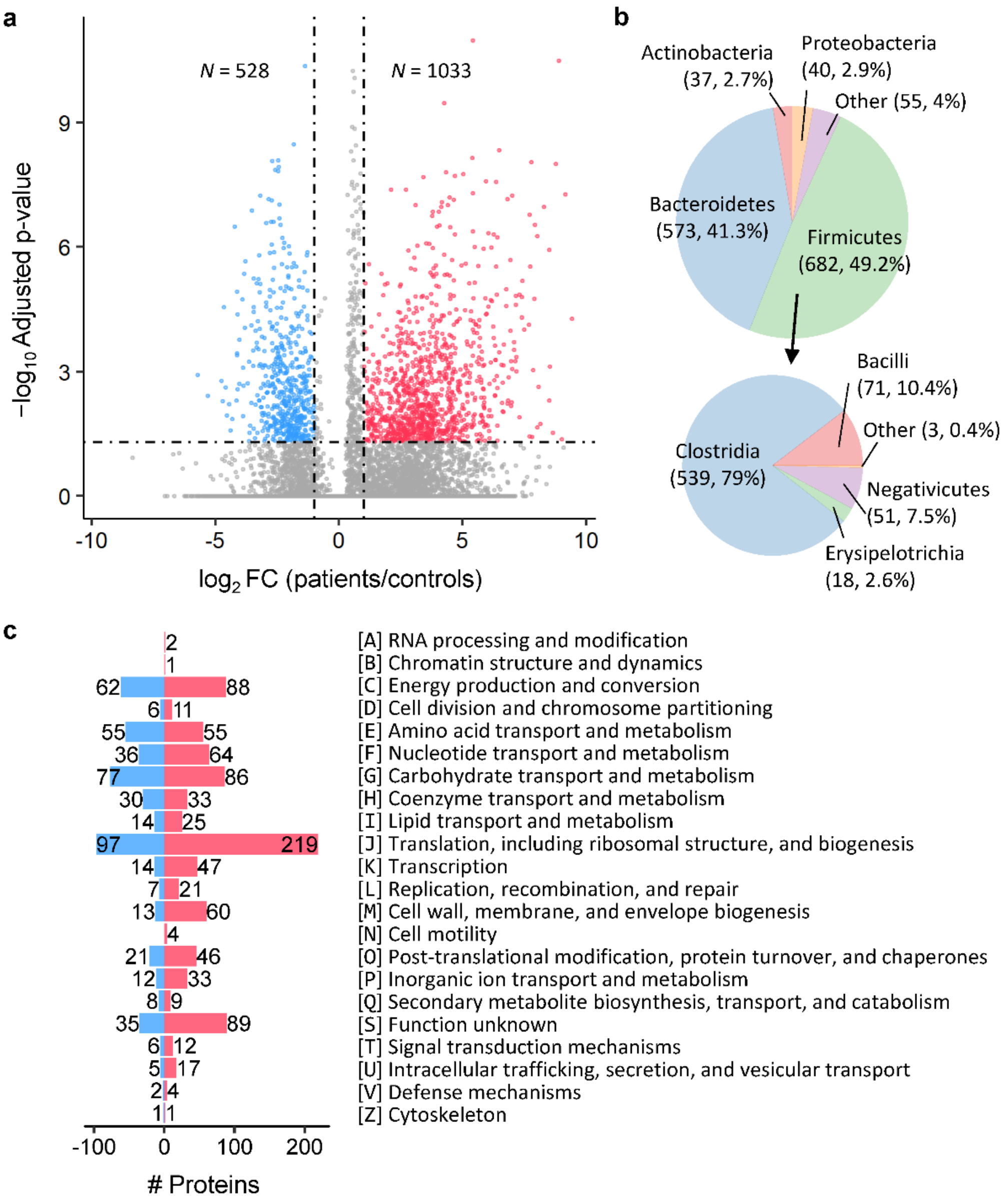

Supplementary Figure 33. Differential gut microbial proteins between the PC patients and controls.

(**a**) Volcano plot indicating the differential proteins between the PC patients and controls. Proteins with fold change (FC, patients/controls) > 2 and p-value < 0.05 were colored red, while those with FC < 0.5 and p-value < 0.05 were colored blue. The p-values were given by MS1-MS2-combined statistical test of Spectronaut and adjusted by the Bonferroni method. (**b**) Distribution of taxonomy assigned to the differential proteins. (**c**) Numbers of the differential proteins in each category of clusters of orthologous groups (COG). Proteins more abundant in patients were colored red, while those more abundant in controls were colored blue.

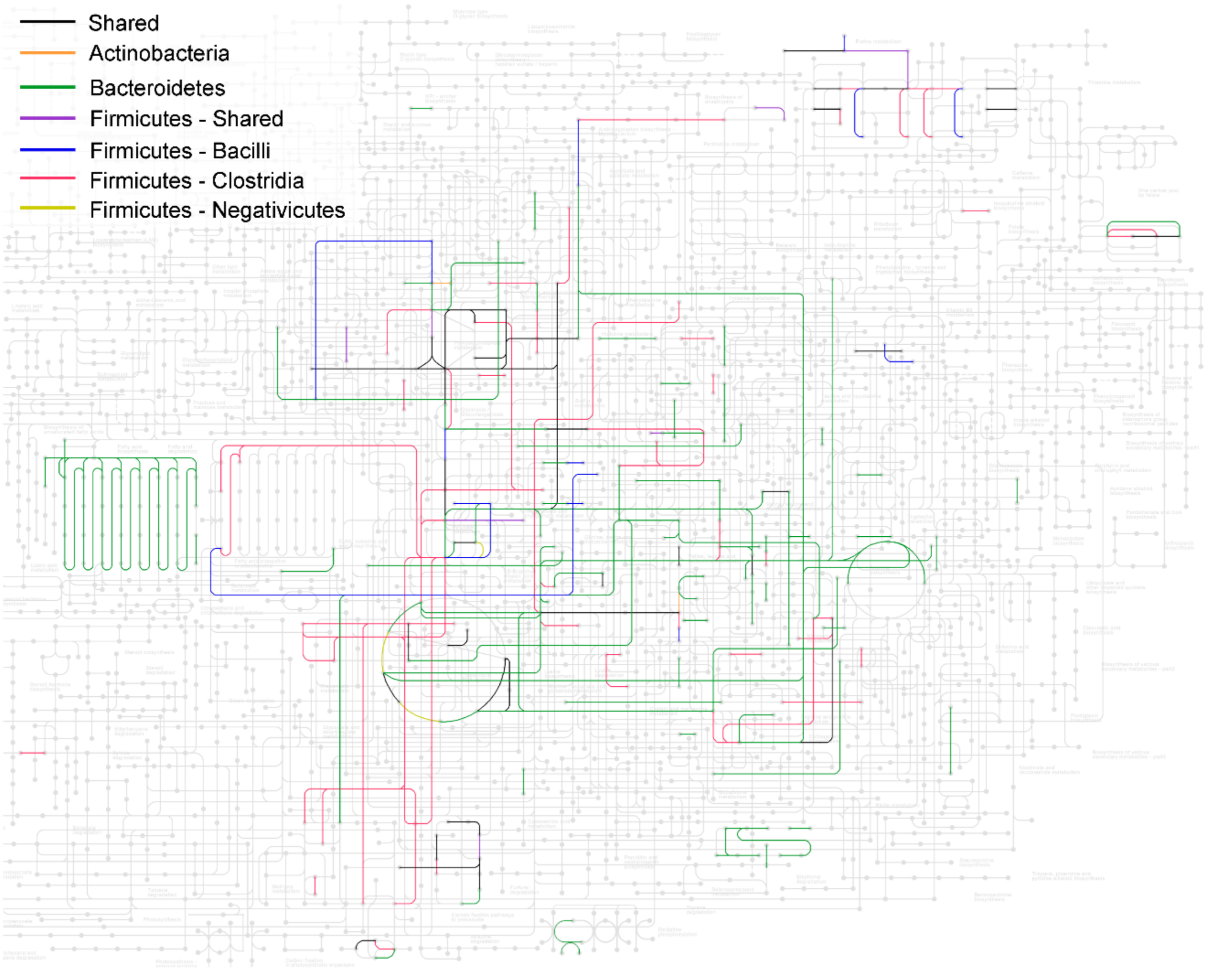

Supplementary Figure 34. Distribution of enriched bacterial metabolic pathways in the PC patients based on the differential proteins.

Enzymes and metabolic reactions shared among multiple taxa are colored in black, while other colors indicate metabolic reactions only enriched in a specific taxon. Pathway map was adapted from the KEGG pathway map01100. Source data are provided as a Source Data file.

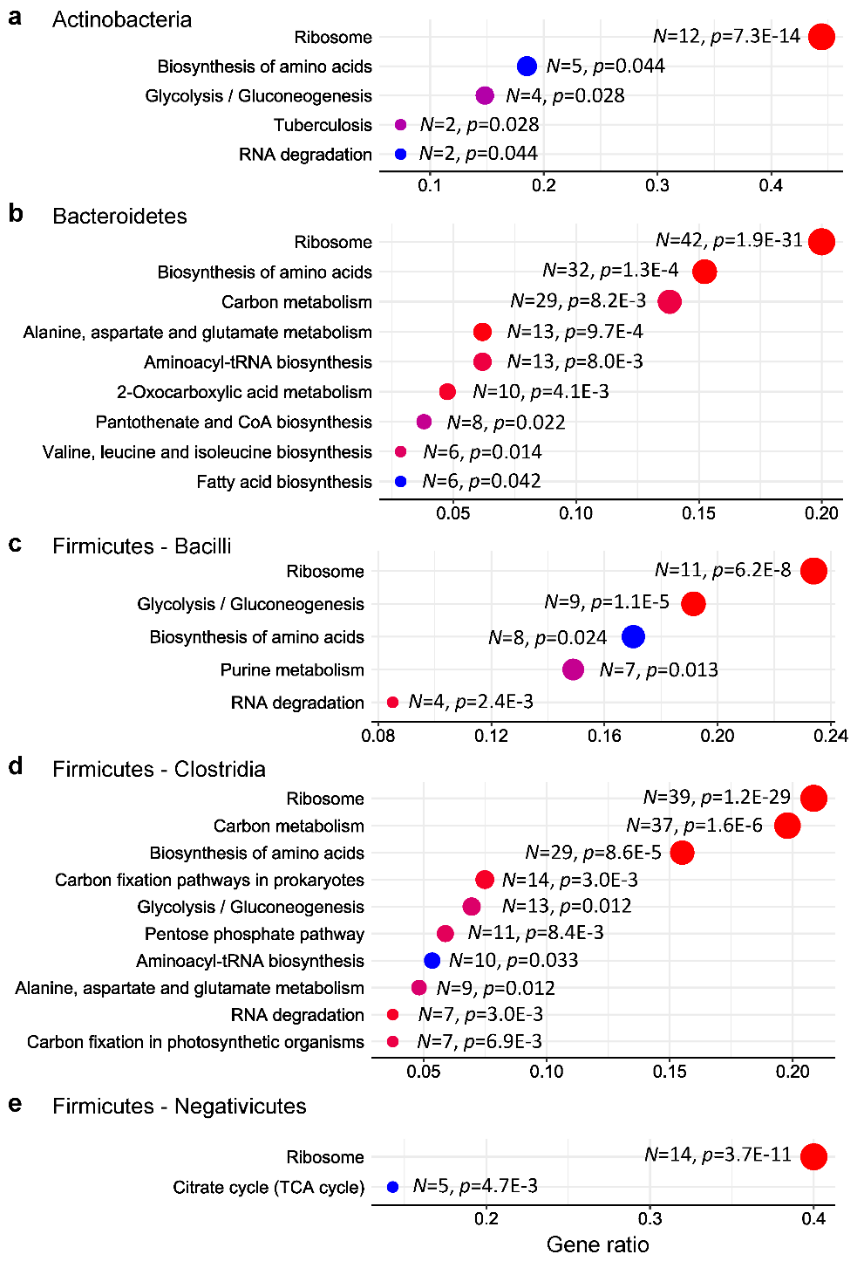

Supplementary Figure 35. Enriched bacterial metabolic pathways in the PC patients based on the differential proteins.

(**a**) Enriched pathways of the phylum Actinobacteria. (**b**) Enriched pathways of the phylum Bacteroidetes. (**c**) Enriched pathways of the class Bacilli in the phylum Firmicutes. (**d**) Enriched pathways of the class Clostridia in the phylum Firmicutes. (**e**) Enriched pathways of the class Negativicutes in the phylum Firmicutes. Numbers of KO entries (*N*) and adjusted p-values (hypergeometric test, adjusted using the Benjamini-Hochberg method) are indicated. Gene ratio: number of the differential genes related to the pathway / number of the total differential genes.

Supplementary Table 1. The bacterial strains used for constructing the simulated microbial communities and the corresponding culture conditions.

Strain	Source	Cultivation condition
Escherichia coli ATCC 25922	ATCC	TSB 37 °C, shaking, aerobic
Citrobacter freundii CICC 10404	CICC	TSB 37 °C, shaking, aerobic
Enterococcus casseliflavus ATCC 700327	ATCC	BHI 37 °C, shaking, aerobic
Enterococcus faecalis ATCC 19433	ATCC	BHI 37 °C, shaking, aerobic
Pseudomonas aeruginosa ATCC 27853	ATCC	TSB 37 °C, shaking, aerobic
Enterobacter asburiae ATCC 35953	ATCC	TSB 30 °C, shaking, aerobic
Klebsiella aerogenes ATCC 13048	ATCC	TSB 30 °C, shaking, aerobic
Klebsiella pneumonia ATCC 4352	ATCC	TSB 30 °C, shaking, aerobic
Morganella morganii CICC 21517	CICC	TSB 30 °C, shaking, aerobic
Bacteroides fragilis ATCC 25285	CICC	BHI 37 °C, shaking, anaerobic
Lactobacillus acidophilus CICC 6074	CICC	MRS 37 °C, shaking, anaerobic
Clostridium butyricum ATCC 1939	CICC	MRS 37 °C, shaking, anaerobic

ATCC: American Type Culture Collection; CICC: China Center of Industrial Culture Collection; TSB: tryptic soy broth; BHI: brain heart infusion; MRS: De Man, Rogosa and Sharpe agar.

Supplementary Table 2. The composition of the simulated communities with 12 bacterial species.

Species	Cell number (10^8 CFU)		
	S1	S2	S3
Morganella morganii	20	4	8
Pseudomonas aeruginosa	4	8	20
Klebsiella pneumonia	20	4	8
Citrobacter freundii	4	10	2
Enterococcus faecalis	2	4	10
Klebsiella aerogenes	2	4	10
Bacteroides fragilis	2	10	4
Enterobacter asburiae	10	2	4
Enterococcus casseliflavus	4	10	2
Escherichia coli	1.32	2	0.68
Clostridium butyricum	0.067	0.133	0.333
Lactobacillus acidophilus	0.333	0.133	0.067

S1: sample 1; S2: sample 2; S3: sample 3.

The cell numbers of *Clostridium butyricum* and *Lactobacillus acidophilus* were significantly lower than the other species, because the cell sizes of the two species are significantly larger than the others and significant higher amounts of proteins can be extracted from one cell of the two species compared to the others.

Supplementary Table 3. The correlation between raw file names and the sample compositions of the simulated microbial communities.

Dataset	Raw files		
Mix12<br>LFQ-DDA	S1:<br>YS-194-1-1.raw<br>YS-194-1-2.raw<br>YS-194-1-3.raw	S2:<br>YS-194-2-1.raw<br>YS-194-2-2.raw<br>YS-194-2-3.raw	S3:<br>YS-194-3-1.raw<br>YS-194-3-2.raw<br>YS-194-3-3.raw
Mix12<br>TMT	YS-QiaoLiang-TMT-MS3-F*.raw (16 fractions)		
	S1: 126, 127N, 127C	S2: 128C, 129N, 129C	S3: 130N, 130C, 131
Mix12<br>LFQ-DIA	S1:<br>YS-194-DIA-1.raw<br>YS-194-DIA-<br>1_20200620233957.raw<br>YS-194-DIA-<br>1_20200621022500.raw	S2:<br>YS-194-DIA-2.raw<br>YS-194-DIA-<br>2_20200621094425.raw<br>YS-194-DIA-<br>2_20200621122928.raw	S3:<br>YS-194-DIA-3.raw<br>YS-194-DIA-<br>3_20200621194833.raw<br>YS-194-DIA-<br>3_20200621223326.raw

S1: sample 1; S2: sample 2; S3: sample 3.

Supplementary Table 4. The composition of the three samples with 6 bacterial species.

Species	Cell number (10^8 CFU)		
	S1	S2	S3
Morganella morganii	20	4	8
Pseudomonas aeruginosa	4	8	20
Enterococcus faecalis	2	10	4
Enterobacter asburiae	10	4	2
Clostridium butyricum	0.267	0.667	0.133
Lactobacillus acidophilus	0.267	0.133	0.667

S1: sample 1; S2: sample 2; S3: sample 3.

The cell numbers of *Clostridium butyricum* and *Lactobacillus acidophilus* were significantly lower than the other species, because the cell sizes of the two species are significantly larger than the others and significant higher amounts of proteins can be extracted from one cell of the two species compared to the others.

After protein extraction from the 6-species mixtures, 1 μg of the bacterial proteins were taken and spiked into 99 μg of microbial proteins extracted from the fecal sample to form the spiked samples.

Supplementary Table 5. The correlation between raw file names and the compositions of the spike-in metaproteome samples.

<table>
<tr><th>Dataset</th><th colspan="3">Raw files</th></tr>
<tr><td>FecesMix6
LFQ-DDA</td><td>S1:
Qiao-FB1-DDA-1.raw
Qiao-FB1-DDA-2.raw
Qiao-FB1-DDA-3.raw</td><td>S2:
Qiao-FB2-DDA-1.raw
Qiao-FB2-DDA-2.raw
Qiao-FB2-DDA-3.raw</td><td>S3:
Qiao-FB3-DDA-1.raw
Qiao-FB3-DDA-2.raw
Qiao-FB3-DDA-3.raw</td></tr>
<tr><td rowspan="2">FecesMix6
TMT</td><td colspan="3">Qiao-TMT-MS3-DDA-F*.raw (12 fractions)</td></tr>
<tr><td>S1: 126, 127N, 127C</td><td>S2: 128N, 128C, 129N</td><td>S3: 129C, 130N, 130C</td></tr>
<tr><td>FecesMix6
LFQ-DIA</td><td>S1:
Qiao-FB1-DIA-1.raw
Qiao-FB1-DIA-2.raw
Qiao-FB1-DIA-3.raw</td><td>S2:
Qiao-FB2-DIA-1.raw
Qiao-FB2-DIA-2.raw
Qiao-FB2-DIA-3.raw</td><td>S3:
Qiao-FB3-DIA-1.raw
Qiao-FB3-DIA-2.raw
Qiao-FB3-DIA-3.raw</td></tr>
</table>

S1: sample 1; S2: sample 2; S3: sample 3.

Supplementary Table 6. Demographic data of the MCI patients and cognitively normal controls.

Characteristics	MCI patients (*n* = 22)	Controls (*n* = 34)
Age, year, mean ± SD	66 ± 2.87	69 ± 4.05
Gender, male + female, number (%)	8 (36.37) + 14 (63.63)	19 (54.29) + 15 (45.71)
Education, year, mean ± SD	9 ± 2.76	11 ± 3.17
MoCA scores, mean ± SD	21 ± 2.57	25 ± 2.21

SD: standard deviation; MoCA: Montreal cognitive assessment.

Supplementary Table 7. Demographic data of the PC patients and cognitively normal controls.

Characteristics	PC patients (*n* = 15)	Controls (*n* = 15)
Age, year, mean ± SD	58 ± 6.75	56 ± 7.23
Gender, male + female, number (%)	9 (60) + 6 (40)	9 (60) + 6 (40)

SD: standard deviation.

Supplementary Table 8. LC gradients.

HPRP-LC		LC-MS/MS	
Time (h:mm)	B (%)	Time (h:mm)	B (%)
0:00	0	0:00	2
5:00	5	3:00	2
50:00	35	96:00	17
60:00	50	111:00	27
65:00	100	116:00	37
75:00	100	119:00	50
		121:00	95
		130:00	100

Supplementary Table 9. DIA variable window settings.

Window center (m/z)	Window width (m/z)	Window center (m/z)	Window width (m/z)
355.5	112	754.5	14
426.5	32	768.5	16
450.5	18	783	15
469	21	796.5	14
489.5	22	811	17
509	19	825.5	14
526.5	18	837.5	12
543	17	849	13
557	13	863.5	18
569.5	14	880.5	18
581.5	12	895.5	14
589.5	6	909	15
595.5	8	923.5	16
604	11	938.5	16
614.5	12	955	19
624	9	973	19
634.5	14	991.5	20
646.5	12	1010	19
658.5	14	1036	35
672.5	16	1063	21
686.5	14	1091	37
699.5	14	1125.5	34
712.5	14	1168.5	54
726.5	16	1232.5	76
741	15	1309.5	80

Supplementary Note 1. Comparison of software solutions for LFQ-DDA data analysis on the 12-species samples.

We compared several state-of-the-art software solutions, including PEAKS Studio[1], MaxQuant[2], and FragPipe (MSFragger[3]), for LFQ-DDA data analysis of the simulated microbial community of 12 species. The quantification results of proteins and peptides are shown in **Supplementary Data 1**. As shown in **Supplementary Figures 2** and **3**, PEAKS Studio identified and quantified 10,240 ± 185 (mean ± standard deviation, sic passim) proteins and 49,018 ± 1539 peptides per run. From the 9 runs, 11,361 proteins and 57,847 peptides were detected totally, among which 71% (8114) proteins and 58% (33,679) peptides were shared in all the runs. PEAKS Studio yielded more detected proteins/peptides and fewer missing values compared with MaxQuant (6392 ± 207 proteins and 42315 ± 1660 peptides per run; 52% [4183/8057] proteins and 35% [20,513/59,003] peptides shared in all the runs) and FragPipe (6539 ± 356 proteins and 41,987 ± 2506 peptides per run; 41% [3763/9146] proteins and 28% [18,096/64,926] peptides shared in all the runs).

For the proteins and peptides detected in all the 3 replicate runs in each sample group, coefficient of variation (CV) values of quantification were calculated among three replicates. FragPipe resulted in smaller protein-level and peptide-level CV values than PEAKS Studio and MaxQuant (**Supplementary Figure 4**), indicating the good quantification precision of this software tool.

We also calculated the fold change (FC) values of quantification results between each two of the three samples based on the average of the replicates of each sample (**Supplementary Figure 5**). Only the proteins detected in at least 2/3 replicate runs in each sample group and uniquely belonging to one species were taken into consideration. The experimental median FC values were compared with the theoretical values. Among the 36 comparisons (by pairwise enumeration of the 3 samples as numerator and denominator) of the 12 species, PEAKS Studio achieved the most accurate measurement in 19 comparisons, indicating better quantitative accuracy than MaxQuant and FragPipe.

Supplementary Note 2. Comparison of software solutions for TMT data analysis on the 12-species samples.

PEAKS Studio[1], MaxQuant[2], and FragPipe (MSFragger[3]) were compared for TMT data analysis of the simulated microbial community consisting of 12 species. The quantification results of proteins and peptides are shown in **Supplementary Data 2**. As shown in **Supplementary Figures 6** and **7**, FragPipe achieved more detected proteins/peptides but slightly more missing values (14,423 ± 110 proteins and 67,091 ± 1591 peptides per channel; 88% [13,181/15,029] proteins and 70% [52,337/74,400] peptides with reporter ion signals detected in all the channels) compared with MaxQuant (13,106 ± 84 proteins and 59,236 ± 1271 peptides per channel; 89% [12,108/13,562] proteins and 73% [47,451/64,890] peptides with reporter ion signals detected in all the channels) and PEAKS Studio (10,810 ± 51 proteins and 56,609 ± 1443 peptides per channel; 92% [10,207/11,081] proteins and 72% [44,716/62,396] peptides with reporter ion signals detected in all the channels).

For the proteins and peptides quantified in all the 3 replicate channels in each sample group, CV values of quantification were calculated among three replicates. The three software tools yielded similar quantification precision (**Supplementary Figure 8**).

FC values of quantification results were calculated using species-specific proteins quantified in at least 2/3 replicate channels in each sample group (**Supplementary Figure 9**). PEAKS Studio achieved the most accurate measurement in 18 of the 36 comparisons, indicating better quantitative accuracy than MaxQuant and FragPipe.

Supplementary Note 3. Quantification of species based on ribosomal proteins.

We searched the description field of all the quantified proteins by directDIA using the keyword "ribosomal" and found 502 result proteins (**Supplementary Data 3**), among which 493 were quantified in at least 2/3 replicate runs in each sample group. Most of the proteins were 30S ribosomal proteins and 50S ribosomal proteins. FC values of quantification results were calculated using those uniquely belonging to one species (**Supplementary Figure 15**). The measured FC distributions of the ribosomal proteins were less dispersed than those of the total proteins. The measured median FC values of the ribosomal proteins were closer to the theoretical values than those of the total proteins in 23 of the 32 comparisons, indicating better quantitative accuracy when considering only ribosomal proteins than total proteins.

Supplementary Note 4. Comparison of software solutions for LFQ-DDA and TMT data analysis on the spike-in metaproteome samples.

PEAKS Studio[1], MaxQuant[2], and FragPipe (MSFragger[3]) were compared for LFQ-DDA and TMT data analysis of the spike-in metaproteome samples. The quantification results of proteins and peptides are shown in **Supplementary Data 4 and 5**.

For LFQ-DDA, PEAKS Studio achieved the highest numbers of detected proteins/peptides (10,229 ± 13 proteins and 47,137 ± 95 peptides per run, **Supplementary Figures 16** and **17**), data completeness (84% [9077/10,774] proteins and 75% [38,542/51,384] peptides detected in all the runs, **Supplementary Figures 16** and **17**), and quantitative accuracy (the most accurate measurement in 10 of the 18 comparisons, **Supplementary Figure 19**).

For TMT, FragPipe achieved the highest numbers of detected proteins/peptides (20,389 ± 14 proteins and 66,459 ± 113 peptides per run, **Supplementary Figures 20** and **21**), whereas MaxQuant showed the highest quantitative accuracy (the most accurate measurement in 15 of the 18 comparisons, **Supplementary Figure 23**). However, ratio compression was observed for all the three software (for PEAKS Studio, −4% to –36% relative error in 8 out of 9 comparisons with theoretical FC > 1, and +18% to +105% relative error in all 9 comparisons with theoretical FC < 1; for MaxQuant, −2% to –33% relative error in 8 out of 9 comparisons with theoretical FC > 1, and +15% to +95% relative error in all 9 comparisons with theoretical FC < 1; for FragPipe, −4% to –37% relative error in 8 out of 9 comparisons with theoretical FC > 1, and +20% to +125% relative error in all 9 comparisons with theoretical FC < 1).

Supplementary Note 5. Characteristics of the taxonomic abundances based on microbial proteins of the MCI patients and controls.

Taxonomic information was assigned to the 233,217 quantified peptides using Unipept[4]. Among them, 93,337 were matched to 80 families of microbes and 80,421 to 141 genera. We summed the quantitative information of all the quantified peptides at different taxonomic levels to demonstrate the abundance of gut microbial taxa (**Supplementary Figure 28** and **Supplementary Data 12**). We observed that the phylum Chloroflexi (FC = 0.456, p-value = 4.2×10^{-3}, t-test, similarly hereinafter) and its subordinate class Anaerolineae (FC = 0.439, p-value = 0.025) were less abundant in the MCI patients, while the families Barnesiellaceae (FC = 2.05, p-value = 0.022) and Prolixibacteraceae (FC = 2.04, p-value = 0.030) were more abundant in the MCI patients. We also observed abundance changes in the families Eggerthellaceae (FC = 0.680, p-value = 0.047), Rikenellaceae (FC = 1.98, p-value = 0.014), Erysipelotrichaceae (FC = 0.638, p-value = 0.040) and Aspergillaceae (FC = 1.49, p-value = 0.032), but their FC values were not significant enough (FC < 0.5 or FC > 2 not satisfied).

Supplementary Note 6. Abundance differences of stress response-related proteins between the MCI patients and controls.

We observed that the stress response-related gut microbial chaperones, proteases and peroxidases were significantly changed in MCI. The chaperones and proteases DnaK, ClpB, ClpA, ClpP, HslU and Lon of *Escherichia*, as well as DnaJ of Coriobacteriia and ClpP of Bacteroidaceae, were less abundance in the MCI patients (**Supplementary Figure 29**). The proteins participant in the degradation of abnormal proteins for maintaining protein homeostasis, playing important roles in several stress responses, including heat shock, DNA damage and oxidative stress[5-11]. The thiol-specific peroxidases Bcp and Tpx of *Escherichia*, which detoxifies peroxides for cell protection against oxidative stress[12], were also decreased in the MCI patients compared to the controls.

Supplementary Note 7. Characteristics of differential proteins between the PC patients and controls.

Differential proteins between the PC patients and controls were determined using abundance FC and statistical test by Spectronaut[13]. The Bonferroni method was conducted on the p-values given by the MS1-MS2-combined statistical test in Spectronaut for multiple testing correction to obtain a conservative result, and 1561 proteins with FC > 2 (or < 0.5) and adjusted p-value < 0.05 were discovered (**Supplementary Figure 33**). In order to assess the microbial functions, the differential proteins were annotated using eggNOG[14]. Among the differential proteins, 1387 were annotated with taxonomy information (**Supplementary Data 10**). The phylum Bacteroidetes (41.3%), as well as the classes Clostridia (38.9%) and Bacilli (5.1%) in the phylum Firmicutes accounted for large proportions of the differential proteins. The differential proteins were annotated into 19 COG categories, where translation (316 proteins in category J), carbohydrate transport and metabolism (163 proteins in category G), as well as energy production and conversion (150 proteins in category C) were among the most significantly altered functions observed in the PC patients.

Supplementary Note 8. Characteristics of the taxonomic abundances based on microbial proteins of the PC patients and controls.

We observed some significant abundance differences in taxa that have been reported in previous studies based on metagenomics or 16S rRNA gene sequencing. Phylum Proteobacteria (FC = 3.63, p-value = 0.037, t-test, similarly hereinafter) showed higher abundance in the PC patients than in the controls, which coincides with previous studies on pancreatic ductal adenocarcinoma (the most common form of PC) using 16S rRNA gene sequencing[15]. Families Porphyromonadaceae (FC = 2.27, p-value = 0.040), Streptococcaceae (FC = 5.22, p-value = 0.045) and Prevotellaceae (FC = 7.98, p-value = 0.030), as well as orders Coriobacteriales (FC = 3.93, p-value = 0.018) and Corynebacteriales (FC = 10.44, p-value = 0.040) were more abundant in the PC patients. Porphyromonas gingivalis, a species in the family Porphyromonadaceae, has been proven as the most prevalent microorganism for periodontal disease. The level of serum immunoglobin G against this microbe was reported positively related to the risk of PC[16,17]. Previous studies using 16S rRNA gene sequencing has demonstrated that species in the genera *Streptococcus* (in Streptococcaceae), *Prevotella* (in Prevotellaceae), *Atopobium* (in Coriobacteriales) and *Corynebacterium* (in Corynebacteriales) were altered in salivary samples between PC and healthy participants[18,19].

The genera *Veillonella* and *Akkermansia* have been observed more abundant in fecal samples of PC patients by 16S rRNA gene sequencing[20]. We observed consistent results for the regulation of the families Veillonellaceae (FC = 6.48, p-value = 0.061) and Akkermansiaceae (FC = 9.08, p-value = 0.166) based on the quantitative metaproteomics, while the difference of their abundance was not statistically significant enough (p-value > 0.05).

We also observed changes in taxonomic abundance that have not been revealed by metagenomics and 16S rRNA, including the families Piscirickettsiaceae (FC = 0.174, p-value = 0.006) and Phyllobacteriaceae (FC = 0.327, p-value = 0.039).

We notice that the 16S rRNA gene sequencing result contained only the 126 genera of bacteria and archaea. In our workflow, UniProt sequences of these genera were used as a complement to the HMP database and the latter contained sequences from eukaryotes and food. Notably, taxonomic information was assigned to peptides using the lowest common ancestor approach, rather than using the taxon annotation of UniProt proteins directly. A peptide shared between bacteria and eukaryotes/food would not be assigned to any taxa but the “root” node.

Supplementary References

1. Lin, H., He, L. & Ma, B. A combinatorial approach to the peptide feature matching problem for label-free quantification. *Bioinformatics* **29**, 1768-1775 (2013).
2. Tyanova, S., Temu, T. & Cox, J. The MaxQuant computational platform for mass spectrometry-based shotgun proteomics. *Nat. Protoc.* **11**, 2301-2319 (2016).
3. Kong, A. T., Leprevost, F. V., Avtonomov, D. M., Mellacheruvu, D. & Nesvizhskii, A. I. MSFragger: ultrafast and comprehensive peptide identification in mass spectrometry–based proteomics. *Nat. Methods* **14**, 513-520 (2017).
4. Gurdeep Singh, R. et al. Unipept 4.0: functional analysis of metaproteome data. *J. Proteome Res.* **18**, 606-615 (2019).
5. Schröder, H., Langer, T., Hartl, F. U. & Bukau, B. DnaK, DnaJ and GrpE form a cellular chaperone machinery capable of repairing heat-induced protein damage. *EMBO J.* **12**, 4137-4144 (1993).
6. Zolkiewski, M., Zhang, T. & Nagy, M. Aggregate reactivation mediated by the Hsp100 chaperones. *Arch. Biochem. Biophys.* **520**, 1-6 (2012).
7. Hoskins, J. R., Singh, S. K., Maurizi, M. R. & Wickner, S. Protein binding and unfolding by the chaperone ClpA and degradation by the protease ClpAP. *Proc. Natl. Acad. Sci.* **97**, 8892 (2000).
8. Kim, Y.-I. et al. Molecular determinants of complex formation between Clp/Hsp100 ATPases and the ClpP peptidase. *Nat. Struct. Biol.* **8**, 230-233 (2001).
9. Rohrwild, M. et al. HslV-HslU: a novel ATP-dependent protease complex in Escherichia coli related to the eukaryotic proteasome. *Proc. Natl. Acad. Sci.* **93**, 5808 (1996).
10. Gottesman, S. Proteases and their targets in Escherichia coli. *Annu. Rev. Genet.* **30**, 465-506 (1996).
11. Yan, Z. et al. A semi-tryptic peptide centric metaproteomic mining approach and its potential utility in capturing signatures of gut microbial proteolysis. *Microbiome* **9**, 12 (2021).
12. Tao, K. Subcellular localization and in vivo oxidation–reduction kinetics of thiol peroxidase in Escherichia coli. *FEMS Microbiol. Lett.* **289**, 41-45 (2008).
13. Huang, T. et al. Combining precursor and fragment information for improved detection of differential abundance in data independent acquisition. *Mol. Cell. Proteom.* **19**, 421-430 (2020).
14. Huerta-Cepas, J. et al. eggNOG 5.0: a hierarchical, functionally and phylogenetically annotated orthology resource based on 5090 organisms and 2502 viruses. *Nucleic Acids Res.* **47**, D309-D314 (2019).
15. Pushalkar, S. et al. The pancreatic cancer microbiome promotes oncogenesis by induction of innate and adaptive immune suppression. *Cancer Discov.* **8**, 403 (2018).
16. Ahn, J., Segers, S. & Hayes, R. B. Periodontal disease, Porphyromonas gingivalis serum antibody levels and orodigestive cancer mortality. *Carcinogenesis* **33**, 1055-1058 (2012).
17. Li, Q., Jin, M., Liu, Y. & Jin, L. Gut microbiota: its potential roles in pancreatic cancer. *Front. Cell. Infect. Microbiol.* **10** (2020).
18. Farrell, J. J. et al. Variations of oral microbiota are associated with pancreatic diseases including pancreatic cancer. *Gut* **61**, 582 (2012).
19. Lin, I. H. et al. Abstract 101: Pilot study of oral microbiome and risk of pancreatic cancer. *Cancer Res.* **73**, 101 (2013).

20. Half, E. et al. P-165 Specific changes in fecal microbiota may differentiate pancreatic cancer patients from healthy individuals. *Ann. Oncol.* **26**, iv48 (2015).
