# Supplementary material for: Data-independent acquisition boosts quantitative metaproteomics for deep characterization of gut microbiota
Source: NPJ Biofilms Microbiomes. 2023 Jan 24;9:4. doi: 10.1038/s41522-023-00373-9 (PMC9873935; doi:10.1038/s41522-023-00373-9)
Supplement: Supplementary file 2 — Reporting Summary [file 41522_2023_373_MOESM2_ESM.pdf]

Corresponding author(s): Liang Qiao

Last updated by author(s): Nov 15, 2022

Reporting Summary

Nature Research wishes to improve the reproducibility of the work that we publish. This form provides structure for consistency and transparency in reporting. For further information on Nature Research policies, see our Editorial Policies and the Editorial Policy Checklist.

Statistics

For all statistical analyses, confirm that the following items are present in the figure legend, table legend, main text, or Methods section.

n/a	Confirmed	
☐	☒	The exact sample size (*n*) for each experimental group/condition, given as a discrete number and unit of measurement
☐	☒	A statement on whether measurements were taken from distinct samples or whether the same sample was measured repeatedly
☐	☒	The statistical test(s) used AND whether they are one- or two-sided<br>*Only common tests should be described solely by name; describe more complex techniques in the Methods section.*
☒	☐	A description of all covariates tested
☐	☒	A description of any assumptions or corrections, such as tests of normality and adjustment for multiple comparisons
☐	☒	A full description of the statistical parameters including central tendency (e.g. means) or other basic estimates (e.g. regression coefficient) AND variation (e.g. standard deviation) or associated estimates of uncertainty (e.g. confidence intervals)
☐	☒	For null hypothesis testing, the test statistic (e.g. *F*, *t*, *r*) with confidence intervals, effect sizes, degrees of freedom and *P* value noted<br>*Give P values as exact values whenever suitable.*
☒	☐	For Bayesian analysis, information on the choice of priors and Markov chain Monte Carlo settings
☒	☐	For hierarchical and complex designs, identification of the appropriate level for tests and full reporting of outcomes
☒	☐	Estimates of effect sizes (e.g. Cohen's *d*, Pearson's *r*), indicating how they were calculated

Our web collection on statistics for biologists contains articles on many of the points above.

Software and code

Policy information about availability of computer code

Data collection	Data collection was performed by Thermo Xcalibur (version 3.0.63).
Data analysis	Raw DDA data were analyzed by PEAKS Studio (version X+, Bioinformatics Solutions Inc., Waterloo, Canada), MaxQuant (version 2.0.3.0), and FragPipe (version 17.1). Raw DIA data were analyzed by Spectronaut (version 15.4.210913 and 16.2.220903, Biognosys AG, Schlieren, Switzerland) and DIA-NN (version 18.0). Annotation of the differential proteins was performed with eggNOG (version 5.0, http://eggnogdb.embl.de/, accessed in November 2021). KEGG enrichment analysis was perform using the R package “clusterProfiler” (version 4.2.0). The quantified peptides were subjected to Unipept (version 4.3, https://unipept.ugent.be/, accessed in November 2021) for taxonomic analysis. The peptide-level taxonomic analysis was performed using Python (version 3.5.6, Anaconda distribution version 4.2.0, https://www.anaconda.com/). Other statistics was conducted using R (version 3.5.1 and 4.0.2, Microsoft R Open distribution, https://mran.microsoft.com/open). AntV G2 (version 3.2.7, https://g2.antv.vision/) was used to plot the cladograms illustrating protein abundance of taxa. The R packages “ggplot2” (version 3.0.0) and “VennDiagram” (version 1.6.20) were used for other data visualization.<br>Custom scripts for post-analysis data processing and visualization in this paper are available at Github (https://github.com/lmsac/metaproteomics-utilities).

For manuscripts utilizing custom algorithms or software that are central to the research but not yet described in published literature, software must be made available to editors and reviewers. We strongly encourage code deposition in a community repository (e.g. GitHub). See the Nature Research guidelines for submitting code & software for further information.

Data

Policy information about availability of data

All manuscripts must include a data availability statement. This statement should provide the following information, where applicable:

- Accession codes, unique identifiers, or web links for publicly available datasets
- A list of figures that have associated raw data
- A description of any restrictions on data availability

All raw MS data, spectral libraries and search results generated in this study have been deposited to the ProteomeXchange via the iProX72 partner repository with accession numbers PXD031301 or IPX0003851000. Public proteome databases used in this study are available at UniProt (https://www.uniprot.org/) and HMP (https://hmpdacc.org/), and custom databases have been deposited to the ProteomeXchange/iProX repository. The source data underlying all figures including statistics are provided as a Source Data file.

Field-specific reporting

Please select the one below that is the best fit for your research. If you are not sure, read the appropriate sections before making your selection.

☒ Life sciences ☐ Behavioural & social sciences ☐ Ecological, evolutionary & environmental sciences

For a reference copy of the document with all sections, see nature.com/documents/nr-reporting-summary-flat.pdf

Life sciences study design

All studies must disclose on these points even when the disclosure is negative.

Sample size	When benchmarking the quantification methods, each sample was analyzed with 3 replicates. The sample size is usually used in proteomics study to validate the technical variance.<br>When applying the DIA method to clinical studies, 15 PC patients and 15 non-PC volunteers, as well as 22 MCI patients and 34 non-MCI volunteers were enrolled. No statistical methods were used to pre-determine the sample size.
Data exclusions	No data were excluded.
Replication	When benchmarking the quantification methods, each sample was analyzed with 3 replicates to verify the quantification results. All attempts at replication were successful.<br>When applying the DIA method to clinical studies, we have not re-run the study with another independent set of participants. The findings about abundance differences in taxa and protein functions have been partially reported in previous studies.
Randomization	When benchmarking the quantification methods, no randomization was carried out.<br>When applying the DIA method to clinical studies, the participants were randomly recruited.
Blinding	The investigators were not blinded to the conditions within participants. Data analysis were performed using computational pipeline applied equally to all conditions of the samples.

Reporting for specific materials, systems and methods

We require information from authors about some types of materials, experimental systems and methods used in many studies. Here, indicate whether each material, system or method listed is relevant to your study. If you are not sure if a list item applies to your research, read the appropriate section before selecting a response.

Materials & experimental systems

n/a	Involved in the study	
☒	☐	Antibodies
☒	☐	Eukaryotic cell lines
☒	☐	Palaeontology and archaeology
☒	☐	Animals and other organisms
☐	☒	Human research participants
☒	☐	Clinical data
☒	☐	Dual use research of concern

Methods

n/a	Involved in the study	
☒	☐	ChIP-seq
☒	☐	Flow cytometry
☒	☐	MRI-based neuroimaging

Human research participants

Policy information about studies involving human research participants

Population characteristics

Population characteristics	Population characteristics of the PC patients and non-PCvolunteers:<br>Age, year, mean ± SD: 58 ± 6.75 56 ± 7.23;<br>Gender, male + female, number (%): 9 (60) + 6 (40) 9 (60) + 6 (40).<br><br>Population characteristics of the MCI patients and non-MCI volunteers:<br>Age, year, mean ± SD: 66 ± 2.87 69 ± 4.05;<br>Gender, male + female, number (%): 8 (36.37) + 14 (63.63) 19 (54.29) + 15 (45.71);<br>Education, year, mean ± SD: 9 ± 2.76 11 ± 3.17;<br>MoCA scores, mean ± SD: 21 ± 2.57 25 ± 2.21.
Recruitment	The participants were randomly recruited.
Ethics oversight	The subjects gave their informed consent for using the biological material for research purposes. The study protocol was approved by the Ethics Committee of Shanghai Changhai Hospital, the Medical Ethics Committee of Shanghai Mental Health Center, and the Ethics Committee of Fudan University, and complied with all relevant laws and regulations of China.

Note that full information on the approval of the study protocol must also be provided in the manuscript.
